# *Staphylococcus aureus* ClpX localizes at the division septum and impacts transcription of genes involved in cell division, T7-secretion, and SaPI5-excision

**DOI:** 10.1038/s41598-019-52823-0

**Published:** 2019-11-11

**Authors:** Camilla Jensen, Marie J. Fosberg, Ida Thalsø-Madsen, Kristoffer T. Bæk, Dorte Frees

**Affiliations:** Department of Veterinary and Animal Sciences, Faculty of Health and Medical Sciences, University of Copenhagen, 1870 Frederiksberg C, Denmark

**Keywords:** Bacterial transcription, Small RNAs, Pathogens, Super-resolution microscopy

## Abstract

In all living cells, molecular chaperones are essential for facilitating folding and unfolding of proteins. ClpX is a highly conserved ATP-dependent chaperone that besides functioning as a classical chaperone can associate with ClpP to form the ClpXP protease. To investigate the relative impact of the ClpXP protease and the ClpX chaperone in cell physiology of the important pathogenic bacterium *Staphylococcus aureus*, we assessed the transcriptional changes induced by inactivating only ClpXP, or by completely deleting ClpX. This analysis revealed that ClpX has a profound impact on *S. aureus* cell physiology that is mediated primarily via ClpXP-dependent pathways. As an example, ClpX impacts expression of virulence genes entirely via ClpXP-dependent mechanisms. Furthermore, ClpX controls a high number of genes and sRNAs via pathways involving both ClpXP protease and ClpX chaperone activities; an interesting example being genes promoting excision and replication of the pathogenicity island SaPI5. Independently of ClpP, ClpX, impacts transcription of only a restricted number of genes involved in peptidoglycan synthesis, cell division, and type seven secretion. Finally, we demonstrate that ClpX localizes in single foci in close proximity to the division septum lending support to the idea that ClpX plays a role in *S. aureus* cell division.

## Introduction

In all living cells protein quality control systems are essential for ensuring correct folding of proteins, and for mitigating the deleterious effects of protein misfolding and aggregation^1^. The task of protein folding is mediated by molecular chaperones that typically use cycles of ATP binding and hydrolysis to act on non-native polypeptides thereby facilitating their folding, unfolding or disaggregation^1^. Some chaperones additionally play an important role in protein turnover by targeting proteins for degradation by proteolytic complexes^2^. As an example, the ClpX chaperone that is highly conserved between eubacteria and the mitochondria of eukaryotic cells, targets proteins for degradation by the separately encoded ClpP proteolytic complex^3,4^. Biochemical studies have given detailed insight into the mechanisms by which ClpX unfolds substrates and translocates unfolded substrates into the ClpP proteolytic chamber for degradation^5,6^. *In vitro* studies also showed that ClpX in the absence of ClpP is capable of unfolding model-substrates such as casein^7^, however, little is known of the contribution of the ClpP-independent chaperone activity of ClpX to cell physiology. Strikingly, mitochondrial ClpX is more widely conserved than mitochondrial ClpP, supporting that ClpX chaperone activity is of vital importance in mitochondrial cell biology^8^. Consistent with this notion, mitochondrial ClpX was recently shown to have a conserved role in the essential biosynthesis of heme by accelerating binding of a cofactor to a key enzyme^8^.

In bacteria, the importance of ClpX varies between species: ClpX is essential, or conditional essential, in diverse species such as *Streptomyces*, *Caulobacter, Synechococcus, Streptococcus, and Lactococcus*, while being dispensable for growth of *Escherichia coli* and in Salmonella^4,9–12^. Lethality of the *clpX* mutation has been traced to a critical role for ClpXP in cell cycle regulation in *Caulobacter*, but remains unexplained for most species^12^. Thus, it remains to be determined whether the essential function of ClpX is mediated via the ClpXP proteolytic complex, or, via the ClpP independent chaperone activity of ClpX. All identified substrates of bacterial ClpX are subject to degradation by ClpXP^13,14^. However, for two ClpXP substrates, the MuA transposase, and the plasmid replication factor TrfA, ClpX mediated unfolding—not ClpXP-mediated degradation—has proved to be the biologically important event^15–17^.

In the important pathogenic bacterium *Staphylococcus aureus*, ClpX is essential for virulence^10,18^, and consistent with this notion, ClpX is required for transcription of major *S. aureus* virulence genes, such as *hla* (alpha hemolysin) and *spa* (Protein A)^10,19,20^. *S. aureus* ClpX is, however, dispensable for growth under standard laboratory conditions^10^. Protein quality systems generally become essential in cells stressed by heat shock and other conditions that exacerbate the problem of protein misfolding and aggregation^1^. Surprisingly, inactivation of *clpX* in *S. aureus* improved survival at high temperatures, indicating that ClpX does not function as a classical heat shock chaperone in this organism^10^. Instead, *S. aureus* ClpX independently of ClpP, is required for growth at sub-optimal temperatures^21,22^. Interestingly, the cold-sensitive phenotype of the *S. aureus clpX* mutant seems to be caused by a yet uncharacterized role of the ClpX chaperone in *S. aureus* cell division^21,23^.

In order to get a more comprehensive picture of the ClpP-independent role of ClpX in *S. aureus* cell physiology, we here compared the global transcriptional changes induced by either a complete inactivation of ClpX, or by replacing ClpX with a ClpX_I265E_ variant. ClpX_I265E_ cannot interact with ClpP resulting in a strain that is devoid of ClpXP protease while retaining ClpP-independent ClpX chaperone activity^22,24^. The RNA sequencing (RNA-Seq) analysis confirms that ClpX has a profound impact on cell physiology and demonstrates that ClpX primarily impacts gene expression via ClpXP-dependent pathways. Independently of ClpXP, ClpX seems to impact transcription of very few genes with a predicted function in cell wall synthesis and cell division. Consistent with this finding, ClpX localizes in foci in close proximity to the division septa in dividing cells.

## Results and Discussion

### ClpX impacts *S. aureus* cell physiology primarily via ClpXP dependent pathways

RNA-Seq analysis was performed on RNA samples prepared from exponential cultures of the JE2 wild-type (a derivative of the multiple antibiotic resistant and community-acquired USA300 clone^25^), the JE2ΔclpX mutant, and JE2clpX_I265E_, expressing the ClpX_I265E_ variant that retains ClpX chaperone activity but is devoid of ClpXP protease activity due to a single amino acid substitution in the ClpP recognition IGF motif of ClpX^22,24^.

**Figure 1:**
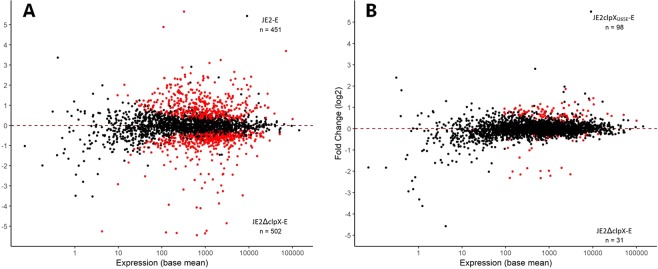
ClpX has a profound impact on *S. aureus* cell physiology that is mediated primarily via ClpXP dependent pathways. Global overview of gene expression in (**a**) JE2 wild-type versus JE2ΔclpX, and (**b**) JE2ΔclpX versus JE2clpX_I265E_ are depicted as MA plots visualizing the log2 fold change in expression between the two strains, as a function of the average expression of each gene. Each point represents a gene; red indicates an adjusted p-value < 0.01. The number of genes that are being significantly higher expressed in each of the two strains compared are stated in the top and bottom right corners of the plot: “E” following the strain names indicate that expression was compared in exponential cells.

The complete list of genes expressed differentially between the JE2 wild-type and the *clpX* deletion mutant can be found in Supplementary Table 1. In total, almost 1000 genes encoding proteins of diverse functions were found to be expressed significantly differentially (adjusted p-value < 0.01) between the JE2 wild-type and the *clpX* deletion mutant showing that ClpX has a profound impact on cell physiology (Fig. 1A, and Supplementary Table 1). Genes that change expression due to inactivation of the ClpXP protease were identified previously by comparing the transcriptome of the JE2 wild-type and the JE2clpX_I265E_ mutant (using the same datasets for the mid-exponential samples of JE2 and JE2clpX_I265E_), and will not be discussed here^22^. In the present study, we instead focus on genes that are differentially expressed between cells possessing ClpX chaperone (JE2clpX_I265E_), or not possessing ClpX chaperone activity (JE2ΔclpX), and therefore candidate to be subject to regulation via ClpX chaperone dependent pathways. Strikingly, relatively few genes were expressed significantly different between the JE2ΔclpX and the JE2clpX_I265E_ strains (Fig. 1B, and Supplementary Table 2), and while more than 100 genes were upregulated >2 fold in JE2ΔclpX cells compared to wild-type cells, only 12 genes were upregulated >2 fold in JE2ΔclpX compared to JE2clpX_I265E_ (Table 1 and Supplementary Table 2). Similarly, only 23 genes were down-regulated >2 fold in JE2ΔclpX cells compared to JE2clpX_I265E_, while 175 genes were down-regulated >2 fold in JE2ΔclpX compared to wild-type (Table 1 and Supplementary Table 2). Furthermore, the fold-changes of genes differently expressed between the JE2ΔclpX and the JE2clpX_I265E_ strains did not exceed 5 fold for any gene (Fig. 1b and Table 1). Taken together, these findings show that the transcriptional profiles of the JE2ΔclpX and the JE2clpX_I265E_ strains are very similar. Hence, inactivation of ClpXP seems to be causing most of the transcriptional changes observed between JE2ΔclpX and JE2 wild-type, indicating that ClpX impacts cell physiology mainly via its association to ClpP. Notably, ClpX seems to control expression of major virulence genes like *spa* (Protein A), *nuc* (nuclease), *geh* (lipase) and SAUSA300_1890 (staphopain A protease), or virulence regulators such as the *agr* quorum sensing system entirely via ClpXP-dependent pathways as transcription of these genes was reduced to the same extent in JE2ΔclpX and in JE2ClpX_*I265E*_ (Supplementary Table 1).

**Table 1:** Genes that are significantly differently transcribed in JE2ΔclpX relative to JE2 expressing ClpXI265E (1.5 fold cut-off).

Gene*	Predicted Function*	Fold change JE2ΔclpX/JE2X_I__265E_	Padj
SAUSA300_0807	SaPI gene, function unknown	5,0	4,6E-10
SAUSA300_0806	SaPI gene, function unknown	5,0	2,5E-09
SAUSA300_0805	SaPI excisionase	4,6	2,3E-05
SAUSA300_0808	SaPI gene, function unknown	4,5	5,8E-11
SAUSA300_0809	SaPI5 DNA primase	4,4	4,6E-10
SAUSA300_0811	SaPI gene, function unknown	4,0	1,8E-05
SAUSA300_0810	SaPI gene, function unknown	4,0	2,8E-11
SAUSA300_0804	putative transcriptional regulator	3,9	1,6E-10
SAUSA300_0813	SaPI gene, function unknown	3,6	1,7E-10
SAUSA300_0812	SaPI5 phage interference	3,6	1,1E-09
SAUSA300_0365	hypothetical protein SAS009 63aa	2,6	3,2E-04
SceD	SceD, WalkR controlled peptidoglycan hydrolases	2,0	5,7E-06
SAUSA300_1903	hypothetical protein, LexA operon	1,9	2,1E-03
cwrA	cwrA gene responding to cell wall damage	1,9	6,3E-03
**divIB**	**cell division protein**	**0,7**	**3,6E-08**
ureC	urease subunit alpha	0,7	9,2E-03
brnQ	branched-chain amino acid transport system II carrier protein	0,7	6,5E-03
SAUSA300_2523	hypothetical protein	0,7	8,5E-03
ureB	urease subunit beta	0,7	4,5E-03
asp23	alkaline shock protein 23	0,7	3,3E-03
SAUSA300_2455	putative fructose-1,6-bisphosphatase	0,7	1,1E-03
ureE	urease accessory protein UreE	0,7	6,5E-03
opuCd	glycine betaine/carnitine/choline transport system permease	0,7	6,0E-04
ureD	urease accessory protein UreD	0,7	5,5E-03
**murD**	**UDP-N-acetylmuramoyl-L-alanyl-D-glutamate synthetase**	**0,6**	**1,6E-23**
accC	acetyl-CoA carboxylase, biotin carboxylase	0,6	1,8E-03
dapD	tetrahydrodipicolinate acetyltransferase	0,6	1,7E-03
SAUSA300_1565	putative urea amidolyase	0,6	4,3E-03
SAUSA300_2524	hypothetical protein	0,6	9,4E-03
ptsG	phosphotransferase system, glucose-specific IIABC component	0,6	7,2E-03
rpmH	50 S ribosomal protein L34	0,6	5,7E-03
SAUSA300_0437	NLPA lipoprotein	0,6	3,0E-04
SAUSA300_2589	SasA adjesom cell wall anchor domain-containing protein	0,6	5,7E-09
thrC	threonine synthase	0,6	3,3E-04
thrB	homoserine kinase	0,6	2,1E-05
yfiA	ribosomal subunit interface protein	0,6	3,7E-03
SAUSA300_2548	hypothetical protein	0,6	4,8E-05
hom	homoserine dehydrogenase, hom	0,6	9,4E-04
SAUSA300_1739	hypothetical protein	0,6	8,0E-05
SAUSA300_1740	hypothetical protein, lipid anchored	0,6	1,1E-04
cap1C	capsular polysaccharide biosynthesis protein Cap1C	0,6	1,9E-03
clfA	clumping factor A	0,6	1,4E-03
glpT	glycerol-3-phosphate transporter	0,6	5,3E-04
ureA	urease subunit gamma	0,6	5,9E-03
**esxE**	**Esx E toxin-antitoxin system (type VII secretion locus)**	**0,6**	**4,6E-03**
SAUSA300_0271	ABC transporter ATP-binding protein	0,6	5,2E-03
SAUSA300_2289	hypothetical protein	0,6	4,5E-04
copZ	copper chaperone copZ	0,6	7,3E-05
**esxC**	**EsxC toxin (type VII secretion locus)**	**0,6**	**6,2E-03**
SAUSA300_0273	hypothetical protein	0,6	6,7E-08
nrdD	anaerobic ribonucleoside triphosphate reductase	0,6	4,7E-03
SAUSA300_0272	hypothetical protein	0,6	2,1E-03
lip	triacylglycerol lipase	0,6	5,7E-03
cap1B	capsular polysaccharide biosynthesis protein Cap1B	0,6	1,4E-05
SAUSA300_2538	amino acid permease family protein	0,6	8,3E-03
dapA	dihydrodipicolinate synthase	0,6	3,2E-04
spxA	transcriptional regulator Spx	0,6	4,9E-04
asd	aspartate semialdehyde dehydrogenase	0,6	2,0E-03
**SAUSA300_0277**	**peptidoglycan hydrolase linked to the type VII secretion locus**	**0,6**	**1,8E-04**
nrdG	anaerobic ribonucleotide reductase, small subunit	0,6	1,2E-03
SAUSA300_0558	putative proline/betaine transporter	0,6	1,6E-04
opp-3a	oligopeptide ABC transporter substrate-binding protein	0,6	5,1E-03
**pbpA**	**penicillin-binding protein 1**	**0,5**	**8,5E-37**
**SAUSA300_0769**	**hypothetical protein**	**0,5**	**6,7E-03**
SAUSA300_1286	aspartate kinase	0,5	7,9E-04
**esxB**	**EsxB toxin (type VII secretion locus)**	**0,5**	**2,8E-03**
SAUSA300_2614	hypothetical protein	0,5	5,2E-09
SAUSA300_2237	putative urea transporter	0,5	1,4E-03
**esxD**	**EsxD toxin (type VII secretion locus)**	**0,5**	**2,8E-03**
SAUSA300_0274	hypothetical protein	0,5	9,9E-05
**essC**	**EssC protein (membrane- bound, type VII secretion)**	**0,5**	**5,4E-04**
SAUSA300_2447	hypothetical protein	0,5	7,9E-04
**SAUSA300_0767**	**hypothetical protein**	**0,5**	**4,6E-03**
**essB**	**EssB protein (membrane- bound, type VII secretion)**	**0,5**	**5,2E-05**
sarZ	SarZ transcriptional regulator	0,5	3,6E-08
SAUSA300_0768	hypothetical protein	0,5	8,5E-04
**essA**	**EssA (membrane- bound, type VII secretion)**	**0,5**	**8,4E-04**
**ftsL**	**cell division protein**	**0,5**	**1,2E-15**
clpX	ATP-binding subunit ClpX	0,5	3,5E-46
cudT	BCCT family choline/carnitine/betaine transporter	0,4	1,4E-04
**esaA**	**EsaA membrane- bound, type VII secretion**	**0,4**	**6,1E-06**
**mraZ**	**cell division protein MraZ**	**0,4**	**1,0E-33**
**mraW**	**S-adenosyl-methyltransferase MraW**	**0,4**	**2,4E-24**
**esxA**	**EsxA toxin (type VII secretion locus)**	**0,4**	**1,8E-05**
betB	glycine betaine aldehyde dehydrogenase	0,3	8,5E-03

*Genes written in bold are predicted to be controlled by ClpX entirely via ClpXP-independent pathways.

### Expression of small RNAs (sRNAs) in JE2ΔclpX and JE2clpX_I265E_

Using a novel genome-wide annotation file for small RNAs (sRNAs) in *S. aureus*^26^, we furthermore performed a global analysis of how the ClpXP protease and ClpX chaperone impact expression of sRNAs. The list of sRNAs that are significantly differentially expressed between the JE2 wild-type and the *clpX* deletion mutant can be found in Table 2. In total 21 sRNA-genes were down-regulated in JE2ΔclpX (relative to JE2), while 17 genes were upregulated. In comparisons, only five sRNA genes were significantly differentially expressed between JE2ΔclpX and JE2clpX_I265E_ (depicted in bold in Table 2). Of these five sRNAs, only RsaOB seems to be regulated by ClpX entirely via its chaperone activity, as RsaOB transcription is similar in JE2 wild-type and JE2clpX_I265E_ cells (Supplementary Table 3). In contrast, ClpX seems to stimulate expression of the SAM riboswitch and T-box riboswitch via both ClpP-dependent and independent pathways, while a number of sRNAs seems to respond solely to inactivation of ClpXP, examples are the highly abundant Teg27, the Lysine riboswitch, SprC, and Ssr63 that all display 3–7 fold lower abundances in JE2ΔclpX and JE2clpX_I265E_, relative to JE2 wild-type. Thus, analyses of sRNA expression in JE2, JE2ΔclpX and JE2clpX_I265E_ support that ClpX impacts *S. aureus* cell physiology primarily via ClpXP dependent pathways.

**Table 2:** sRNAS that are significantly differently transcribed in JE2ΔclpX relative to the JE2 wild-type.

ID*^26^	sRNA*^26^	Fold change JE2/JE2ΔclpX	Padj
SAUSA300s013	Lysine riboswitch	**6,6**	1,42E-08
SAUSA300s084	Teg27	**5,8**	4,34E-26
SAUSA300s030	sprC	**4,4**	9,78E-03
**SAUSA300s003**	**T-box riboswitch**	**3,9**	**4,07E-16**
**SAUSA300s002**	**SAM riboswitch**	**3,7**	**1,46E-27**
SAUSA300s168	Sau-76	3,5	4,16E-06
SAUSA300s128	ssr63	3,4	3,24E-08
**SAUSA300s041**	**rsaOB**	**2,7**	**2,12E-04**
SAUSA300s176	Sau-26	2,5	2,67E-13
SAUSA300s281	tsr17	2,2	1,60E-03
SAUSA300s129	ssr68	2,2	2,38E-12
SAUSA300s284	tsr20	2,1	6,62E-04
SAUSA300s034	sprF3	1,9	3,74E-04
SAUSA300s285	tsr21	1,8	3,61E-04
SAUSA300s007	SAM riboswitch	1,8	8,49E-03
SAUSA300s181	Sau-72	1,8	1,07E-03
SAUSA300s166	Teg40as	1,7	9,04E-03
SAUSA300s065	Sau-59	1,6	9,90E-04
SAUSA300s279	tsr15	1,5	5,51E-03
SAUSA300s173	Sau-15	1,5	6,05E-03
SAUSA300s190	Sau-6515	1,3	2,02E-03
SAUSA300s191	Sau-6524	0,7	4,36E-03
SAUSA300s263	JKD6008sRNA403	0,7	2,02E-03
SAUSA300s187	Sau-6405	0,6	3,61E-04
SAUSA300s006	ssrA	0,6	2,56E-04
SAUSA300s238	JKD6008sRNA259	0,6	2,96E-04
SAUSA300s197	Sau-6769	0,6	3,86E-04
SAUSA300s180	Sau-39	0,6	7,41E-05
SAUSA300s165	Teg38as	0,6	9,78E-03
SAUSA300s182	Sau-6079	0,5	1,79E-03
SAUSA300s024	GlmS ribozyme	0,5	6,83E-07
**SAUSA300s140**	**ssr128**	**0,4**	**1,09E-03**
SAUSA300s080	rsaOV	0,4	3,77E-04
SAUSA300s164	Teg36as	0,3	8,58E-14
SAUSA300s211	JKD6008sRNA073	0,3	4,75E-03
SAUSA300s117	ssr8	0,3	9,09E-06
SAUSA300s152	Teg13	0,2	4,19E-06
**SAUSA300s226**	**JKD6008sRNA173**	**0,0**	**1,01E-11**

*Inactivation of ClpXP impacts expression of all listed sRNAs except rsaOB. Additionally, sRNAs written in bold are predicted to be controlled by ClpX also via ClpP independent pathways.

### Genes upregulated in JE2ΔclpX compared to JE2clpX_I265E_; de-repression of SaPI5 genes

**Figure 2:**
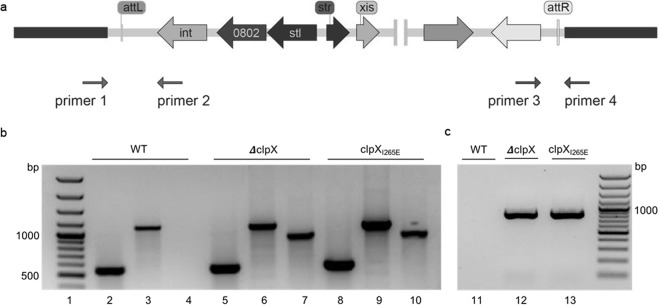
SaPI5 excises spontaneously from JE2ΔclpX and JE2clpX_I265E_. (**a**) Diagram depicting the annealing sites of primer pairs used to detect SaPI5 integrated into the chromosome (P1 + P2 and P3 + P4), and to detect excision of SaPI5 from the chromosome (P1 + P4), or excised, and circularized SaPI5 (P2 + P3). (**b**) PCR products amplified from DNA extracted from JE2 wild-type, JE2ΔclpX, and JE2clpX_I265E_ as indicated using primer sets P1 + P2 (lanes 2, 5 and 8), P3 + P4 (lanes 3, 6 and 9), and P2 + P3 (lanes 4, 7, and 10). (**c**) PCR fragments amplified using primer set P1 + P4 and DNA extracted from JE2 wild-type (lane 11), JE2ΔclpX, (lane 12), and JE2clpX_I265E_ (lane 13). DNA ladders are loaded in lane 1 and 14.

All highly up-regulated genes in JE2ΔclpX compared to JE2clpX_I265E_ localize in the phage-like pathogenicity island, SaPI5^27^, suggesting that ClpX independently of ClpP has a role in controlling the SaPI5 lifestyle (Table 1). Similar to pro-phages, SaPIs exhibit a typical modular organization with two divergent transcription units controlled by a phage-like genetic switch^28^. When SaPIs reside passively in the host chromosome, transcription of genes involved in the active life-style of the SaPI is prevented by the SaPI-encoded master repressor, Stl^28^. Consistent, with this general paradigm, the only actively transcribed SaPI5 genes in wild-type cells are genes involved in the passive SaPI life-style, namely the *int*-gene (Integrase), the *stl* gene (Stl repressor) and the upstream SAUSA300_0802 gene encoding a protein of unknown function (Supplemental Table 4). Interestingly, the SaPI5 genes that are highly upregulated in the *clpX* deletion mutant all localize in the transcription unit linked to the active lifestyle of the SaPI, and encode proteins with a predicted function in excision and replication of the SaPI element (Table 1). Importantly, SaPI genes were both significantly upregulated (5–10 fold) in the JE2ΔclpX mutant relative to the JE2clpX_I265E_ mutant and, as described before, between the JE2clpX_I265E_ mutant and the wild-type^22^ (Supplementary Table 4), supporting a dual role for ClpX in repression of SaPI5 involving both ClpXP and the ClpP-independent chaperone activity of ClpX. Accordingly, SaPI5 genes such as SAUSA300_0805 (predicted to encode the SaPI5 excisionase) are induced 30–45 fold in JE2ΔclpX relative to wild-type cells. The strong derepression of SaPI genes with a role in the active life-style of the SaPI, prompted us to set up PCR assays to assess whether SaPI5 excised from the chromosome can be detected in JE2ΔclpX mutant. Indeed, primer sets designed to detect the free, circular form of SaPI5 amplified a PCR-fragment of the expected size from cell extracts of JE2ΔclpX and JE2clpX_I265E_ but not from JE2 wild-type cells (Fig. 2). In contrast, a primer set designed to detect SaPI5 integrated into the chromosome amplified PCR products of the expected size from both the wild-type and *clpX* mutant cells (Fig. 2). De-repression of genes involved in excision and replication of the SaPI elements normally requires activation of specific helper phages^28^, and we therefore checked the RNA-seq data to see if genes in the JE2 prophages, ϕSA2usa and ϕSA3usa, change expression in the *clpX* mutants. Interestingly, this was not the case, hence, ClpX seems to impact transcription of SaPI genes independently of phage-induction, thereby revealing a novel layer of regulation of SaPI-elements.

### Genes down-regulated in JE2ΔclpX relative to JE2ClpX_I265E_

#### Genes predicted to be controlled by ClpX independently of ClpP

Genes that are down-regulated in the JE2ΔclpX compared to the JE2clpX_I265E_, but displaying similar expression in wild-type and the JE2clpX_I265E_ strain, candidate to be subject to ClpX regulation only via a ClpX chaperone dependent pathway. These genes map primarily in three genetic loci (Supplemental Table 2): the peptidoglycan synthesis operon (*mraZ-mraW-ftsL-pbpa*)^29^, the locus encoding the specialized type VII secretion system (T7SS) also referred to as the ESAT-6-like secretion systems (ESS) (SAUSA300_0278- SAUSA300_0289)^30^, and a predicted three-cistronic operon (SAUSA300_0767 - SAUSA300_0769) encoding three proteins of unknown function.

The finding that ClpX, but not ClpXP, impacts transcription of the cell wall synthesis operon *mraZ-mraW-ftsL-pbpa* operon is consistent with phenotypic characterization of the Δ*clpX* mutant, showing that the ClpX chaperone has an important role in *S. aureus* septum formation and cell division that is independent of ClpP^21,23^. In line with a ClpP independent role for the ClpX chaperone in cell wall synthesis, we note that also the *murD* gene (encoding the MurD D-glutamate ligase, a cytoplasmic peptidoglycan biosynthetic enzyme) was significantly down-regulated in cells lacking ClpX, while showing wild-type expression in cells only lacking ClpXP activity (Table 2). Furthermore, two genes encoding proteins with a predicted role in cell wall damage (*cwrA*)^31^, and cell wall hydrolysis (*sceD*)^32^, are among the most highly up-regulated genes in JE2Δ*clpX* mutant compared to the *clpX*_*I265E*_ mutant, Table 3.

The T7SS was initially discovered in pathogenic Mycobacteria, where it secretes proteins that are important for persistent infection of the mammalian host^33^. The *S. aureus* T7SS is only distantly related to the T7SSs found in mycobacteria, and mutational analysis has indicated that it contributes to virulence, intra-species competition, and potentially iron homeostasis^33^. The first five genes of the *ess* locus, *esxA-esaA-essA-esaB-essB*, are the most highly conserved and encode essential components of the secretion machinery^30,34^. Additionally, the *ess* locus encodes a number of secreted small effectors (EsxA-EsxD and EssD). Consistent with previous data, we find that *esxA* is by far the most highly expressed gene, however^34^, despite the differences in expression levels, the genes encoding the membrane-bound protein complex, and all *esx-*toxin genes are down-regulated 1.5–3 fold in cells lacking ClpX chaperone activity suggesting co-regulation of the *ess* locus by a pathway responding to ClpX chaperone deficiency. Moreover, the upstream and divergently transcribed gene, SAUSA300_0277 that was recently shown to encode a peptidoglycan hydrolase facilitating secretion of Ess effector molecules^35^, follows the same expression pattern, indicating that this gene is subject to regulation by the same ClpX chaperone dependent pathway. In support of a functional link between the ClpX chaperone and the T7SS, we have found that a *S. aureus clpX* mutant grown at 30 °C spontaneously acquired a single point mutation in *essC* introducing a T1153N substitution in EssC. However, as the suppressor strain additionally contained a loss-of function mutation in *ltaS*, we did not pursue to determine the phenotypes of the EssC_T1153N_ variant.

#### Genes subject to positive ClpX regulation via both ClpX chaperone and ClpXP-dependent pathways

A number of genes exhibited reduced transcription in JE2clpX_I265E_ relative to JE2 wild-type, as well as in JE2*Δ*clpX mutant relative to JE2clpX_I265E_, implying that these genes are subject to positive ClpX regulation via both ClpX chaperone and ClpXP-dependent pathways. Many of these genes are predicted to function in stress responses. Examples include *spxA* and *sarZ* encoding two transcriptional regulators that both use thiol-based redox switches to sense and respond to reactive oxygen species^36^; *yfiA* encoding the recently characterized ribosome hibernation factor^37^, and the *asp23*-SAUSA300_2143-SAUSA300_2144 operon that depends entirely on the alternative sigma factor, SigB for transcription. This finding suggests that ClpX contributes to control of SigB activity in *S. aureus* via both ClpP dependent pathways (as suggested in^38^) – and via ClpP independent pathways. Other genes regulated in this manner include genes encoding proteins with a predicted role in metabolism or transport of amino acid; examples are *lysC- asd - dapA - dapB - dapD* (encoding aspartate kinase, aspartate dehydregenase, dihydrodipicolinate synthase and reductase, and tetrahydrodipicolinate acetyltransferase, respectively), *hom - thrC - thrB* (encoding homoserine dehydrogenase, threonine synthase, and homoserine kinase, respectively), and opp-3a (oligopeptide ABC transporter substrate-binding protein), and brnQ1 (branched-chain amino acid transport system II carrier protein).

### Genes positively regulated by ClpXP and negatively by the ClpX chaperone

A number of genes up-regulated in JE2clpX_I265E_ relative to JE2 were down-regulated in JE2ΔclpX relative to JE2clpX_I265E_ indicating that ClpX and ClpXP affect transcription of these genes positively, and negatively, respectively (Supplemental Table 2). Examples include the highly up-regulated urease operon (*ureABCEFGHD*), and SAUSA300_2237 predicted to encode a urease transporter; genes predicted to encode proteins involved in transport and metabolism of the osmoprotectant, glycine betaine: *betB*, *bccT*, and, *opuCD*; as well as genes encoding proteins involved in copper transport: *copA* (copper-translocating P-type ATPase), and *copZ* (copper chaperone) encoded by the core genome and universally conserved in *S. aureus*^39^, as well as the horizontally acquired *copX* gene associated with the SCCmec element of USA300^40^. Finally, the RNAseq data suggested that *clpX* itself belongs to this group of genes. To follow up on this finding*, clpX* transcription was examined by Northern blot analysis. In agreement with previous data^10^, the *clpX* probe detected transcripts corresponding in size to *clpX* being transcribed both monocistronically as well as being co-transcribed with the upstream *tig* gene and the downstream *engB* gene (Supplemental Fig. 1). As expected, all *clpX* transcripts were reduced in size corresponding to the 651 bp deletion in the *clpX* deletion strain. For this reason, the apparent down-regulation of *clpX* in JE2ΔclpX may at least partly be ascribed to the reduced sizes of the *clpX* transcripts that will result in a reduced number of reads being mapped to *clpX* when the RNAseq analysis is performed on this strain.

### *S. aureus* ClpX localizes in single foci in close proximity to the division septum

**Figure 3:**
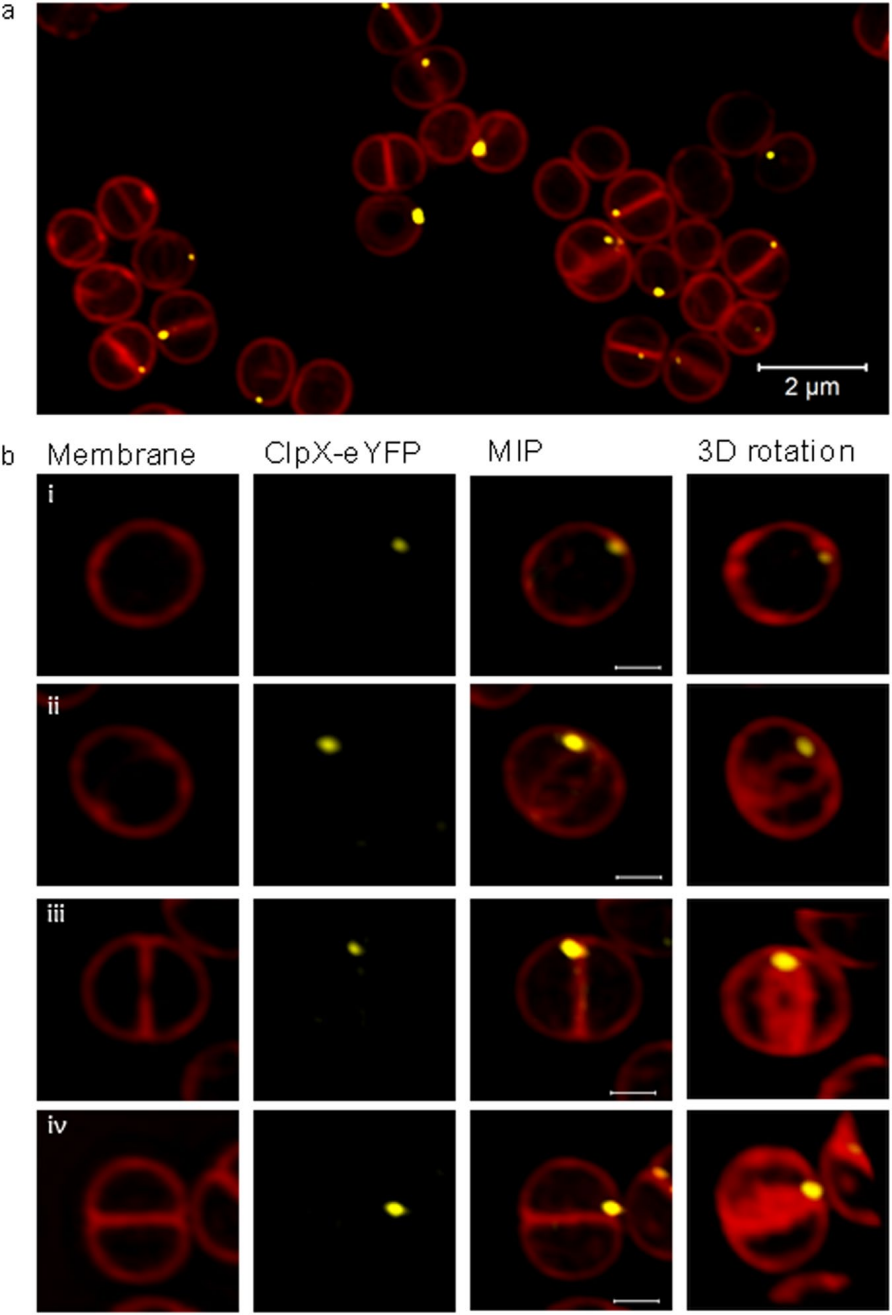
ClpX localizes in proximity to the division septum. ClpX localization in live *S. aureus* cells was analyzed by expressing an eYFP-tagged derivative of ClpX from an IPTG-inducible promoter in cells growing exponentially at 37 °C. Prior to SR-SIM imaging, cells were stained with the membrane dye Nile Red for 5 min. (**a**) Focal localization of ClpX-eYFP near the membrane of *S. aureus* cells (overview); scalebar, 2 μm. (**b**) ClpX-eYFP localization in *S. aureus* cells in different stages of cell division (i) non septating cells, (ii-iii) cells displaying an incomplete (non-closed) septum and (iv) cells displaying a closed septum. Left panels: membranes visualized using Nile-Red; middle panels to the left: ClpX-eYFP fluorescence; middle panels to the right and right panels; overlay of membrane and ClpX-eYFP fluorescence. The middle panels to the right show maximum intensity projections of the depicted cells, while the right panels show the 3D structures (transparency projection) of cells rotated around the vertical axis. Scale bars 0.5 μm.

Finally, we assessed the subcellular localization of an eYFP-tagged derivative of ClpX in live *S. aureus* cells using Super-Resolution Structured Illumination Microscopy (SR-SIM). The SR-SIM images revealed that ClpX localizes in single foci near the membrane in *S. aureus* wild-type cells (Fig. 3). To the best of our knowledge, localization of ClpX has not previously been addressed in cocci, however, in the rod shaped bacteria *Bacillus subtili*s^41,42^, *Mycobacterium tuberculosis*^43^, and *Caulobacter crescentus*^44,45^ ClpX similarly localizes in single foci that in the majority of cells are located near the cell poles, however, transient localization at the septal position was observed for *B. subtilis*^41,42^ and *Caulobacter* ClpX^44^. In comparisons, *S. aureus* ClpX localizes in close proximity to the ingrowing septum in the vast majority of wild-type cells in the process of septation (86 + 3% of septating cells displaying an eYFP signal). Strikingly, the ClpX-eYFP signal seems to be present in only one of two daughter cells (Fig. 3bii–iv) and consistent with this finding only 51 ± 3% of all *S. aureus* cells displayed a ClpX-eYFP signal. Previous data have indicated that ClpX contributes to two processes of importance to *S. aureus* cell division, namely septum synthesis and stability of cell wall hydrolases involved in daughter cell separation^23^. The interesting finding that ClpX localizes close to the division septum is consistent with ClpX being functionally linked to *S. aureus* cell division.

## Concluding Remarks

We here demonstrate that ClpX has a profound impact on *S. aureus* cell physiology that is mediated primarily via ClpXP-dependent pathways. Based on the finding that ClpX impacts expression of virulence genes entirely via ClpXP-dependent pathways, we speculate that the essential role of ClpX in *S. aureus* virulence is linked to the activity of the ClpXP protease. Strikingly, the most strongly up-regulated genes in the *clpX* mutants are involved in the excision and replication of the phage-like pathogenicity island, SaPI5, and accordingly circular SaPI elements excised from the chromosome were detected in the *S. aureus clpX* mutants. ClpX seems to control expression of SaPI5 and a large number of other genes via pathways involving both ClpXP and ClpX chaperone activity supporting that the ClpX chaperone activity in combination with ClpXP proteolytic activity modulate a large subgroup of ClpX substrates. Finally, ClpX seems to impact transcription of very few genes entirely via ClpP-independent pathways. These genes map primarily in two genetic loci: the peptidoglycan synthesis operon (*mraZ-mraW-ftsL-pbpa*), and the genetic locus encoding the protein secretion components and effector molecules of T7SS. Of particular interest, we demonstrate that ClpX localizes in single foci in close proximity to the division septum. In *Caulobacter* proper subcellular positioning of ClpXP at the cell pole is important for the role of ClpXP in cell cycle regulation^46^. The finding that *S. aureus* ClpX localizes in distinct foci close to the septum in dividing *S. aureus* cells supports that ClpX activity is confined to a distinct subcellular site, and lends support to the idea that ClpX functions in *S. aureus* cell division.

## Methods

### Bacterial strains and growth conditions

The bacterial strains used in this study are described in^10,22^. For liquid cultures *S. aureus* strains were grown in tryptic soy broth media (TSB; Oxoid); for solid medium, 1.5% agar was added to make TSA plates. Erythromycin (7.5 µg ml^−1^) was added as required. In all experiments, bacterial strains freshly streaked from the frozen stocks on TSA plates, with antibiotics added as required and incubated overnight at 37 °C, were used to inoculate 20 ml of TSB medium in 200-ml flasks to allow efficient aeration of the medium. The starting OD was always below 0.05 and unless otherwise stated, strains were grown with vigorous agitation at 200 rpm at 37 °C. The growth was followed by measuring the optical densities at 600 nm.

### RNA extraction, library preparation and RNA sequencing

The RNA extraction was performed as described previously^10^. Briefly, cultures inoculated to a starting OD_600_ below 0.02 were grown at 37 °C with vigorous shaking, and when the cultures reached OD_600_ = 0.7 +/− 0.1 (exponential samples) samples were withdrawn for the isolation of RNA. Cells were quickly cooled on an EtOH/dry ice bath and frozen at −80 °C until extraction of RNA. RNA was isolated from three biological replicates grown on different days: cells were lyzed mechanically using the FastPrep machine (MP Biomedicals) and RNA was isolated by the RNeasy mini kit (Quiagen, Valencia, Calif) according to the manufacturer’s instructions. RNA integrity was confirmed using a TapeStation with RNA HS screen tapes (Agilent). rRNA was removed by the Ribo pure kit (Illumina, Little Chesterford, USA). High quality RNA was delivered to DNASense ApS (Denmark) for transcriptomic analysis. To remove ribosomal RNA the Ribo-Zero kit for Bacteria (Illumina, Little Chesterford, USA) was used. Based on TapeStation gels (Agilent), the majority of ribosomal RNA was removed in all 9 samples. Transcriptome libraries were prepared using the stranded TruSeq mRNAseq protocol, which enables strand specific identification of transcripts. Library preparation and subsequent Illumina HiSeq sequencing (1 × 50 bp was successful for all samples. The sequencing generated on average 11 million reads per sample, and of these, an average of 8 million reads mapped to non-rRNA transcripts. Note that the RNA seq analysis described here was performed at the same time, and uses the same datasets for the JE2 wild-type and JE2clpX_I265E_ ^22^.

### Analysis of gene expression, bioinformatic processing and analysis

Raw sequence reads in fastq format were trimmed using USEARCH (v10.0.2132) -fastq_filter with the settings -fastq_minlen 45 -fastq_truncqual 20. The trimmed transcriptome reads were mapped to the predicted protein coding genes or sRNAs in the annotated genome of USA300^26^ using USEARCH (v10.0.2132) -usearch_global with the following settings -id 0.98 -otutabout -strand plus (the genes and sRNAs were reverse complemented to allow for strand specific mapping in USEARCH). For each mapping, the number of reads mapping to a specific gene was calculated using a simple command line script: grep “ˆ@”-v map.sam | cut -f3 | sort | uniq -c > result.txt. The DESeq2^47^ workflow was applied to normalize the read counts and identify differentially expressed genes. Counts from rRNA genes were removed prior to the analysis as these would have been heavily influened by the Ribo-Zero rRNA removal step. Principal Component Analysis (PCA) of the normalized gene expression data from DESeq. 2 showed that the samples clustered strain specifically.

### SaPI5 PCR assay

Primer pairs were designed to detect SaPI5 integrated into the chromosome: P1 (5′-GCGTAAAAATTGAAGATGCTAAG) + P2 (5′-CTCACGTTACTGAACAGATGG) and P3 (5′ GCACTTGCTATCAATGTTTTTGTT’) + P4 (5′-CCTTTTGCTTGTTCAAATTTACTT), as depicted in Fig. 2. The unoccupied chromosomal attachment was detected by combining P1 + P4, while the excised circular SaPI was detected by using P2 + P3 in Fig. 2. Template DNA was extracted by re-suspending a streak of freshly grown colonies into 50 μL of 0.9% NaCl and boiling at 98 °C for 5 minutes. 1 μL of cell lysate was used as template in PCR-reactions using DreamTaq Green PCR Mastermix (2X) from Thermo Fisher Scientific and the settings: initial denaturation at 95 °C for 3 minutes; 35X cycles of denaturing at 95 °C for 30 seconds, annealing at 50 °C for 30 seconds, and elongation at 72 °C for 1 minute, and followed by a final extension at 72 °C for 3 minutes. The PCR products were analyzed on a 1.2% agarose gel at 125 V for 20 minutes and visualized with ChemiDoc™ XRS + System (Bio-Rad, CA).

### Northern blotting

Bacterial strains were inoculated as described above, and grown at 37 °C until the cultures reached OD_600_ = 0.7 +/− 0.1(T = 0). At this point, the cultures were subsequently transferred to a water bath at 28 °C. At T = 0, T = 10 min and T = 30 min following the temperature shift, 1 ml samples were withdrawn for the isolation of RNA. The RNA extraction was performed as described above and Northern blotting was performed as described previously^20^. Briefly, 5 µg of RNA from each preparation was loaded onto a 1% agarose gel and separated in 10 mM sodium phosphate buffer as described previously. RNA was transferred to a positively charged nylon membrane by capillary blotting. The hybridization was performed using a [^32^P] labeled *clpX*-specific probe, amplified with clpX-f: 5′-GCTGTGGCTGTTTATAACCAC and clpX-r: 5′-GTGCTGTAATAACTACCTTCG primers, and labeled with [^32^P]dCTP (Perkin-Elmer) using the Ready-to-Go DNA-labeling beads from GE-healthcare.

### ClpX localization by Super-Resolution structured illumination microscopy (SR-SIM)

#### Construction of plasmid expressing eYFP-tagged ClpX from an inducible promoter

The *clpX* gene was amplified wit primers ClpX_PCQ11f (5′-CCGTGGCTAGCATGTTTAAATTCAATGAAGA) and ClpX_PCQ11r (5′-CTGCCATGGATAATTCAGCTGATGTTTTACT) (NheI and NcoI restriction sites are underlined). The PCR product was subsequently digested with NheI and NcoI and cloned into pCQ11 plasmid^48^ digested with the same two restriction enzymes and treated with 1 U FastAP Thermosensitive Alkaline Phosphatase to avoid relegation. Following transformation into *Escherichia coli* (IMB08) colonies harboring the desired *clpX-eYFP* fusion were detected with colony-PCR using the primers ClpX_PCQ11f and eYFPr (5′-TCACCTGAAACTGAGAATTTATGACC), and the purified plasmid was subsequently introduced into two different *S. aureus* wild-type model strains (8325-4 and SA564) using the protocol described in^49,49^. The relative amounts of un-tagged ClpX and ClpX-eYFP in cells grown in the presence of increasing concentrations of IPTG (0, 10, 40, 70, 100 µM) were determined by Western blot analysis (Supplementary Fig. 2) using antibodies specific for *S. aureus* ClpX as described by Jelsbak *et al*.^20^.

#### SR-SIM

Prior to imaging *S. aureus*/pCQ11clpX::eYFP was inoculated into 20 mL TSB to an initial OD_600_ of 0.02. Cultures were grown at 37 °C with aeration for 3 generations before adding 10 µM IPTG and cells were subsequently growing exponentially until OD_600_ = 0.5 +/− 0.1. At this time 1 mL samples were collected and Nile Red was added to a final concentration of 5 mg/ml and the samples were incubated for 5 min at 37 °C. Images was acquired with an Elyra PS.1 microscope (Zeiss) using a Plan Apochromat 63x/1.4 oil DIC M27 objective and a Pco.edge 5.5 camera. Images of cell stained with NileRed were acquired using a 561 nm laser (200 mW) with five grid rotations and a grating period of 34 mm. Laser power was set to 7% with an exposure time of 50 ms. eYFP signal was acquired using a 488 nm laser (200 mW) with five grid rotations and a grating period of 34 mm. Laser power was set to 7% with an exposure time of 50 ms. 40 images with a distance of 0.11 µm were obtained to construct a Z-stack and 3D rotations. Images were reconstructed using ZEN software (black edition, 2012, version 8.1.0.484) based on a structured illumination algorithm, using synthetic, channel specific optical transfer functions and noise filter settings ranging from 6 to 8. Z-stacks were visualized by 3D structures (transparency projection) rotated around the vertical axis and by maximum intensity projection (MIP) using a subset of the z-stacks (15 slides). Quantitative analysis was performed on 100 cells in each of two biological replicates.

## Supplementary information

Supplementary Info File #1

## Data Availability

The dataset generated using RNA-sequencing is provided in Supplementary Tables 1 and 2.
