# Supplementary material for: Staphylococcus aureus ClpX localizes at the division septum and impacts transcription of genes involved in cell division, T7-secretion, and SaPI5-excision
Source: Sci Rep. 2019 Nov 11;9:16456. doi: 10.1038/s41598-019-52823-0 (PMC6848492; doi:10.1038/s41598-019-52823-0)
Supplement: Supplementary file 1 — Supplementary Info File #1 [file 41598_2019_52823_MOESM1_ESM.pdf]

Staphylococcus aureus ClpX localizes at the division septum and impacts transcription of genes involved in cell division, T7-secretion, and SaPI5-excision

Camilla Jensen[1], Marie J. Fosberg[1,] Ida Thalsø-Madsen[1], Kristoffer T. Bæk[1], and Dorte Frees[1]*

Supplementary Figure 1

(Northern blot showing that the deletion in *clpX* does not prevent transcription of clpX, but reduces the sizes of all *clpX* mRNAs consistent with the deletion in *clpX*)

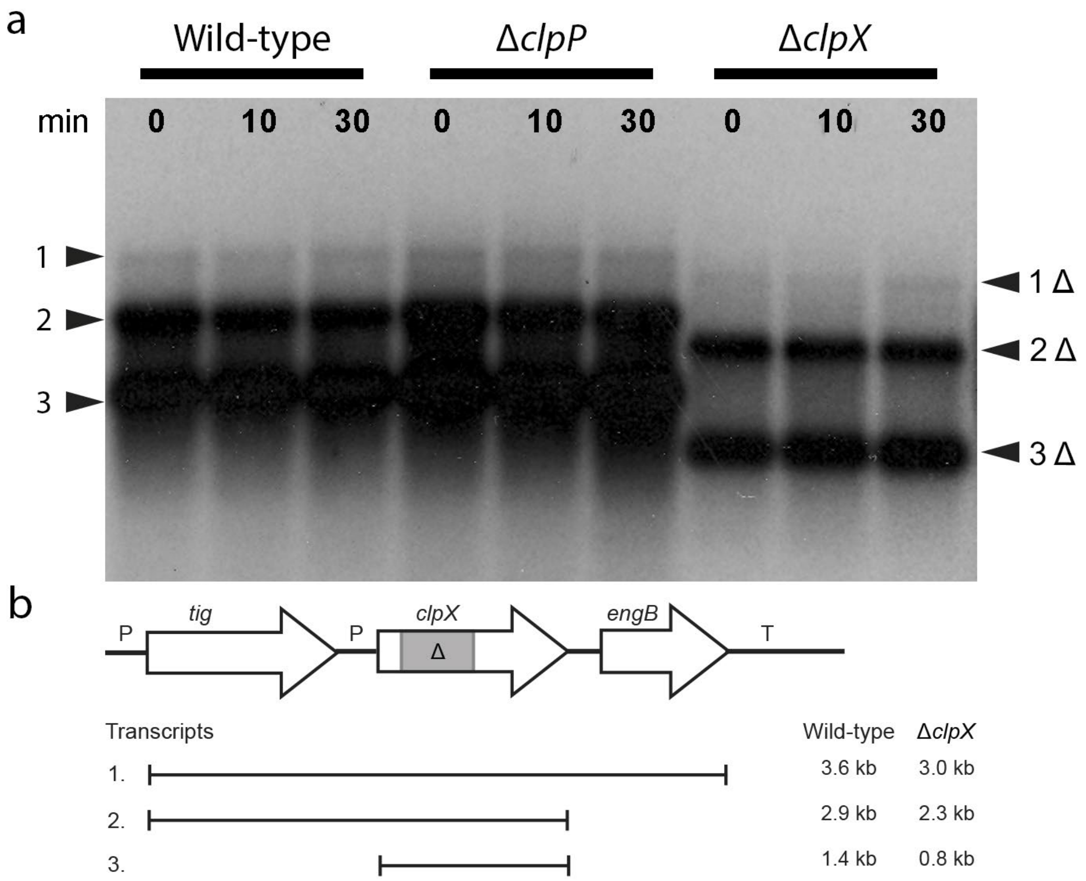

Supplemental Figure 1. Northern blotting showing reduced sizes of *clpX* mRNAs in the *clpX* deletion strain. (a) RNA was isolated from *S. aureus* wild type cells and the ΔclpP[46] and ΔclpX[46] derived here from growing either exponentially at 37°C (T=0) or 10, or 30 min after shifting cells to 28°C as indicated. *clpX* mRNAs were detected using a [^{32}P] labeled *clpX*-specific DNA probe. In agreement with previous data[47], the *clpX* probe detected transcripts corresponding in size to *clpX* being monocistronically transcribed (transcript 3) as well as being co-transcribed with the upstream *tig* gene and the downstream *engB* gene (transcripts 1 and 2). The sizes of the transcripts were estimated from the positions of the 16S and 23S rRNA. The chromosomal organization of the *clpX* locus, and putative mapping of the detected transcripts are illustrated schematically in (b).The 651 bp deletion in *clpX* is depicted in grey.

Supplementary Figure 2

(Western blot showing the relative amount of ClpX and ClpX-eYFP when grown in the presence of increasing IPTG concentrations)

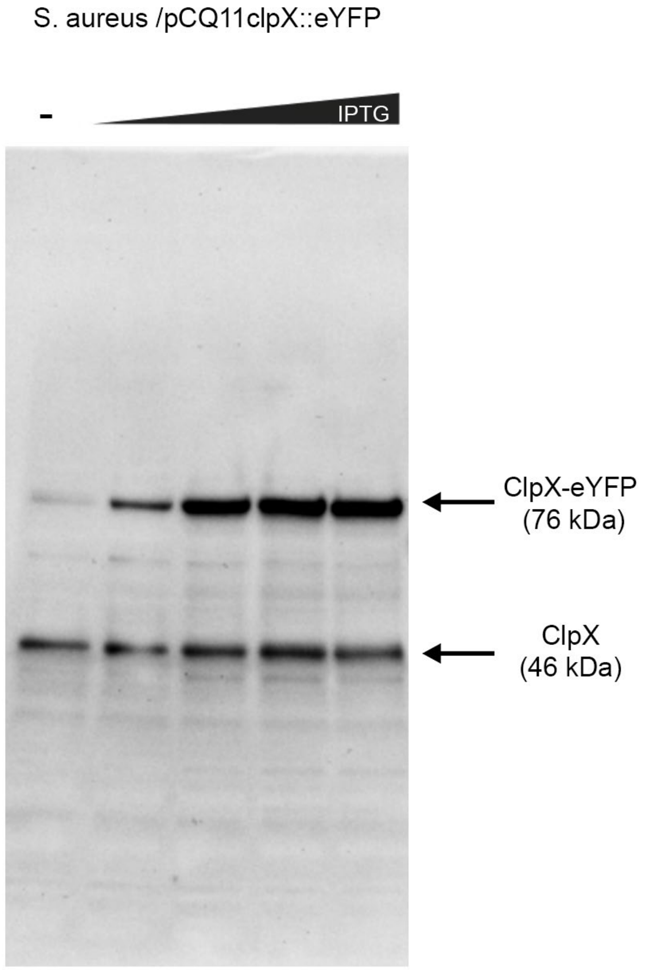

Supplemental Figure 2. Western blot showing the relative amount of ClpX and ClpX-eYFP when grown in the presence of increasing IPTG concentrations. *S. aureus* wild type cells expressing a translational fusion of *clpX* to *eYFP* from the P*spac* promoter were grown with increasing IPTG concentrations 0, 10, 40, 70, 100 µM, and the relative amount of native ClpX and ClpX-eYFP were determined by Western blotting using antibodies directed against ClpX.

Supplementary Table 1. The complete list of genes expressed significantly differentially between the JE2 wild-type and JE2ΔclpX.

ID	Fold change wt/ΔclpX	Fold change ΔclpX/wt	padj	Significant	JE2 E1	JE2 E2	JE2 E3	ΔclpX E1	ΔclpX E2	ΔclpX E3	$clpX_{I265E}$ E1	$clpX_{I265E}$ E2	$clpX_{I265E}$ E3	Gene	Product
SAUSA300_0807	0,0	43,6	2,9E-44	Yes	34	17	15	1414	694	707	154	207	201	SAUSA300_0807	SaPI5 gene unknown function
SAUSA300_0804	0,0	41,0	1,6E-60	Yes	44	16	26	1502	1025	966	239	365	284	SAUSA300_0804	SaPI5-gene predicted to encode the Str transcriptional regulator
SAUSA300_0808	0,0	40,6	8,4E-37	Yes	19	8	3	493	262	361	61	93	96	SAUSA300_0808	SaPI5 gene unknown function
SAUSA300_0806	0,0	39,7	3,9E-25	Yes	9	3	6	357	153	187	34	52	53	SAUSA300_0806	SaPI5 gene unknown function
SAUSA300_0805	0,0	37,5	3,7E-29	Yes	70	23	34	2361	1181	1175	273	432	310	SAUSA300_0805	SaPI5 gene predicted to encode an excisionase
SAUSA300_0809	0,0	28,9	3,3E-52	Yes	262	138	149	8170	3810	3861	996	1369	1216	SAUSA300_0809	SaPI5 gene predicted to encode a DNA primase
SAUSA300_2238	0,1	17,3	1,1E-56	Yes	30	27	29	595	442	466	838	823	787	ureA	urease subunit gamma
SAUSA300_0810	0,1	16,8	1,3E-43	Yes	68	59	55	1560	803	705	225	302	246	SAUSA300_0810	SaPI5 gene unknown function
SAUSA300_0811	0,1	15,4	4,9E-18	Yes	27	14	12	423	163	204	51	58	86	SAUSA300_0811	SaPI5 gene unknown function
SAUSA300_2239	0,1	14,6	2,8E-95	Yes	94	93	95	1818	1199	1117	2028	2162	2058	ureB	urease subunit beta
SAUSA300_2240	0,1	11,1	7,0E-85	Yes	421	428	467	6229	4159	4263	7070	7683	7302	ureC	urease subunit alpha
SAUSA300_0812	0,1	10,1	7,8E-33	Yes	364	248	254	4372	2018	2339	687	891	859	SAUSA300_0812	hypothetical protein
SAUSA300_2241	0,1	9,6	4,6E-72	Yes	178	193	197	2358	1546	1570	2638	2792	2866	ureE	urease accessory protein UreE
SAUSA300_0813	0,1	9,0	4,1E-29	Yes	66	64	82	974	433	522	155	197	180	SAUSA300_0813	SaPI5 gene unknown function
SAUSA300_1093	0,1	8,7	9,9E-19	Yes	48	76	55	635	340	584	403	247	508	pyrB	aspartate carbamoyltransferase catalytic subunit
SAUSA300_2242	0,1	7,9	7,9E-48	Yes	241	224	202	2376	1352	1521	2426	2430	2777	ureF	urease accessory protein UreF
SAUSA300_0431	0,1	7,5	1,6E-03	Yes	3	3	0	16	23	6	13	18	11	SAUSA300_0431	hypothetical protein
SAUSA300_1092	0,1	7,1	2,5E-16	Yes	84	95	83	696	458	715	565	336	581	pyrP	uracil permease
SAUSA300_2237	0,2	6,5	5,8E-26	Yes	96	68	84	659	395	555	950	980	1146	SAUSA300_2237	putative urea transporter
SAUSA300_1094	0,2	6,4	8,0E-20	Yes	98	160	129	1003	604	886	741	518	787	pyrC	dihydroorotase
SAUSA300_1692	0,2	5,7	7,2E-04	Yes	17	16	17	22	22	242	27	20	24	SAUSA300_1692	hypothetical protein
SAUSA300_1096	0,2	5,5	3,8E-25	Yes	376	606	466	3258	1975	2680	2218	1744	2218	carB	carbamoyl phosphate synthase large subunit
SAUSA300_2244	0,2	5,4	1,0E-42	Yes	378	334	362	2320	1538	1932	2683	2858	3320	ureD	urease accessory protein UreD
SAUSA300_1095	0,2	5,4	1,3E-19	Yes	120	151	132	897	515	752	709	499	594	carA	carbamoyl phosphate synthase small subunit
SAUSA300_1097	0,2	5,3	2,8E-21	Yes	57	113	108	586	371	544	420	367	450	pyrF	orotidine 5'-phosphate decarboxylase
SAUSA300_2243	0,2	5,3	3,4E-49	Yes	342	371	353	2402	1626	1650	2642	2797	2849	ureG	urease accessory protein UreG
SAUSA300_0365	0,2	4,5	6,7E-11	Yes	106	48	58	378	273	283	108	157	100	SAUSA300_0365	hypothetical protein
SAUSA300_2441	0,2	4,4	7,2E-16	Yes	1177	1081	1209	5942	5368	3875	3317	5213	3162	fnbA	fibronectin binding protein A
SAUSA300_2051	0,2	4,1	2,5E-23	Yes	133	160	116	587	621	468	265	297	267	SAUSA300_2051	hypothetical protein
SAUSA300_2493	0,3	3,9	3,3E-11	Yes	46	25	32	128	116	156	70	86	57	SAUSA300_2493	hypothetical protein
SAUSA300_0017	0,3	3,9	9,3E-32	Yes	357	545	382	1516	1986	1490	1610	1372	1412	purA	adenylosuccinate synthetase
SAUSA300_1098	0,3	3,8	9,4E-17	Yes	121	138	101	574	324	489	339	297	366	pyrE	orotate phosphoribosyltransferase
SAUSA300_1641	0,3	3,7	1,3E-28	Yes	178	268	233	891	910	731	752	753	692	gltA	citrate synthase
SAUSA300_1981	0,3	3,6	7,2E-04	Yes	6	7	8	25	21	28	21	19	16	SAUSA300_1981	phage terminase family protein
SAUSA300_0953	0,3	3,5	8,0E-06	Yes	86	49	81	205	130	414	185	132	231	SAUSA300_0953	hypothetical protein
SAUSA300_0987	0,3	3,3	2,3E-05	Yes	290	152	391	1017	641	1113	629	883	880	SAUSA300_0987	cytochrome D ubiquinol oxidase, subunit II
SAUSA300_0433	0,3	3,3	8,8E-29	Yes	478	546	491	1866	1756	1408	1424	1573	1754	cysM	cysteine synthase/cystathionine beta-synthase
SAUSA300_0270	0,3	3,2	2,9E-15	Yes	1060	1144	1011	3535	4044	2856	2628	2966	2409	lytM	peptidoglycan hydrolase
SAUSA300_0224	0,3	3,2	1,6E-12	Yes	492	307	312	1287	1263	1024	625	881	616	coa	staphylocoagulase
SAUSA300_1192	0,3	3,2	3,1E-17	Yes	171	259	260	766	807	632	573	568	443	glpK	glycerol kinase
SAUSA300_2319	0,3	3,0	3,0E-10	Yes	71	126	161	386	377	333	294	316	346	SAUSA300_2319	pyridine nucleotide-disulfide oxidoreductase

SAUSA300_2318	0,3	3,0	1,7E-10	Yes	54	73	89	191	270	194	212	226	206	SAUSA300_2318	acetyltransferase
SAUSA300_0434	0,3	2,9	1,2E-23	Yes	1666	1336	1240	4551	3880	4067	4164	4260	4494	metB	cystathionine gamma-synthase
SAUSA300_2523	0,3	2,9	7,4E-16	Yes	118	140	133	368	451	334	572	594	573	SAUSA300_2523	hypothetical protein
SAUSA300_1640	0,3	2,9	3,8E-64	Yes	686	718	701	2099	2055	1932	1978	2078	1896	icd	isocitrate dehydrogenase
SAUSA300_2249	0,3	2,9	2,4E-18	Yes	8200	9237	6212	24376	22614	21235	19081	19934	20655	ssaA	secretory antigen precursor SsaA
SAUSA300_2160	0,4	2,8	1,1E-09	Yes	54	42	43	143	129	116	101	81	88	SAUSA300_2160	MerR family transcriptional regulator
SAUSA300_0398	0,4	2,8	2,9E-03	Yes	9	7	11	22	30	21	20	18	17	SAUSA300_0398	superantigen-like protein
SAUSA300_0986	0,4	2,7	2,9E-05	Yes	933	590	1010	2807	1925	2179	1977	2640	2121	SAUSA300_0986	cytochrome D ubiquinol oxidase, subunit I
SAUSA300_2159	0,4	2,7	1,5E-24	Yes	679	438	511	1472	1494	1369	1333	1375	1343	SAUSA300_2159	aldo/keto reductase family protein
SAUSA300_2253	0,4	2,7	2,1E-11	Yes	3012	3453	2341	8336	8688	6396	5535	5921	7066	ssaA	secretory antigen precursor SsaA
SAUSA300_0394	0,4	2,6	6,0E-12	Yes	87	74	78	264	191	167	165	174	172	SAUSA300_0394	FAD/NAD(P)-binding Rossmann fold superfamily protein
SAUSA300_2206	0,4	2,5	9,5E-03	Yes	31	39	24	47	34	156	48	44	38	SAUSA300_2206	hypothetical protein
SAUSA300_0741	0,4	2,5	1,0E-16	Yes	1141	1395	1255	4205	2765	2602	2103	2220	2145	uvrB	excinuclease ABC subunit B
SAUSA300_2585	0,4	2,5	1,3E-11	Yes	171	217	292	705	511	511	631	555	738	SAUSA300_2585	hypothetical protein
SAUSA300_1539	0,4	2,5	2,8E-09	Yes	378	530	677	1563	1306	1064	1065	1090	1253	dnaJ	chaperone protein DnaJ
SAUSA300_0300	0,4	2,5	3,9E-03	Yes	4	24	33	59	61	38	65	47	57	SAUSA300_0300	hypothetical protein
SAUSA300_2522	0,4	2,5	1,8E-04	Yes	36	23	36	82	71	79	93	95	126	SAUSA300_2522	hypothetical protein
SAUSA300_2162	0,4	2,5	3,8E-12	Yes	248	342	353	740	896	683	523	539	547	SAUSA300_2162	M23/M37 peptidase domain-containing protein
SAUSA300_2475	0,4	2,4	1,4E-09	Yes	221	329	202	625	752	449	497	474	445	SAUSA300_2475	hypothetical protein
SAUSA300_0419	0,4	2,4	2,6E-08	Yes	130	237	209	469	473	445	440	463	522	SAUSA300_0419	tandem lipoprotein
SAUSA300_2263	0,4	2,3	9,2E-03	Yes	63	51	142	157	119	323	202	181	335	SAUSA300_2263	putative transposase
SAUSA300_1903	0,4	2,3	2,8E-06	Yes	245	194	225	651	408	453	251	280	266	SAUSA300_1903	hypothetical protein
SAUSA300_2148	0,4	2,3	8,2E-12	Yes	204	271	358	721	657	531	466	486	461	SAUSA300_2148	hypothetical protein
SAUSA300_2524	0,4	2,3	2,2E-07	Yes	61	46	59	108	144	121	183	192	205	SAUSA300_2524	hypothetical protein
SAUSA300_0207	0,4	2,2	1,1E-08	Yes	88	92	76	183	209	179	177	174	159	SAUSA300_0207	hypothetical protein
SAUSA300_0413	0,4	2,2	7,8E-03	Yes	7	11	11	22	23	22	18	22	22	SAUSA300_0413	tandem lipoprotein
SAUSA300_0742	0,4	2,2	1,1E-12	Yes	2496	2733	2669	7854	4880	4933	3873	4258	3927	uvrA	excinuclease ABC subunit A
SAUSA300_2393	0,4	2,2	1,5E-08	Yes	921	1191	873	2208	2444	2008	3013	2280	2959	opuCa	glycine betaine/carnitine/choline ABC transporter ATP-binding protein
SAUSA300_2387	0,4	2,2	2,6E-20	Yes	545	635	549	1301	1350	1204	1153	1171	1040	SAUSA300_2387	NAD dependent epimerase/dehydratase family protein
SAUSA300_1538	0,5	2,2	8,9E-17	Yes	697	828	999	2124	1757	1734	1595	1670	1703	prmA	ribosomal protein L11 methyltransferase
SAUSA300_1747	0,5	2,2	1,1E-04	Yes	16	34	55	77	85	77	77	72	83	SAUSA300_1747	hypothetical protein
SAUSA300_2474	0,5	2,2	3,8E-09	Yes	185	264	174	486	520	349	324	353	336	SAUSA300_2474	hypothetical protein
SAUSA300_0170	0,5	2,2	4,8E-08	Yes	124	127	126	287	245	281	245	265	239	SAUSA300_0170	aldehyde dehydrogenase
SAUSA300_0411	0,5	2,1	5,6E-03	Yes	13	8	16	30	26	24	37	39	35	SAUSA300_0411	tandem lipoprotein
SAUSA300_0407	0,5	2,1	9,9E-03	Yes	70	18	39	109	61	100	72	73	56	SAUSA300_0407	superantigen-like protein
SAUSA300_2104	0,5	2,1	3,0E-08	Yes	9441	8291	7649	18612	17776	17751	18104	18883	17208	glmS	glucosamine--fructose-6-phosphate aminotransferase
SAUSA300_0079	0,5	2,1	3,2E-19	Yes	968	908	820	1995	1900	1833	2977	2576	2891	SAUSA300_0079	putative lipoprotein
SAUSA300_2473	0,5	2,1	2,0E-11	Yes	1372	1909	1457	3320	3795	2912	2994	2707	2783	SAUSA300_2473	hypothetical protein
SAUSA300_0799	0,5	2,1	8,8E-10	Yes	339	287	247	788	513	544	443	461	385	int	integrase
SAUSA300_0397	0,5	2,1	8,4E-03	Yes	17	15	8	29	29	24	25	31	27	SAUSA300_0397	superantigen-like protein
SAUSA300_2515	0,5	2,1	4,9E-05	Yes	43	32	50	93	75	97	81	55	64	SAUSA300_2515	TetR family transcriptional regulator
SAUSA300_2163	0,5	2,1	3,3E-07	Yes	118	166	230	316	421	352	259	266	295	SAUSA300_2163	hypothetical protein
SAUSA300_2262	0,5	2,1	3,6E-03	Yes	550	350	791	819	837	1858	931	935	1216	SAUSA300_2262	hypothetical protein
SAUSA300_0231	0,5	2,1	1,8E-05	Yes	432	287	413	996	581	775	943	971	1173	SAUSA300_0231	ABC transporter substrate-binding protein

SAUSA300_2470	0,5	2,1	2,3E-05	Yes	57	59	74	127	125	144	142	141	164	sdaAB	L-serine dehydratase, iron-sulfur-dependent, beta subunit
SAUSA300_2317	0,5	2,1	4,3E-15	Yes	1326	1324	1300	2692	3149	2348	2891	3058	2448	SAUSA300_2317	putative zinc-binding dehydrogenase
SAUSA300_2492	0,5	2,1	7,0E-10	Yes	220	293	234	505	599	442	553	518	539	SAUSA300_2492	acetyltransferase family protein
SAUSA300_1876	0,5	2,1	7,1E-20	Yes	241	236	213	467	475	473	409	379	386	SAUSA300_1876	DNA polymerase IV
SAUSA300_2392	0,5	2,1	7,7E-10	Yes	519	567	490	1045	1133	1064	1583	1160	1440	opuCb	glycine betaine/carnitine/choline ABC transporter
SAUSA300_1297	0,5	2,0	1,5E-09	Yes	190	265	253	431	544	481	450	443	394	SAUSA300_1297	acylphosphatase
SAUSA300_1099	0,5	2,0	4,1E-04	Yes	68	81	91	138	112	244	139	102	155	SAUSA300_1099	hypothetical protein
SAUSA300_0967	0,5	2,0	2,2E-03	Yes	489	1479	677	1923	2090	1380	1261	1155	1584	purK	phosphoribosylaminoimidazole carboxylase ATPase subunit
SAUSA300_1492	0,5	2,0	3,1E-03	Yes	292	236	397	492	469	916	449	387	534	SAUSA300_1492	hypothetical protein
SAUSA300_2089	0,5	2,0	1,3E-16	Yes	415	484	527	1086	889	926	853	967	851	pdp	pyrimidine-nucleoside phosphorylase
SAUSA300_1276	0,5	2,0	2,8E-07	Yes	87	78	82	154	158	186	165	148	216	opp-2B	oligopeptide permease, channel-forming protein
SAUSA300_0078	0,5	2,0	3,8E-19	Yes	3294	3301	2688	6651	5995	6057	9391	8240	8972	copA	ATPase copper transport
SAUSA300_0156	0,5	2,0	2,5E-04	Yes	44	33	41	95	79	65	77	79	78	cap5E	capsular polysaccharide biosynthesis protein Cap5E
SAUSA300_2348	0,5	2,0	4,1E-08	Yes	83	143	114	224	253	209	212	210	200	SAUSA300_2348	hypothetical protein
SAUSA300_2090	0,5	2,0	4,2E-08	Yes	96	133	135	261	241	231	250	271	234	deoC	deoxyribose-phosphate aldolase
SAUSA300_1595	0,5	2,0	3,0E-11	Yes	1567	2039	2403	3662	4314	3979	3764	3975	3625	tgt	queuine tRNA-ribosyltransferase
SAUSA300_1658	0,5	2,0	2,1E-09	Yes	1152	1165	1061	2478	2060	2166	2147	1789	1972	SAUSA300_1658	hypothetical protein
SAUSA300_2390	0,5	2,0	1,0E-10	Yes	693	571	592	1139	1198	1328	2082	1568	1953	opuCd	glycine betaine/carnitine/choline transport system permease
SAUSA300_2586	0,5	2,0	3,4E-12	Yes	508	653	673	1424	1135	1082	1622	1508	1597	SAUSA300_2586	hypothetical protein
SAUSA300_0968	0,5	2,0	2,8E-03	Yes	116	319	195	429	479	340	389	248	383	purC	phosphoribosylaminoimidazole-succinocarboxamide synthase
SAUSA300_2440	0,5	2,0	1,9E-04	Yes	1446	1014	1646	3209	2422	2399	2806	4045	2591	fnbB	fibronectin binding protein B
SAUSA300_1674	0,5	2,0	1,2E-10	Yes	3236	4043	3293	6768	7622	6227	5079	5004	5071	SAUSA300_1674	putative serine protease HtrA
SAUSA300_2391	0,5	1,9	9,2E-07	Yes	1214	1231	961	2374	2020	2206	3347	2312	3012	opuCc	glycine betaine/carnitine/choline ABC transporter
SAUSA300_0223	0,5	1,9	8,1E-06	Yes	108	84	71	144	169	182	133	123	114	SAUSA300_0223	hypothetical protein
SAUSA300_0686	0,5	1,9	2,7E-20	Yes	722	895	763	1592	1596	1374	1316	1322	1300	nagA	N-acetylglucosamine-6-phosphate deacetylase
SAUSA300_1088	0,5	1,9	1,8E-12	Yes	302	331	302	551	594	642	516	507	488	SAUSA300_1088	glyoxalase family protein
SAUSA300_0140	0,5	1,9	1,8E-08	Yes	655	906	942	1715	1581	1495	1488	1634	1434	deoC	deoxyribose-phosphate aldolase
SAUSA300_2053	0,5	1,9	1,0E-09	Yes	207	184	273	392	447	431	431	419	359	SAUSA300_2053	hypothetical protein
SAUSA300_0154	0,5	1,9	2,2E-03	Yes	36	26	21	51	52	52	86	69	103	cap5C	capsular polysaccharide biosynthesis protein Cap5C
SAUSA300_2513	0,5	1,9	6,4E-06	Yes	51	113	86	178	156	152	136	122	129	SAUSA300_2513	hypothetical protein
SAUSA300_2485	0,5	1,9	4,2E-07	Yes	125	209	159	344	339	262	296	293	280	SAUSA300_2485	methylated DNA-protein cysteine methyltransferase
SAUSA300_2236	0,5	1,9	6,7E-09	Yes	406	514	441	868	962	743	613	717	733	SAUSA300_2236	hypothetical protein
SAUSA300_2631	0,5	1,9	1,8E-03	Yes	157	151	129	247	288	287	331	253	268	SAUSA300_2631	putative N-acetyltransferase
SAUSA300_0325	0,5	1,9	8,9E-06	Yes	70	58	83	148	131	123	115	119	113	SAUSA300_0325	glycine cleavage H-protein
SAUSA300_0031	0,5	1,9	9,2E-05	Yes	211	168	185	283	350	424	369	344	359	SAUSA300_0031	hypothetical protein
SAUSA300_0408	0,5	1,9	3,9E-04	Yes	348	181	165	465	360	469	405	417	338	SAUSA300_0408	hypothetical protein
SAUSA300_0628	0,5	1,9	2,1E-12	Yes	250	355	346	614	638	542	668	745	653	SAUSA300_0628	glycerol-3-phosphate cytidylyltransferase
SAUSA300_0096	0,5	1,9	1,0E-04	Yes	50	55	63	95	123	98	104	104	104	SAUSA300_0096	hypothetical protein
SAUSA300_1298	0,5	1,9	3,5E-03	Yes	292	300	500	594	509	944	473	484	638	SAUSA300_1298	hypothetical protein
SAUSA300_2125	0,5	1,9	3,0E-10	Yes	2024	2612	2049	4113	4680	3713	3879	3925	3188	SAUSA300_2125	ATP-binding Mrp/Nbp35 family protein
SAUSA300_1177	0,5	1,9	6,7E-14	Yes	458	586	519	954	1034	936	774	687	774	cinA	competence/damage-inducible protein cinA
SAUSA300_1734	0,5	1,9	1,4E-04	Yes	51	105	100	190	167	131	119	117	129	SAUSA300_1734	hypothetical protein
SAUSA300_1275	0,5	1,9	1,9E-04	Yes	78	58	64	120	105	147	131	98	146	SAUSA300_1275	peptide ABC transporter permease
SAUSA300_2222	0,5	1,9	2,8E-06	Yes	218	380	340	644	650	459	572	476	471	moaE	molybdopterin converting factor, subunit 2

SAUSA300_2103	0,5	1,9	1,8E-07	Yes	97	122	121	243	205	190	218	213	213	SAUSA300_2103	ABC transporter ATP-binding protein
SAUSA300_1138	0,5	1,9	1,1E-04	Yes	558	1087	1159	1932	1825	1458	1456	1671	1690	sucC	succinyl-CoA synthetase subunit beta
SAUSA300_1607	0,5	1,9	2,5E-04	Yes	367	244	299	423	616	643	561	522	543	SAUSA300_1607	hypothetical protein
SAUSA300_2647	0,5	1,9	6,1E-04	Yes	68	59	70	129	100	138	127	108	129	rnpA	ribonuclease P
SAUSA300_0490	0,5	1,9	7,3E-05	Yes	702	956	1154	2094	1633	1490	1373	1366	1517	hslO	Hsp33-like chaperonin
SAUSA300_1925	0,5	1,9	4,7E-04	Yes	76	72	55	125	110	137	114	121	118	SAUSA300_1925	phiPVL ORF17-like protein
SAUSA300_0355	0,5	1,8	7,3E-26	Yes	1466	1527	1469	2745	2776	2730	2605	2668	2418	SAUSA300_0355	acetyl-CoA acetyltransferase
SAUSA300_2084	0,5	1,8	2,8E-14	Yes	414	446	519	871	860	824	905	989	903	coaA	pantothenate kinase
SAUSA300_0363	0,5	1,8	3,8E-04	Yes	73	93	139	183	184	200	150	148	174	SAUSA300_0363	hypothetical protein
SAUSA300_2446	0,5	1,8	6,4E-09	Yes	429	407	519	801	817	879	787	899	874	SAUSA300_2446	hypothetical protein
SAUSA300_1378	0,5	1,8	1,7E-03	Yes	39	33	39	81	64	59	97	64	68	SAUSA300_1378	hypothetical protein
SAUSA300_0328	0,5	1,8	1,2E-09	Yes	138	180	170	335	290	278	251	269	272	SAUSA300_0328	lipoate-protein ligase A family protein
SAUSA300_2484	0,5	1,8	8,1E-32	Yes	4857	4556	4132	8320	8317	8192	7789	8013	7057	SAUSA300_2484	hydroxymethylglutaryl-CoA synthase
SAUSA300_2081	0,5	1,8	2,7E-26	Yes	3284	3288	3259	6099	6105	5784	6067	6081	5880	pyrG	CTP synthetase
SAUSA300_2560	0,5	1,8	2,9E-06	Yes	220	183	195	313	387	390	440	512	383	SAUSA300_2560	hypothetical protein
SAUSA300_2469	0,5	1,8	3,6E-07	Yes	188	208	224	403	355	380	432	495	451	sdaAA	L-serine dehydratase, iron-sulfur-dependent, alpha subunit
SAUSA300_2514	0,5	1,8	4,9E-03	Yes	24	37	23	59	49	45	27	29	37	SAUSA300_2514	hypothetical protein
SAUSA300_2588	0,5	1,8	1,7E-06	Yes	224	211	238	390	348	488	475	512	574	secY	preprotein translocase subunit SecY
SAUSA300_0638	0,6	1,8	3,9E-04	Yes	41	58	31	81	82	74	88	94	73	SAUSA300_0638	phosphotransferase mannnose-specific family component IIA
SAUSA300_1039	0,6	1,8	8,7E-14	Yes	369	412	381	740	731	635	495	490	461	rnhC	ribonuclease HIII
SAUSA300_0085	0,6	1,8	1,3E-03	Yes	44	35	57	92	74	84	76	80	73	SAUSA300_0085	hypothetical protein
SAUSA300_1875	0,6	1,8	9,5E-05	Yes	498	394	395	815	702	800	590	541	665	SAUSA300_1875	exonuclease
SAUSA300_2495	0,6	1,8	8,1E-07	Yes	501	364	385	635	801	805	1375	1259	1165	SAUSA300_2495	copper chaperone copZ
SAUSA300_2462	0,6	1,8	2,9E-06	Yes	1363	1216	912	1980	2199	2095	2267	2035	2036	frp	NAD(P)H-flavin oxidoreductase
SAUSA300_1667	0,6	1,8	1,6E-05	Yes	133	200	190	281	368	294	244	236	256	SAUSA300_1667	putative glycerophosphoryl diester phosphodiesterase
SAUSA300_1440	0,6	1,8	2,6E-04	Yes	56	57	55	124	94	85	171	135	154	SAUSA300_1440	hypothetical protein
SAUSA300_0381	0,6	1,8	5,1E-13	Yes	1265	1234	1027	2102	2227	1976	2083	1978	1822	SAUSA300_0381	putative NAD(P)H-flavin oxidoreductase
SAUSA300_2404	0,6	1,8	4,6E-09	Yes	416	570	496	886	1026	745	813	820	771	SAUSA300_2404	hypothetical protein
SAUSA300_2630	0,6	1,8	3,4E-08	Yes	995	826	720	1692	1365	1482	2044	1894	1794	nixA	high-affinity nickel-transporter
SAUSA300_1710	0,6	1,8	4,9E-11	Yes	1004	1049	1143	1740	2259	1719	1706	1785	1606	SAUSA300_1710	putative lysophospholipase
SAUSA300_1339	0,6	1,8	6,8E-03	Yes	565	435	696	766	874	1391	764	723	1011	SAUSA300_1339	hypothetical protein
SAUSA300_0660	0,6	1,8	4,1E-03	Yes	200	135	274	334	269	484	276	265	335	SAUSA300_0660	hypothetical protein
SAUSA300_2512	0,6	1,8	3,8E-05	Yes	78	78	84	161	156	115	127	121	136	SAUSA300_2512	glyoxalase family protein
SAUSA300_2312	0,6	1,8	1,1E-07	Yes	552	861	791	1333	1365	1227	1281	1483	1215	mqo	malate:quinone oxidoreductase
SAUSA300_0327	0,6	1,8	2,8E-07	Yes	181	211	196	410	348	290	303	315	313	SAUSA300_0327	hypothetical protein
SAUSA300_1128	0,6	1,8	6,4E-04	Yes	376	685	839	1356	1104	916	936	916	981	ftsY	signal recognition particle-docking protein FtsY
SAUSA300_0417	0,6	1,8	7,6E-03	Yes	17	31	31	41	50	51	60	46	50	SAUSA300_0417	tandem lipoprotein
SAUSA300_2530	0,6	1,8	8,3E-09	Yes	150	144	151	253	274	258	288	302	298	SAUSA300_2530	TetR family transcriptional regulator
SAUSA300_2083	0,6	1,8	4,3E-12	Yes	307	295	370	558	566	588	561	555	572	SAUSA300_2083	acetyltransferase
SAUSA300_1606	0,6	1,8	8,6E-04	Yes	163	101	171	240	228	293	187	179	215	SAUSA300_1606	hypothetical protein
SAUSA300_1296	0,6	1,7	2,1E-05	Yes	245	190	201	266	410	429	409	334	358	SAUSA300_1296	hypothetical protein
SAUSA300_0289	0,6	1,7	6,3E-04	Yes	297	275	480	559	504	779	666	647	790	SAUSA300_0289	hypothetical protein
SAUSA300_1383	0,6	1,7	6,5E-03	Yes	37	34	26	62	55	53	63	59	64	SAUSA300_1383	phiSLT ORF484-like protein, lysin
SAUSA300_1020	0,6	1,7	6,6E-13	Yes	1309	1470	1153	2159	2528	2144	2127	2254	1974	SAUSA300_1020	glycerophosphoryl diester phosphodiesterase family protein

SAUSA300_2078	0,6	1,7	2,1E-16	Yes	1915	2254	2121	3905	3611	3379	3519	3339	3488	murA	UDP-N-acetylglucosamine 1-carboxyvinyltransferase
SAUSA300_2381	0,6	1,7	4,9E-07	Yes	954	871	670	1308	1741	1255	1345	1297	1128	SAUSA300_2381	hypothetical protein
SAUSA300_1348	0,6	1,7	7,0E-16	Yes	649	779	701	1237	1222	1214	1037	1055	1119	SAUSA300_1348	tRNA CCA-pyrophosphorylase
SAUSA300_0362	0,6	1,7	2,5E-05	Yes	164	284	267	435	416	389	332	341	323	SAUSA300_0362	hypothetical protein
SAUSA300_1847	0,6	1,7	4,3E-14	Yes	938	836	922	1705	1545	1391	1326	1357	1237	SAUSA300_1847	hypothetical protein
SAUSA300_0298	0,6	1,7	7,0E-03	Yes	34	33	46	70	65	62	71	74	100	SAUSA300_0298	hypothetical protein
SAUSA300_1446	0,6	1,7	5,6E-08	Yes	127	117	111	209	187	209	195	177	201	SAUSA300_1446	hypothetical protein
SAUSA300_0737	0,6	1,7	3,9E-29	Yes	9521	9197	9783	16648	16168	15911	16217	16783	16548	secA	preprotein translocase subunit SecA
SAUSA300_0840	0,6	1,7	1,5E-05	Yes	83	94	113	145	177	175	181	176	147	SAUSA300_0840	hypothetical protein
SAUSA300_0418	0,6	1,7	3,0E-03	Yes	39	73	63	110	111	80	113	92	106	SAUSA300_0418	tandem lipoprotein
SAUSA300_1369	0,6	1,7	1,6E-08	Yes	549	580	571	949	998	945	851	739	806	SAUSA300_1369	pyridine nucleotide-disulfide oxidoreductase
SAUSA300_0903	0,6	1,7	8,3E-07	Yes	1435	1146	1320	2171	2054	2383	1748	1862	1978	SAUSA300_0903	hypothetical protein
SAUSA300_2445	0,6	1,7	5,9E-18	Yes	901	836	881	1437	1475	1518	1535	1596	1741	SAUSA300_2445	MerR family transcriptional regulator
SAUSA300_1983	0,6	1,7	9,7E-04	Yes	458	802	437	1056	905	912	943	869	758	groES	co-chaperonin GroES
SAUSA300_0101	0,6	1,7	1,2E-03	Yes	70	58	60	109	112	96	144	132	172	SAUSA300_0101	tandem lipoprotein
SAUSA300_2558	0,6	1,7	1,0E-11	Yes	608	542	561	924	943	1020	1039	1106	1050	nsaS	nisin susceptibility-associated sensor histidine kinase
SAUSA300_2494	0,6	1,7	5,2E-09	Yes	2299	2477	1754	3728	3704	3577	5217	4629	4884	SAUSA300_2494	copper-translocating P-type ATPase
SAUSA300_0443	0,6	1,7	3,0E-12	Yes	1202	1145	1161	1972	2005	1933	1923	1968	1871	SAUSA300_0443	hypothetical protein
SAUSA300_0421	0,6	1,7	5,8E-04	Yes	434	453	383	708	637	790	957	782	833	SAUSA300_0421	hypothetical protein
SAUSA300_2223	0,6	1,7	1,0E-04	Yes	147	222	256	389	320	351	268	252	310	mobB	molybdopterin-guanine dinucleotide biosynthesis protein B
SAUSA300_0489	0,6	1,7	1,4E-16	Yes	19109	20280	21091	36174	32554	33002	32849	34537	33396	SAUSA300_0489	putative cell division protein FtsH
SAUSA300_2394	0,6	1,7	2,3E-08	Yes	475	418	507	749	783	817	907	957	843	SAUSA300_2394	hypothetical protein
SAUSA300_2210	0,6	1,7	2,0E-08	Yes	2017	2528	2584	4353	4135	3477	3440	3704	2923	glcU	glucose uptake protein
SAUSA300_0930	0,6	1,7	2,6E-05	Yes	934	961	827	1473	1650	1436	1492	1356	1305	SAUSA300_0930	lipoate-protein ligase A family protein
SAUSA300_0965	0,6	1,7	1,9E-06	Yes	1747	2471	1739	2883	3786	3307	4149	4214	3616	folD	bifunctional 5,10-methylene-tetrahydrofolate dehydrogenase/ 5,10-methylene-tetrahydrofolate cyclohydrolase
SAUSA300_0988	0,6	1,7	8,1E-05	Yes	478	614	740	1111	1054	910	913	1093	928	trkA	potassium uptake protein
SAUSA300_1299	0,6	1,7	8,7E-11	Yes	1629	1638	1768	2773	2865	2795	2110	2270	2483	SAUSA300_1299	putative tellurite resistance protein
SAUSA300_0141	0,6	1,7	1,5E-07	Yes	1727	2343	2349	3933	3639	3182	3447	3792	3330	deoB	phosphopentomutase
SAUSA300_1785	0,6	1,7	4,7E-04	Yes	981	927	1243	1417	1407	2445	1656	1523	1747	SAUSA300_1785	putative ABC transporter protein EcsB
SAUSA300_1188	0,6	1,7	1,4E-16	Yes	1614	1558	1539	2746	2384	2741	2550	2380	2641	mutS	DNA mismatch repair protein MutS
SAUSA300_1139	0,6	1,7	1,8E-05	Yes	1199	1760	1657	2823	2614	2278	2452	2636	2466	sucD	succinyl-CoA synthetase subunit alpha
SAUSA300_2529	0,6	1,7	1,1E-04	Yes	1843	1142	1410	2152	2504	2668	3668	3473	2701	SAUSA300_2529	hypothetical protein
SAUSA300_2587	0,6	1,7	1,2E-12	Yes	906	991	950	1735	1567	1452	2031	1996	2232	SAUSA300_2587	accessory secretory protein Asp1
SAUSA300_0420	0,6	1,7	1,6E-04	Yes	762	764	765	1423	1130	1260	1583	1336	1573	SAUSA300_0420	hypothetical protein
SAUSA300_0442	0,6	1,7	1,1E-05	Yes	1110	959	1054	1594	1758	1829	1641	1740	1662	SAUSA300_0442	hypothetical protein
SAUSA300_2643	0,6	1,7	1,1E-06	Yes	1614	2080	1501	2979	3236	2412	2876	2918	2561	SAUSA300_2643	ParB family chromosome partioning protein ParB family
SAUSA300_0453	0,6	1,7	2,6E-03	Yes	836	1325	1621	2262	2291	1715	1978	1926	1757	SAUSA300_0453	hypothetical protein
SAUSA300_1071	0,6	1,7	2,2E-07	Yes	926	1089	1046	1832	1732	1505	1465	1331	1441	SAUSA300_1071	hypothetical protein
SAUSA300_2129	0,6	1,7	3,7E-04	Yes	378	364	599	866	619	740	624	680	698	SAUSA300_2129	putative hemolysin III
SAUSA300_2221	0,6	1,7	2,3E-03	Yes	64	93	115	164	154	138	121	114	108	moaD	molybdopterin converting factor, subunit 1
SAUSA300_0828	0,6	1,7	1,9E-16	Yes	1075	1149	1052	1915	1808	1689	1590	1620	1539	SAUSA300_0828	5'-nucleotidase family protein
SAUSA300_2147	0,6	1,6	2,6E-08	Yes	1741	1844	1410	2893	2905	2435	2578	2392	2208	SAUSA300_2147	alcohol dehydrogenase, zinc-containing
SAUSA300_1350	0,6	1,6	4,0E-09	Yes	198	193	214	325	348	325	329	304	329	SAUSA300_1350	hypothetical protein
SAUSA300_2646	0,6	1,6	5,0E-09	Yes	655	633	610	1206	957	962	1089	1080	1116	trmE	tRNA modification GTPase TrmE

**SAUSA300_0684**	**0,6**	**1,6**	**1,6E-03**	**Yes**	**492**	**697**	**869**	**1267**	**1141**	**978**	**1124**	**1566**	**1159**	**fruB**	**fructose 1-phosphate kinase**
**SAUSA300_0158**	**0,6**	**1,6**	**2,6E-03**	**Yes**	**41**	**59**	**41**	**85**	**71**	**78**	**102**	**95**	**98**	**cap5G**	**capsular polysaccharide biosynthesis protein Cap5G**
**SAUSA300_2421**	**0,6**	**1,6**	**1,2E-04**	**Yes**	**183**	**172**	**212**	**328**	**287**	**317**	**271**	**198**	**287**	**SAUSA300_2421**	**hypothetical protein**
**SAUSA300_2102**	**0,6**	**1,6**	**2,3E-09**	**Yes**	**627**	**676**	**604**	**994**	**1099**	**1031**	**1011**	**1076**	**989**	**SAUSA300_2102**	**haloacid dehalogenase-like hydrolase**
**SAUSA300_2645**	**0,6**	**1,6**	**2,1E-11**	**Yes**	**1242**	**1528**	**1382**	**2571**	**2159**	**2085**	**2204**	**2199**	**2201**	**gidA**	**tRNA uridine 5-carboxymethylaminomethyl modification enzyme GidA**
**SAUSA300_2584**	**0,6**	**1,6**	**1,4E-04**	**Yes**	**613**	**827**	**1058**	**1610**	**1276**	**1218**	**1567**	**1459**	**1746**	**SAUSA300_2584**	**preprotein translocase subunit SecA**
**SAUSA300_2491**	**0,6**	**1,6**	**2,0E-07**	**Yes**	**519**	**601**	**499**	**863**	**923**	**864**	**1040**	**1041**	**912**	**SAUSA300_2491**	**1-pyrroline-5-carboxylate dehydrogenase**
**SAUSA300_0324**	**0,6**	**1,6**	**1,2E-04**	**Yes**	**134**	**136**	**144**	**229**	**225**	**225**	**195**	**201**	**190**	**SAUSA300_0324**	**hypothetical protein**
**SAUSA300_0157**	**0,6**	**1,6**	**6,0E-03**	**Yes**	**56**	**45**	**43**	**84**	**68**	**80**	**102**	**86**	**99**	**cap5F**	**capsular polysaccharide biosynthesis protein Cap5F**
**SAUSA300_0931**	**0,6**	**1,6**	**2,5E-03**	**Yes**	**599**	**439**	**636**	**825**	**793**	**1107**	**1106**	**1029**	**891**	**SAUSA300_0931**	**hypothetical protein**
**SAUSA300_0627**	**0,6**	**1,6**	**4,6E-10**	**Yes**	**653**	**788**	**826**	**1311**	**1164**	**1228**	**1176**	**1326**	**1246**	**tagX**	**teichoic acid biosynthesis protein X**
**SAUSA300_2294**	**0,6**	**1,6**	**7,6E-07**	**Yes**	**324**	**301**	**302**	**476**	**497**	**532**	**452**	**479**	**515**	**SAUSA300_2294**	**hypothetical protein**
**SAUSA300_0237**	**0,6**	**1,6**	**1,3E-06**	**Yes**	**422**	**445**	**359**	**660**	**699**	**630**	**563**	**565**	**608**	**SAUSA300_0237**	**inosine-uridine preferring nucleoside hydrolase**
**SAUSA300_0491**	**0,6**	**1,6**	**5,5E-04**	**Yes**	**7738**	**11442**	**7672**	**13094**	**18120**	**12342**	**11425**	**10032**	**9415**	**cysK**	**cysteine synthase A**
**SAUSA300_0868**	**0,6**	**1,6**	**5,7E-10**	**Yes**	**1157**	**916**	**1038**	**1582**	**1690**	**1747**	**1558**	**1425**	**1474**	**spsB**	**signal peptidase IB**
**SAUSA300_1885**	**0,6**	**1,6**	**2,5E-16**	**Yes**	**2571**	**2906**	**2788**	**4813**	**4516**	**4032**	**4076**	**3808**	**3617**	**ligA**	**DNA ligase**
**SAUSA300_2561**	**0,6**	**1,6**	**3,3E-03**	**Yes**	**57**	**84**	**78**	**123**	**116**	**118**	**123**	**145**	**109**	**phoB**	**alkaline phosphatase**
**SAUSA300_1130**	**0,6**	**1,6**	**8,7E-11**	**Yes**	**2397**	**2830**	**2969**	**4575**	**4528**	**4120**	**4029**	**4047**	**4196**	**ffh**	**signal recognition particle protein**
**SAUSA300_2224**	**0,6**	**1,6**	**2,7E-04**	**Yes**	**429**	**713**	**627**	**1072**	**990**	**796**	**790**	**705**	**728**	**moeA**	**molybdopterin biosynthesis protein A**
**SAUSA300_2200**	**0,6**	**1,6**	**1,8E-03**	**Yes**	**2975**	**3640**	**3927**	**6421**	**5391**	**5156**	**6181**	**6715**	**5533**	**rpsS**	**30S ribosomal protein S19**
**SAUSA300_0992**	**0,6**	**1,6**	**1,5E-04**	**Yes**	**2236**	**1730**	**2130**	**3013**	**3190**	**3542**	**3446**	**2666**	**2998**	**SAUSA300_0992**	**hypothetical protein**
**SAUSA300_0248**	**0,6**	**1,6**	**2,3E-07**	**Yes**	**1619**	**1273**	**1273**	**2420**	**1956**	**2268**	**2755**	**2528**	**2434**	**SAUSA300_0248**	**putative teichoic acid biosynthesis protein F**
**SAUSA300_0827**	**0,6**	**1,6**	**8,3E-09**	**Yes**	**361**	**371**	**367**	**655**	**553**	**548**	**548**	**522**	**471**	**SAUSA300_0827**	**hypothetical protein**
**SAUSA300_0624**	**0,6**	**1,6**	**1,6E-09**	**Yes**	**2367**	**2057**	**2279**	**3142**	**3896**	**3634**	**3323**	**3265**	**3458**	**tagH**	**teichoic acids export protein ATP-binding subunit**
**SAUSA300_0364**	**0,6**	**1,6**	**3,7E-04**	**Yes**	**869**	**1217**	**1412**	**2101**	**1892**	**1586**	**1552**	**1696**	**1499**	**ychF**	**GTP-dependent nucleic acid-binding protein EngD**
**SAUSA300_1124**	**0,6**	**1,6**	**1,0E-22**	**Yes**	**3082**	**3334**	**3528**	**5193**	**5430**	**5222**	**5908**	**6170**	**5576**	**fabG**	**3-oxoacyl-(acyl-carrier-protein) reductase**
**SAUSA300_0326**	**0,6**	**1,6**	**5,0E-05**	**Yes**	**104**	**142**	**147**	**221**	**217**	**194**	**182**	**209**	**208**	**SAUSA300_0326**	**hypothetical protein**
**SAUSA300_1659**	**0,6**	**1,6**	**1,1E-08**	**Yes**	**4639**	**4338**	**3547**	**6191**	**7159**	**6517**	**6579**	**5863**	**5782**	**tpx**	**thiol peroxidase**
**SAUSA300_0883**	**0,6**	**1,6**	**2,5E-03**	**Yes**	**255**	**283**	**208**	**401**	**454**	**326**	**440**	**349**	**417**	**SAUSA300_0883**	**putative surface protein**
**SAUSA300_1126**	**0,6**	**1,6**	**1,4E-04**	**Yes**	**184**	**273**	**337**	**429**	**447**	**392**	**426**	**434**	**470**	**rnc**	**ribonuclease III**
**SAUSA300_2644**	**0,6**	**1,6**	**1,6E-09**	**Yes**	**569**	**638**	**651**	**1098**	**957**	**896**	**1003**	**1039**	**1019**	**gidB**	**16S rRNA methyltransferase GidB**
**SAUSA300_1326**	**0,6**	**1,6**	**5,0E-03**	**Yes**	**185**	**166**	**155**	**192**	**362**	**241**	**306**	**270**	**260**	**SAUSA300_1326**	**putative cell wall enzyme EbsB**
**SAUSA300_2214**	**0,6**	**1,6**	**1,5E-06**	**Yes**	**1566**	**2071**	**2033**	**3160**	**3059**	**2710**	**2734**	**2827**	**2746**	**SAUSA300_2214**	**FmhB protein**
**SAUSA300_0969**	**0,6**	**1,6**	**9,8E-03**	**Yes**	**125**	**245**	**203**	**334**	**293**	**280**	**338**	**220**	**346**	**purS**	**phosphoribosylformylglycinamidine synthase**
**SAUSA300_0251**	**0,6**	**1,6**	**2,8E-13**	**Yes**	**5562**	**5367**	**5225**	**9094**	**8341**	**7941**	**9326**	**9555**	**9218**	**SAUSA300_0251**	**putative teichoic acid biosynthesis protein**
**SAUSA300_0904**	**0,6**	**1,6**	**1,2E-07**	**Yes**	**1097**	**1040**	**957**	**1712**	**1653**	**1484**	**1516**	**1426**	**1402**	**SAUSA300_0904**	**hypothetical protein**
**SAUSA300_2595**	**0,6**	**1,6**	**7,0E-03**	**Yes**	**53**	**53**	**40**	**81**	**77**	**70**	**74**	**81**	**73**	**SAUSA300_2595**	**acetyltransferase**
**SAUSA300_2323**	**0,6**	**1,6**	**9,0E-03**	**Yes**	**806**	**582**	**851**	**1019**	**916**	**1567**	**1141**	**1078**	**1438**	**cobI**	**CorA family protein**
**SAUSA300_0512**	**0,6**	**1,6**	**3,8E-04**	**Yes**	**646**	**634**	**797**	**1033**	**920**	**1296**	**867**	**773**	**996**	**SAUSA300_0512**	**PilT domain-containing protein**
**SAUSA300_1634**	**0,6**	**1,6**	**4,3E-07**	**Yes**	**941**	**1016**	**960**	**1574**	**1598**	**1389**	**1372**	**1260**	**1223**	**coaE**	**dephospho-CoA kinase**
**SAUSA300_2231**	**0,6**	**1,6**	**3,5E-05**	**Yes**	**1487**	**1210**	**1002**	**2078**	**1773**	**1916**	**2102**	**1706**	**1698**	**fdhD**	**formate dehydrogenase accessory protein**
**SAUSA300_0422**	**0,6**	**1,6**	**1,9E-04**	**Yes**	**707**	**608**	**504**	**874**	**1000**	**957**	**1430**	**1201**	**1358**	**SAUSA300_0422**	**hypothetical protein**
**SAUSA300_0945**	**0,6**	**1,6**	**3,0E-07**	**Yes**	**546**	**713**	**693**	**1112**	**976**	**966**	**1021**	**844**	**914**	**SAUSA300_0945**	**isochorismate synthase family protein**
**SAUSA300_1146**	**0,6**	**1,6**	**2,2E-03**	**Yes**	**486**	**546**	**772**	**1029**	**882**	**909**	**943**	**1143**	**1175**	**hslV**	**ATP-dependent protease peptidase subunit**

SAUSA300_1190	0,6	1,6	2,3E-07	Yes	318	292	330	486	494	485	495	450	485	glpP	glycerol uptake operon antiterminator regulatory protein
SAUSA300_0535	0,6	1,6	1,2E-07	Yes	777	864	1034	1357	1423	1395	1679	2000	1603	SAUSA300_0535	2-amino-3-ketobutyrate coenzyme A ligase
SAUSA300_0875	0,6	1,6	1,4E-06	Yes	1179	1183	1147	1517	2063	1880	1931	1961	1660	SAUSA300_0875	hypothetical protein
SAUSA300_1728	0,6	1,6	2,2E-04	Yes	421	542	398	683	782	651	602	517	540	SAUSA300_1728	aldo/keto reductase family oxidoreductase
SAUSA300_1854	0,6	1,6	9,6E-03	Yes	212	224	273	388	332	384	300	274	368	recX	recombination regulator RecX
SAUSA300_1872	0,6	1,6	1,5E-14	Yes	978	995	1041	1510	1615	1558	1371	1427	1369	SAUSA300_1872	hypothetical protein
SAUSA300_2450	0,6	1,6	5,6E-03	Yes	570	394	557	708	669	980	827	911	789	SAUSA300_2450	DedA family protein
SAUSA300_1189	0,6	1,5	1,6E-08	Yes	1477	1507	1519	2465	2105	2406	2216	2101	2342	mutL	DNA mismatch repair protein
SAUSA300_0636	0,6	1,5	9,3E-03	Yes	43	63	51	73	93	77	80	95	95	SAUSA300_0636	dihydroxyacetone kinase subunit DhaK
SAUSA300_2139	0,6	1,5	2,7E-03	Yes	110	111	150	188	163	225	166	166	210	SAUSA300_2139	putative transporter
SAUSA300_2002	0,6	1,5	3,7E-04	Yes	890	958	1040	1663	1362	1437	1373	1263	1323	SAUSA300_2002	putative DNA-binding/iron metalloprotein/AP endonuclease
SAUSA300_2535	0,6	1,5	1,7E-09	Yes	1329	1500	1246	2018	2230	2035	2154	2216	1981	panE	2-dehydropantoate 2-reductase
SAUSA300_2581	0,6	1,5	1,5E-07	Yes	486	626	567	897	903	792	940	879	1016	SAUSA300_2581	putative surface anchored protein
SAUSA300_0707	0,6	1,5	1,3E-08	Yes	2443	1907	2030	3378	3230	3199	3312	3128	3284	SAUSA300_0707	osmoprotectant ABC transporter permease
SAUSA300_1043	0,7	1,5	2,1E-14	Yes	1497	1647	1468	2573	2300	2223	1939	1817	1817	mutS2	recombination and DNA strand exchange inhibitor protein
SAUSA300_0252	0,7	1,5	9,2E-18	Yes	7886	7747	7729	12599	11488	11799	13320	13377	13409	SAUSA300_0252	glycosyl transferase, group 2 family protein
SAUSA300_2422	0,7	1,5	2,2E-05	Yes	412	514	521	765	791	667	674	630	677	SAUSA300_2422	short chain dehydrogenase/reductase family oxidoreductase
SAUSA300_2516	0,7	1,5	1,4E-03	Yes	130	127	115	225	187	158	160	142	146	SAUSA300_2516	short chain dehydrogenase/reductase family oxidoreductase
SAUSA300_2182	0,7	1,5	4,7E-07	Yes	2037	2304	2393	3695	3507	3106	3301	3535	3234	infA	translation initiation factor IF-1
SAUSA300_0626	0,7	1,5	4,1E-07	Yes	685	728	801	1176	1088	1124	1137	1255	1208	tagB	teichoic acid biosynthesis protein B
SAUSA300_1850	0,7	1,5	6,8E-07	Yes	1025	864	943	1266	1432	1622	1243	1337	1369	SAUSA300_1850	hypothetical protein
SAUSA300_2295	0,7	1,5	1,2E-07	Yes	392	346	361	558	557	560	520	564	602	SAUSA300_2295	hypothetical protein
SAUSA300_0780	0,7	1,5	1,5E-05	Yes	228	189	182	339	281	290	268	284	269	SAUSA300_0780	hypothetical protein
SAUSA300_2091	0,7	1,5	4,3E-05	Yes	5041	4457	4379	6595	7455	7122	6945	7194	5451	deoD	purine nucleoside phosphorylase
SAUSA300_1013	0,7	1,5	3,4E-06	Yes	2072	1716	1754	2529	2763	3143	2917	2853	2787	SAUSA300_1013	cell cycle protein FtsW
SAUSA300_0826	0,7	1,5	1,1E-07	Yes	726	732	677	1182	1040	1028	1095	1012	1027	SAUSA300_0826	hypothetical protein
SAUSA300_0726	0,7	1,5	7,8E-11	Yes	1058	1250	1070	1736	1765	1634	1415	1400	1506	SAUSA300_0726	glycerate kinase family protein
SAUSA300_1596	0,7	1,5	1,7E-04	Yes	772	1095	1149	1729	1537	1314	1189	1234	1290	queA	S-adenosylmethionine:tRNA ribosyltransferase-isomerase
SAUSA300_1351	0,7	1,5	1,2E-05	Yes	16366	15675	14089	19845	25822	24185	23517	23498	18249	SAUSA300_1351	hypothetical protein
SAUSA300_1982	0,7	1,5	1,2E-03	Yes	3938	5580	3474	6902	6404	6351	6725	6187	5609	groEL	chaperonin GroEL
SAUSA300_0250	0,7	1,5	7,5E-09	Yes	3159	3225	3002	5036	4458	4704	4965	5108	5006	SAUSA300_0250	alcohol dehydrogenase, zinc-containing
SAUSA300_0102	0,7	1,5	6,5E-03	Yes	68	65	73	124	96	94	126	137	147	SAUSA300_0102	tandem lipoprotein
SAUSA300_1902	0,7	1,5	2,6E-08	Yes	1530	1382	1395	2071	2255	2177	1989	1974	1903	SAUSA300_1902	hypothetical protein
SAUSA300_0685	0,7	1,5	2,6E-04	Yes	2745	3249	3769	5405	4729	4616	5795	7294	5483	fruA	fructose specific permease
SAUSA300_0991	0,7	1,5	2,4E-06	Yes	2339	2019	1902	3007	3413	3006	3023	2819	2664	def	peptide deformylase
SAUSA300_0477	0,7	1,5	9,2E-04	Yes	2052	3233	2651	4150	4478	3338	3220	3529	3039	glmU	bifunctional N-acetylglucosamine-1-phosphate uridyltransferase/glucosamine-1-phosphate acetyltransferase
SAUSA300_1347	0,7	1,5	1,3E-05	Yes	714	723	728	1146	1029	1090	878	845	1011	birA	BirA bifunctional protein
SAUSA300_1023	0,7	1,5	7,8E-07	Yes	382	370	360	561	500	611	475	474	491	SAUSA300_1023	hypothetical protein
SAUSA300_1861	0,7	1,5	7,8E-04	Yes	140	164	177	223	283	218	269	288	269	SAUSA300_1861	hypothetical protein
SAUSA300_0030	0,7	1,5	3,7E-07	Yes	702	793	720	1086	1176	1070	1192	1138	1118	SAUSA300_0030	putative glycerophosphoryl diester phosphodiesterase
SAUSA300_0730	0,7	1,5	2,7E-03	Yes	454	420	519	653	537	903	642	620	754	SAUSA300_0730	GGDEF domain-containing protein
SAUSA300_1873	0,7	1,5	3,3E-10	Yes	1572	1512	1508	2514	2068	2306	1969	1972	1891	SAUSA300_1873	Mur ligase family protein
SAUSA300_1261	0,7	1,5	2,3E-11	Yes	620	612	585	910	938	872	808	808	733	SAUSA300_1261	putative glutamyl aminopeptidase
SAUSA300_1786	0,7	1,5	2,5E-08	Yes	1544	1456	1399	2189	2201	2195	2388	2340	2101	SAUSA300_1786	ABC transporter ATP-binding protein EcsA

SAUSA300_1901	0,7	1,5	1,5E-07	Yes	2551	2511	2075	3612	3706	3354	3159	3051	2965	aldA2	aldehyde dehydrogenase
SAUSA300_1660	0,7	1,5	4,6E-03	Yes	131	150	191	282	220	213	199	221	234	SAUSA300_1660	hypothetical protein
SAUSA300_1495	0,7	1,5	1,8E-04	Yes	2367	2353	2237	2745	4169	3487	3390	3259	2614	SAUSA300_1495	hypothetical protein
SAUSA300_0825	0,7	1,5	2,4E-03	Yes	863	1226	991	1576	1700	1334	1254	1244	1303	SAUSA300_0825	2-nitropropane dioxygenase family oxidoreductase
SAUSA300_0902	0,7	1,5	2,6E-05	Yes	5702	5924	4900	8159	8364	8166	7693	6951	7120	pepF	oligoendopeptidase F
SAUSA300_1691	0,7	1,5	3,8E-12	Yes	1115	1006	920	1537	1510	1464	1499	1476	1387	SAUSA300_1691	glutamyl-aminopeptidase
SAUSA300_0706	0,7	1,5	1,1E-09	Yes	1713	1547	1430	2488	2328	2138	2374	2262	2227	SAUSA300_0706	putative osmoprotectant ABC transporter ATP-binding protein
SAUSA300_0366	0,7	1,5	9,2E-03	Yes	3421	4696	5559	7663	6897	5731	6661	7351	6095	rpsF	30S ribosomal protein S6
SAUSA300_1042	0,7	1,5	3,0E-06	Yes	896	816	873	1431	1241	1163	1070	943	1039	SAUSA300_1042	hypothetical protein
SAUSA300_1123	0,7	1,5	8,0E-10	Yes	3243	3463	4024	5555	5355	4995	5298	5821	5460	fabD	malonyl CoA-acyl carrier protein transacylase
SAUSA300_0020	0,7	1,5	7,7E-10	Yes	1583	1590	1575	2356	2376	2289	3044	3111	2860	SAUSA300_0020	DNA-binding response regulator
SAUSA300_0744	0,7	1,5	1,9E-05	Yes	488	664	673	888	978	839	815	877	836	lgt	prolipoprotein diacylglyceryl transferase
SAUSA300_1886	0,7	1,5	3,1E-08	Yes	2440	3098	2611	4254	4096	3690	3503	3362	3315	pcrA	ATP-dependent DNA helicase, PcrA
SAUSA300_1635	0,7	1,5	6,1E-08	Yes	757	920	879	1341	1204	1238	1089	1000	1013	mutM	formamidopyrimidine-DNA glycosylase
SAUSA300_0439	0,7	1,5	9,9E-03	Yes	375	342	382	404	663	552	649	554	463	SAUSA300_0439	hypothetical protein
SAUSA300_2213	0,7	1,5	7,2E-07	Yes	3453	3669	3929	5860	5443	5010	5702	5681	5528	SAUSA300_2213	AcrB/AcrD/AcrF family protein
SAUSA300_2540	0,7	1,5	2,5E-04	Yes	10836	7916	8047	11552	13713	14107	14292	15310	13666	SAUSA300_2540	fructose-1,6-bisphosphate aldolase
SAUSA300_0073	0,7	1,5	8,2E-05	Yes	599	759	568	1060	931	842	924	899	863	SAUSA300_0073	peptide ABC transporter peptide-binding protein
SAUSA300_0526	0,7	1,5	8,2E-09	Yes	1332	1319	1354	2249	1769	1863	1765	1693	1714	SAUSA300_0526	methyltransferase small subunit
SAUSA300_2194	0,7	1,5	9,4E-03	Yes	3836	4833	5023	7783	6485	5797	7037	7512	6072	rplN	50S ribosomal protein L14
SAUSA300_1242	0,7	1,5	6,6E-05	Yes	255	324	278	414	465	378	401	394	438	sbcD	exonuclease SbcD
SAUSA300_2188	0,7	1,5	2,5E-03	Yes	4114	5393	5476	8658	6955	6310	7100	7459	6645	rplR	50S ribosomal protein L18
SAUSA300_0509	0,7	1,5	2,8E-05	Yes	1207	1546	1184	2071	1818	1870	1677	1551	1692	SAUSA300_0509	ATP:guanido phosphotransferase
SAUSA300_0803	0,7	1,5	5,2E-04	Yes	690	749	569	1166	868	904	848	803	784	SAUSA300_0803	Cro/CI family transcriptional regulator
SAUSA300_2005	0,7	1,5	1,4E-03	Yes	358	348	344	468	457	607	517	403	509	SAUSA300_2005	hypothetical protein
SAUSA300_0090	0,7	1,5	6,2E-03	Yes	78	81	91	138	129	103	131	156	124	SAUSA300_0090	hypothetical protein
SAUSA300_0857	0,7	1,5	2,2E-06	Yes	2133	2571	2252	3187	3842	3126	2739	3026	2741	SAUSA300_0857	hypothetical protein
SAUSA300_1668	0,7	1,5	1,1E-04	Yes	582	517	445	663	815	766	707	647	657	SAUSA300_1668	OsmC/Ohr family protein
SAUSA300_1843	0,7	1,5	2,8E-05	Yes	876	890	834	1184	1328	1273	1130	1153	1169	SAUSA300_1843	D-isomer specific 2-hydroxyacid dehydrogenase family protein
SAUSA300_1460	0,7	1,5	5,0E-04	Yes	1120	1528	1216	1783	2108	1738	1521	1474	1497	SAUSA300_1460	M20/M25/M40 family peptidase
SAUSA300_1335	0,7	1,5	8,2E-03	Yes	173	140	183	223	199	299	237	199	241	SAUSA300_1335	hypothetical protein
SAUSA300_2353	0,7	1,5	5,9E-03	Yes	519	558	439	544	892	764	801	776	735	SAUSA300_2353	hypothetical protein
SAUSA300_1051	0,7	1,5	9,4E-03	Yes	1975	1571	1723	2252	2533	2876	2281	2163	2513	SAUSA300_1051	hypothetical protein
SAUSA300_1620	0,7	1,5	6,1E-05	Yes	388	397	351	521	558	567	480	527	514	engB	ribosome biogenesis GTP-binding protein YsxC
SAUSA300_2220	0,7	1,5	1,4E-03	Yes	245	233	337	428	347	416	355	324	323	mobA	molybdopterin-guanine dinucleotide biosynthesis protein MobA
SAUSA300_0659	0,7	1,5	1,5E-09	Yes	931	899	879	1289	1245	1395	1173	1192	1144	SAUSA300_0659	sugar efflux transporter
SAUSA300_1733	0,7	1,4	1,4E-03	Yes	231	245	225	388	340	289	285	307	278	SAUSA300_1733	hypothetical protein
SAUSA300_0948	0,7	1,4	4,1E-05	Yes	4076	4580	4394	6118	6450	6314	5644	6189	5178	menB	naphthoate synthase
SAUSA300_1349	0,7	1,4	4,7E-08	Yes	703	689	757	1138	924	1048	924	860	889	SAUSA300_1349	glycosyl transferase, group 1 family protein
SAUSA300_2184	0,7	1,4	9,0E-05	Yes	15599	18572	18682	29630	25262	21401	25775	26861	23217	secY	preprotein translocase subunit SecY
SAUSA300_2181	0,7	1,4	8,4E-03	Yes	1651	1905	1799	3023	2320	2385	2274	2224	2541	rpmJ	50S ribosomal protein L36
SAUSA300_2130	0,7	1,4	7,5E-05	Yes	1098	1146	1537	2007	1617	1842	1711	1758	1780	SAUSA300_2130	UTP-glucose-1-phosphate uridylyltransferase family protein
SAUSA300_2526	0,7	1,4	5,9E-08	Yes	2088	1780	1955	2824	2686	2879	3407	3291	3412	pyrD	dihydroorotate dehydrogenase 2
SAUSA300_1147	0,7	1,4	4,3E-05	Yes	2687	3324	3662	5082	4659	4182	4925	5894	5721	hslU	ATP-dependent protease ATP-binding subunit HslU

SAUSA300_1451	0,7	1,4	1,0E-06	Yes	418	500	426	657	647	630	483	488	553	SAUSA300_1451	short chain dehydrogenase/reductase family oxidoreductase
SAUSA300_1246	0,7	1,4	1,6E-04	Yes	4267	4481	3882	6250	6596	5299	6786	6217	5226	acnA	aconitate hydratase
SAUSA300_1024	0,7	1,4	1,5E-04	Yes	483	468	439	613	620	756	554	534	558	coaD	phosphopantetheine adenylyltransferase
SAUSA300_2201	0,7	1,4	7,5E-03	Yes	12971	14913	15872	23759	20055	18959	21874	23818	19449	rplB	50S ribosomal protein L2
SAUSA300_0508	0,7	1,4	3,9E-04	Yes	633	794	572	1063	1000	804	872	911	838	SAUSA300_0508	hypothetical protein
SAUSA300_2203	0,7	1,4	6,5E-03	Yes	7403	7883	8847	13710	9935	10936	11486	12778	11405	rplD	50S ribosomal protein L4
SAUSA300_1045	0,7	1,4	7,0E-04	Yes	595	720	563	959	940	791	749	679	706	uvrC	excinuclease ABC subunit C
SAUSA300_2003	0,7	1,4	1,8E-03	Yes	431	413	441	697	502	642	587	511	641	rimI	ribosomal-protein-alanine acetyltransferase
SAUSA300_1243	0,7	1,4	1,9E-06	Yes	659	726	752	973	993	1087	918	837	976	sbcC	exonuclease SbcC
SAUSA300_0747	0,7	1,4	9,8E-03	Yes	2158	2940	3312	4507	4249	3237	3296	3178	3322	trxB	thioredoxin-disulfide reductase
SAUSA300_2260	0,7	1,4	4,2E-03	Yes	160	160	224	256	281	242	230	274	243	SAUSA300_2260	inositol monophosphatase family protein
SAUSA300_0072	0,7	1,4	3,1E-04	Yes	282	318	227	407	400	366	373	351	338	SAUSA300_0072	hypothetical protein
SAUSA300_0867	0,7	1,4	1,6E-04	Yes	1114	843	1046	1281	1408	1577	1427	1348	1371	spsA	signal peptidase IA
SAUSA300_2185	0,7	1,4	3,1E-03	Yes	4062	4995	4830	7809	6136	5811	7001	7197	5986	rplO	50S ribosomal protein L15
SAUSA300_0789	0,7	1,4	5,5E-03	Yes	1733	1495	1134	1891	2391	1912	2062	1823	1464	SAUSA300_0789	putative thioredoxin
SAUSA300_1145	0,7	1,4	2,6E-03	Yes	556	593	902	1019	947	953	990	1138	1149	xerC	tyrosine recombinase xerC
SAUSA300_0824	0,7	1,4	3,9E-03	Yes	613	827	648	1086	1049	830	780	741	746	SAUSA300_0824	hypothetical protein
SAUSA300_2046	0,7	1,4	2,5E-04	Yes	9769	7284	8656	11403	11442	13608	14442	14019	12851	oxaA	membrane protein oxaA
SAUSA300_0021	0,7	1,4	9,6E-08	Yes	4251	4542	4223	6317	6090	6029	7294	7650	7091	SAUSA300_0021	sensory box histidine kinase
SAUSA300_1654	0,7	1,4	1,1E-03	Yes	1614	1713	1384	2260	2402	1999	1896	1860	1632	SAUSA300_1654	proline dipeptidase
SAUSA300_0839	0,7	1,4	9,5E-03	Yes	1622	1643	1497	1716	2645	2368	2213	2219	1818	SAUSA300_0839	hypothetical protein
SAUSA300_1122	0,7	1,4	3,3E-07	Yes	2884	3199	3609	4924	4490	4305	4926	5025	4667	plsX	putative glycerol-3-phosphate acyltransferase PlsX
SAUSA300_0943	0,7	1,4	5,6E-04	Yes	318	347	363	515	523	419	414	481	431	SAUSA300_0943	acetyltransferase
SAUSA300_1104	0,7	1,4	4,0E-06	Yes	777	936	833	1296	1256	1052	943	899	855	coaBC	phosphopantothenoylcysteine decarboxylase/phosphopantothenate--cysteine ligase
SAUSA300_0074	0,7	1,4	5,0E-04	Yes	418	451	347	613	594	507	579	539	475	opp-3B	oligopeptide permease, channel-forming protein
SAUSA300_2192	0,7	1,4	7,0E-03	Yes	4902	5969	6654	9804	8170	6758	8217	8959	7709	rplE	50S ribosomal protein L5
SAUSA300_1127	0,7	1,4	6,9E-03	Yes	1412	1952	2093	2913	2487	2303	2385	2264	2662	smc	chromosome segregation protein SMC
SAUSA300_2382	0,7	1,4	7,7E-03	Yes	230	203	173	272	303	272	287	253	252	SAUSA300_2382	hypothetical protein
SAUSA300_1624	0,7	1,4	1,3E-03	Yes	513	752	746	967	1024	847	812	773	789	SAUSA300_1624	MutT/nudix family protein
SAUSA300_0349	0,7	1,4	5,6E-03	Yes	1392	1041	1015	1647	1631	1567	1837	1558	1326	SAUSA300_0349	hypothetical protein
SAUSA300_0511	0,7	1,4	9,5E-05	Yes	1727	2339	2195	3257	2803	2756	2318	2337	2303	radA	DNA repair protein RadA
SAUSA300_1994	0,7	1,4	2,3E-03	Yes	1363	1983	1963	2598	2768	2102	2158	2415	2320	scrB	sucrose-6-phosphate hydrolase
SAUSA300_1688	0,7	1,4	1,5E-05	Yes	665	658	644	930	833	995	777	724	764	SAUSA300_1688	phenylalanyl-tRNA synthetase (beta subunit)
SAUSA300_0763	0,7	1,4	3,0E-08	Yes	836	902	948	1326	1200	1249	1252	1191	1159	est	carboxylesterase
SAUSA300_0510	0,7	1,4	2,0E-07	Yes	5331	5717	4826	7958	7030	7242	7390	6693	6717	clpC	endopeptidase
SAUSA300_1346	0,7	1,4	3,5E-06	Yes	2163	1987	2198	2748	2734	3383	2472	2507	2639	SAUSA300_1346	DnaQ family exonuclease/DinG family helicase
SAUSA300_0190	0,7	1,4	7,7E-04	Yes	1756	1573	1261	2224	2228	1951	2569	2351	2575	ipdC	indole-3-pyruvate decarboxylase
SAUSA300_1121	0,7	1,4	2,4E-04	Yes	1231	1135	1531	1883	1584	1982	2060	1986	2002	SAUSA300_1121	fatty acid biosynthesis transcriptional regulator
SAUSA300_0705	0,7	1,4	7,1E-05	Yes	2283	2301	2535	3503	3145	3299	2858	3006	3131	recQ	ATP-dependent DNA helicase RecQ
SAUSA300_1340	0,7	1,4	3,0E-12	Yes	2350	2294	2289	3043	3234	3387	3221	3316	3241	recU	Holliday junction-specific endonuclease
SAUSA300_0947	0,7	1,4	1,8E-04	Yes	1001	880	1097	1491	1307	1356	1252	1319	1307	SAUSA300_0947	alpha/beta fold family hydrolase
SAUSA300_1867	0,7	1,4	1,2E-07	Yes	592	581	531	796	752	819	681	640	650	SAUSA300_1867	hypothetical protein
SAUSA300_1148	0,7	1,4	1,1E-05	Yes	1979	1961	2203	2912	2705	2922	3138	3394	3507	codY	transcriptional repressor CodY
SAUSA300_1374	0,7	1,4	9,5E-03	Yes	1215	1122	1020	1249	1741	1669	1489	1359	1489	SAUSA300_1374	hypothetical protein

SAUSA300_0886	0,7	1,4	5,0E-04	Yes	6011	7861	8077	10620	10868	9011	10998	11593	10135	fabF	3-oxoacyl-(acyl-carrier-protein) synthase II
SAUSA300_2580	0,7	1,4	2,7E-04	Yes	954	861	728	1162	1247	1116	1372	1393	1240	SAUSA300_2580	isochorismatase family protein
SAUSA300_1889	0,7	1,4	1,4E-04	Yes	4199	4747	3422	5190	6030	5923	6168	5500	5672	purB	adenylosuccinate lyase
SAUSA300_1069	0,7	1,4	2,0E-04	Yes	247	263	285	392	357	359	308	316	333	SAUSA300_1069	hypothetical protein
SAUSA300_1693	0,7	1,4	1,8E-04	Yes	493	506	498	638	791	641	747	794	637	SAUSA300_1693	hypothetical protein
SAUSA300_0871	0,7	1,4	8,8E-05	Yes	7215	7076	7105	9420	10734	9407	9059	9655	8545	SAUSA300_0871	hypothetical protein
SAUSA300_2183	0,7	1,4	6,6E-03	Yes	7673	9364	8896	14302	11364	10167	12665	13030	11205	adk	adenylate kinase
SAUSA300_1735	0,7	1,4	4,2E-03	Yes	314	454	432	620	550	493	418	434	456	menC	O-succinylbenzoic acid synthetase
SAUSA300_1737	0,7	1,4	4,7E-03	Yes	434	620	542	833	757	616	530	561	529	menE	O-succinylbenzoate-CoA ligase
SAUSA300_1105	0,7	1,4	2,8E-06	Yes	2099	2559	2177	3394	3195	2839	2649	2695	2460	priA	primosomal protein N`
SAUSA300_2527	0,7	1,4	1,5E-03	Yes	928	825	853	1132	1259	1197	1397	1292	1397	SAUSA300_2527	hypothetical protein
SAUSA300_2628	0,7	1,4	1,4E-04	Yes	562	594	550	768	894	687	781	801	698	rarD	RarD protein
SAUSA300_1636	0,7	1,4	1,6E-04	Yes	2474	2803	2554	3770	3587	3406	3083	2877	2948	polA	DNA polymerase I superfamily protein
SAUSA300_0743	0,7	1,4	5,0E-05	Yes	1209	1351	1493	2004	1929	1640	1626	1717	1802	hprK	HPr kinase/phosphorylase
SAUSA300_1155	0,7	1,4	1,5E-04	Yes	1987	2202	2188	3043	3143	2567	2349	2400	2282	SAUSA300_1155	putative membrane-associated zinc metalloprotease
SAUSA300_1049	0,7	1,4	1,1E-05	Yes	2265	2310	2006	3081	3082	2858	2964	2644	2542	murI	glutamate racemase
SAUSA300_1887	0,7	1,4	1,1E-05	Yes	616	793	705	960	987	958	860	836	776	pcrB	geranylgeranylglyceryl phosphate synthase-like protein
SAUSA300_1467	0,7	1,4	2,1E-09	Yes	3303	3716	3262	4634	4735	4724	4612	4905	4677	lpdA	dihydrolipoamide dehydrogenase
SAUSA300_1343	0,7	1,4	8,3E-03	Yes	881	881	630	1031	1110	1129	943	784	838	nth	endonuclease III
SAUSA300_0023	0,7	1,4	3,9E-07	Yes	2442	2133	2188	3039	2985	3193	3684	3697	3720	SAUSA300_0023	hypothetical protein
SAUSA300_1781	0,7	1,4	6,8E-05	Yes	1231	1492	1403	2031	1908	1690	1557	1457	1555	hemG	protoporphyrinogen oxidase
SAUSA300_2583	0,7	1,4	2,2E-03	Yes	896	910	1163	1526	1294	1229	1651	1673	1764	SAUSA300_2583	putative glycosyl transferase
SAUSA300_0249	0,7	1,4	6,1E-04	Yes	1697	1674	1621	2500	2192	2094	2592	2411	2492	ispD	2-C-methyl-D-erythritol 4-phosphate cytidylyltransferase
SAUSA300_0005	0,7	1,4	1,1E-03	Yes	3890	5341	4767	6906	6604	5522	7098	6873	6540	gyrB	DNA gyrase subunit B
SAUSA300_1605	0,7	1,4	2,9E-03	Yes	801	937	912	1279	1262	1059	1049	1005	997	mreC	rod shape-determining protein MreC
SAUSA300_1884	0,7	1,4	7,8E-06	Yes	2738	2736	2734	3846	3734	3553	3350	3254	3282	SAUSA300_1884	CamS sex pheromone cAM373
SAUSA300_1783	0,7	1,4	6,2E-03	Yes	873	1063	936	1400	1479	1019	1101	1080	1081	hemE	uroporphyrinogen decarboxylase
SAUSA300_2146	0,7	1,4	1,7E-04	Yes	685	756	573	892	979	854	874	837	777	SAUSA300_2146	alcohol dehydrogenase, zinc-containing
SAUSA300_0507	0,7	1,4	1,5E-05	Yes	506	547	503	685	688	735	676	644	616	ctsR	transcriptional regulator CtsR
SAUSA300_0958	0,7	1,4	3,6E-05	Yes	5918	5001	5215	7395	6832	7610	7566	7529	7598	SAUSA300_0958	hypothetical protein
SAUSA300_1686	0,7	1,4	2,5E-06	Yes	3287	3051	3296	4488	3994	4537	4031	4115	4190	murC	UDP-N-acetylmuramate--L-alanine ligase
SAUSA300_2068	0,7	1,4	4,9E-03	Yes	1322	1906	1847	2378	2519	1967	2080	2176	1908	SAUSA300_2068	hypothetical protein
SAUSA300_0527	0,7	1,4	1,4E-04	Yes	46072	51007	42511	68735	61597	58177	61737	58525	56547	rpoB	DNA-directed RNA polymerase subunit beta
SAUSA300_1522	0,7	1,3	1,4E-03	Yes	3172	2819	3014	3626	3719	4799	4354	3940	4201	dnaG	DNA primase
SAUSA300_0704	0,7	1,3	2,2E-03	Yes	2263	2250	2572	3355	2948	3254	3291	3036	2845	SAUSA300_0704	ABC transporter ATP-binding protein
SAUSA300_0022	0,7	1,3	1,8E-05	Yes	4488	4635	4022	6240	5610	5835	7008	7007	6649	SAUSA300_0022	hypothetical protein
SAUSA300_1579	0,7	1,3	9,1E-04	Yes	3093	3390	2634	4067	4292	3900	3386	3058	3065	SAUSA300_1579	aminotransferase, class V
SAUSA300_0946	0,7	1,3	7,0E-04	Yes	1536	1463	1538	2207	1948	1944	1842	1770	1830	menD	2-succinyl-6-hydroxy-2, 4-cyclohexadiene-1-carboxylic acid synthase/2-oxoglutarate decarboxylase
SAUSA300_2589	0,7	1,3	7,1E-05	Yes	23639	20777	20326	30034	26358	30390	41032	46977	47655	SAUSA300_2589	cell wall anchor domain-containing protein
SAUSA300_0802	0,7	1,3	1,0E-03	Yes	1031	1117	800	1427	1312	1208	1164	1199	1126	SAUSA300_0802	hypothetical protein
SAUSA300_1548	0,7	1,3	3,7E-03	Yes	676	717	614	782	1040	861	620	633	598	SAUSA300_1548	ComE operon protein 2
SAUSA300_1661	0,7	1,3	6,7E-04	Yes	454	467	542	736	612	618	560	601	617	thiI	thiamine biosynthesis protein ThiI
SAUSA300_2519	0,7	1,3	2,5E-03	Yes	168	166	162	217	211	234	251	233	252	SAUSA300_2519	putative cobalamin synthesis protein
SAUSA300_0517	0,8	1,3	4,7E-03	Yes	599	705	764	1076	930	759	844	920	820	SAUSA300_0517	RNA methyltransferase

SAUSA300_0853	0,8	1,3	4,6E-03	Yes	1031	986	1033	1343	1433	1285	1122	1157	1124	mnhC	putative monovalent cation/H+ antiporter subunit C
SAUSA300_0752	0,8	1,3	2,8E-05	Yes	6890	5636	5398	7452	8170	8195	7847	7648	7376	clpP	ATP-dependent Clp protease proteolytic subunit
SAUSA300_0703	0,8	1,3	6,8E-03	Yes	17404	13580	15441	20086	17598	24025	23016	22951	24954	SAUSA300_0703	sulfatase family protein
SAUSA300_2219	0,8	1,3	1,4E-03	Yes	1369	1469	1304	1959	1862	1681	1779	1717	1540	moaA	molybdenum cofactor biosynthesis protein A
SAUSA300_1465	0,8	1,3	1,9E-06	Yes	2635	2772	2337	3504	3403	3362	3544	3452	3321	SAUSA300_1465	2-oxoisovalerate dehydrogenase, E1 component, beta subunit
SAUSA300_1120	0,8	1,3	6,8E-05	Yes	1259	1291	1146	1622	1569	1690	1392	1366	1300	recG	ATP-dependent DNA helicase RecG
SAUSA300_1336	0,8	1,3	4,7E-05	Yes	1078	1045	1022	1325	1322	1501	1431	1377	1364	SAUSA300_1336	hypothetical protein
SAUSA300_0912	0,8	1,3	4,2E-13	Yes	4223	4304	4460	5460	5816	5868	5261	5381	5053	SAUSA300_0912	enoyl-(acyl carrier protein) reductase
SAUSA300_0745	0,8	1,3	2,4E-04	Yes	421	467	456	666	564	550	518	558	522	SAUSA300_0745	putative acetyltransferase
SAUSA300_0528	0,8	1,3	2,5E-03	Yes	46520	51426	45520	70834	59029	59206	60902	62302	55316	rpoC	DNA-directed RNA polymerase subunit beta'
SAUSA300_1543	0,8	1,3	3,2E-03	Yes	281	319	305	414	357	425	367	343	358	SAUSA300_1543	coproporphyrinogen III oxidase
SAUSA300_1622	0,8	1,3	1,4E-03	Yes	17768	18470	16191	23380	23896	21738	20857	20706	18025	tig	trigger factor
SAUSA300_1216	0,8	1,3	6,1E-03	Yes	975	884	1113	1164	1248	1495	1226	1214	1228	SAUSA300_1216	cardiolipin synthetase
SAUSA300_1156	0,8	1,3	1,4E-04	Yes	4502	4997	4957	6943	6279	5780	4700	5026	4672	proS	prolyl-tRNA synthetase
SAUSA300_0732	0,8	1,3	8,0E-05	Yes	615	588	577	699	800	829	737	695	678	SAUSA300_0732	hypothetical protein
SAUSA300_0833	0,8	1,3	5,6E-05	Yes	1291	1485	1420	1811	1968	1724	1593	1664	1583	SAUSA300_0833	hypothetical protein
SAUSA300_1050	0,8	1,3	8,8E-05	Yes	2035	1800	1659	2517	2343	2320	2229	2022	2088	SAUSA300_1050	nucleoside-triphosphatase
SAUSA300_0013	0,8	1,3	2,9E-05	Yes	2206	1986	1943	2575	2526	2905	2895	2868	2852	SAUSA300_0013	hypothetical protein
SAUSA300_0834	0,8	1,3	2,7E-07	Yes	1680	1836	1763	2202	2399	2297	2004	2085	1930	SAUSA300_0834	D-isomer specific 2-hydroxyacid dehydrogenase
SAUSA300_1540	0,8	1,3	7,0E-03	Yes	8356	10470	8204	12524	11615	11130	11898	11417	11239	dnaK	molecular chaperone DnaK
SAUSA300_0024	0,8	1,3	5,9E-03	Yes	1108	892	935	1122	1306	1384	1578	1503	1422	SAUSA300_0024	metallo-beta-lactamase family protein
SAUSA300_1464	0,8	1,3	1,9E-06	Yes	3902	4128	3487	5002	4943	5019	5371	5093	4918	SAUSA300_1464	2-oxoisovalerate dehydrogenase, E2 component, dihydrolipoamide acetyltransferase
SAUSA300_0515	0,8	1,3	9,7E-03	Yes	823	1094	1128	1476	1307	1182	1129	1283	1256	cysS	cysteinyl-tRNA synthetase
SAUSA300_1025	0,8	1,3	5,2E-03	Yes	700	542	569	763	689	891	787	745	734	SAUSA300_1025	hypothetical protein
SAUSA300_0657	0,8	1,3	4,8E-04	Yes	545	537	618	686	708	812	734	699	705	SAUSA300_0657	hypothetical protein
SAUSA300_1782	0,8	1,3	9,9E-03	Yes	692	938	804	1164	1127	870	802	885	889	hemH	ferrochelatase
SAUSA300_1466	0,8	1,3	4,6E-05	Yes	2973	3327	2699	4062	3856	3728	4197	4004	3768	SAUSA300_1466	2-oxoisovalerate dehydrogenase, E1 component, alpha subunit
SAUSA300_0869	0,8	1,3	1,2E-04	Yes	1529	1737	1678	2399	1957	2045	1757	1682	1813	rexB	exonuclease RexB
SAUSA300_1468	0,8	1,3	3,9E-03	Yes	2563	2818	2781	3608	3267	3668	3191	3394	3711	recN	DNA repair protein RecN
SAUSA300_1687	0,8	1,3	4,2E-05	Yes	10993	11174	9605	14670	13492	12656	12561	12396	12358	SAUSA300_1687	FtsK/SpoIIIE family protein
SAUSA300_1612	0,8	1,3	7,7E-07	Yes	1092	1053	1054	1306	1351	1437	1344	1391	1373	tag	DNA-3-methyladenine glycosidase
SAUSA300_1178	0,8	1,3	8,0E-03	Yes	9210	8277	7275	11709	9586	10425	9835	10803	9961	recA	recombinase A
SAUSA300_1341	0,8	1,3	3,6E-06	Yes	18632	18737	18031	23872	22954	24019	21559	22975	21939	pbp2	penicillin binding protein 2
SAUSA300_0874	0,8	1,3	8,5E-03	Yes	1288	1034	1272	1544	1395	1655	1417	1430	1554	SAUSA300_0874	hypothetical protein
SAUSA300_0014	0,8	1,3	1,0E-03	Yes	8858	9170	8116	11024	11119	11250	12558	13085	11788	SAUSA300_0014	hypothetical protein
SAUSA300_0643	0,8	1,3	2,9E-04	Yes	1718	1527	1467	2075	2095	1843	2209	2250	2009	SAUSA300_0643	acetyltransferase
SAUSA300_0466	0,8	1,3	3,4E-03	Yes	315	329	310	389	443	383	392	371	353	SAUSA300_0466	hypothetical protein
SAUSA300_1115	0,8	1,3	4,7E-03	Yes	690	731	724	909	875	937	802	874	815	rpe	ribulose-phosphate 3-epimerase
SAUSA300_1878	0,8	1,3	2,1E-03	Yes	629	621	536	754	715	786	775	695	690	rumA	RNA methyltransferase
SAUSA300_0855	0,8	1,3	2,9E-04	Yes	5405	5421	5814	7054	6482	7460	5526	5950	5950	mnhA	monovalent cation/H+ antiporter subunit A
SAUSA300_1165	0,8	1,3	1,1E-03	Yes	1544	1633	1369	1857	1966	1906	1795	1854	1830	ribF	riboflavin biosynthesis protein ribF
SAUSA300_0911	0,8	1,3	1,8E-05	Yes	1400	1400	1484	1795	1716	1893	1693	1553	1566	SAUSA300_0911	transporter, monovalent cation:proton antiporter-2 (CPA2) family protein
SAUSA300_1866	0,8	1,3	5,8E-03	Yes	1935	1925	1772	2603	2058	2419	2012	1926	2045	vraS	two-component sensor histidine kinase
SAUSA300_1118	0,8	1,3	1,3E-05	Yes	1142	1197	1201	1445	1457	1537	1610	1479	1488	SAUSA300_1118	hypothetical protein

SAUSA300_1114	0,8	1,3	4,8E-03	Yes	647	622	713	813	755	918	856	800	836	rsgA	hypothetical protein
SAUSA300_1993	0,8	1,2	4,6E-05	Yes	1057	1172	1146	1407	1423	1391	1306	1333	1321	SAUSA300_1993	PfkB family kinase
SAUSA300_0483	0,8	1,2	1,6E-03	Yes	2175	2742	2326	3194	3050	2803	2677	2637	2566	SAUSA300_0483	tetrapyrrole methylase family protein
SAUSA300_1865	0,8	1,2	2,4E-03	Yes	1750	1746	1513	2144	2133	1960	1942	1928	1807	vraR	DNA-binding response regulator
SAUSA300_1119	0,8	1,2	1,8E-08	Yes	7392	7549	7142	8919	9459	9097	9673	9616	9173	SAUSA300_1119	hypothetical protein
SAUSA300_1470	0,8	1,2	1,9E-04	Yes	3048	3457	3095	4133	4069	3736	3704	3493	3511	SAUSA300_1470	geranyltranstransferase
SAUSA300_0669	0,8	1,2	6,7E-03	Yes	4063	4618	3707	4966	5690	4686	4571	4815	4140	SAUSA300_0669	undecaprenyl pyrophosphate phosphatase
SAUSA300_1869	0,8	1,2	5,9E-04	Yes	2616	2466	2449	3015	3122	3149	3204	3244	2932	map	methionine aminopeptidase
SAUSA300_1239	0,8	1,2	4,6E-03	Yes	17752	18081	14852	19729	22735	19989	20036	20177	17354	tkt	transketolase
SAUSA300_0944	0,8	1,2	2,0E-04	Yes	2055	2189	2131	2704	2673	2464	2184	2259	2473	menA	1,4-dihydroxy-2-naphthoate octaprenyltransferase
SAUSA300_2504	0,8	1,2	1,8E-04	Yes	5873	5583	5669	7097	6385	7503	7833	8438	8215	SAUSA300_2504	acyltransferase
SAUSA300_0870	0,8	1,2	1,4E-03	Yes	3411	3796	3247	4566	4226	4022	3832	3692	3684	rexA	exonuclease RexA
SAUSA300_1116	0,8	1,2	5,7E-03	Yes	706	819	751	872	938	958	887	972	884	SAUSA300_1116	hypothetical protein
SAUSA300_1570	0,8	1,2	6,6E-03	Yes	1758	1864	1709	2374	2074	2002	1792	1665	1706	SAUSA300_1570	U32 family peptidase
SAUSA300_0006	0,8	1,2	5,2E-03	Yes	8073	9136	7926	10566	9965	9855	11797	11362	10308	gyrA	DNA gyrase subunit A
SAUSA300_0658	0,8	1,2	3,2E-03	Yes	1405	1416	1538	1755	1754	1763	1656	1699	1789	SAUSA300_0658	LysR family transcriptional regulator
SAUSA300_1475	0,8	1,2	9,8E-03	Yes	5145	6159	5327	6714	7271	6086	6224	6325	5891	accC	acetyl-CoA carboxylase biotin carboxylase subunit
SAUSA300_0656	0,8	1,2	4,0E-03	Yes	1097	1136	1159	1342	1422	1315	1244	1417	1173	SAUSA300_0656	hypothetical protein
SAUSA300_1257	0,8	1,2	9,2E-03	Yes	1878	1756	1759	1995	2146	2242	1936	1949	2053	msrR	peptide methionine sulfoxide reductase regulator MsrR
SAUSA300_1162	0,9	1,2	3,2E-04	Yes	7742	8402	7796	9641	9214	9290	9651	10103	9616	infB	translation initiation factor IF-2
SAUSA300_1086	1,2	0,9	4,3E-03	Yes	4144	3983	3845	3387	3424	3480	3102	3125	3224	SAUSA300_1086	hypothetical protein
SAUSA300_2358	1,2	0,9	1,6E-03	Yes	9750	10184	8947	8609	7814	8244	6859	7034	6850	SAUSA300_2358	ABC transporter permease
SAUSA300_2021	1,2	0,8	7,6E-05	Yes	2584	2737	2442	2245	2140	2113	2041	2082	2000	SAUSA300_2021	S1 RNA-binding domain-containing protein
SAUSA300_1367	1,2	0,8	2,0E-03	Yes	1959	1977	1790	1495	1688	1541	1552	1547	1431	cmk	cytidylate kinase
SAUSA300_1038	1,2	0,8	4,4E-03	Yes	5284	5597	4931	4400	4339	4196	4410	4574	4372	pheT	phenylalanyl-tRNA synthetase subunit beta
SAUSA300_1108	1,2	0,8	9,5E-03	Yes	622	621	636	547	488	502	504	479	494	def	peptide deformylase
SAUSA300_0570	1,2	0,8	7,7E-03	Yes	7540	7895	6203	5760	6194	5609	5460	5419	4727	eutD	phosphotransacetylase
SAUSA300_1517	1,2	0,8	4,3E-05	Yes	2099	2033	2108	1735	1656	1677	1599	1651	1632	SAUSA300_1517	endonuclease IV
SAUSA300_1371	1,2	0,8	4,7E-03	Yes	1251	1349	1133	981	1072	961	935	913	938	recQ	ATP-dependent DNA helicase RecQ
SAUSA300_2329	1,2	0,8	4,7E-03	Yes	5198	4155	5305	3858	3895	4080	5028	5053	4930	gltT	proton/sodium-glutamate symport protein
SAUSA300_0032	1,2	0,8	6,0E-03	Yes	109024	96889	100585	85952	78206	82347	106258	111690	100287	mecA	penicillin-binding protein 2'
SAUSA300_2069	1,2	0,8	5,9E-03	Yes	613	694	601	493	553	488	509	539	484	SAUSA300_2069	hypothetical protein
SAUSA300_1285	1,2	0,8	6,0E-03	Yes	4264	4470	4233	3467	3546	3401	2983	2910	2802	SAUSA300_1285	ABC transporter ATP-binding protein
SAUSA300_0822	1,2	0,8	2,9E-04	Yes	8637	8928	7638	6825	7002	6388	6745	6458	6172	sufB	FeS assembly protein SufB
SAUSA300_1695	1,3	0,8	3,9E-03	Yes	6154	5783	5256	4101	4892	4720	4271	4303	4134	SAUSA300_1695	hypothetical protein
SAUSA300_1631	1,3	0,8	2,2E-03	Yes	1770	2067	1936	1475	1547	1559	1324	1524	1386	SAUSA300_1631	replication initiation and membrane attachment protein
SAUSA300_0462	1,3	0,8	2,1E-04	Yes	1282	1476	1359	1144	1093	1033	1289	1215	1181	SAUSA300_0462	hypothetical protein
SAUSA300_0563	1,3	0,8	5,2E-03	Yes	501	524	580	452	419	405	433	424	465	ung	uracil-DNA glycosylase
SAUSA300_1916	1,3	0,8	1,0E-03	Yes	6239	6695	5783	4634	5358	4767	4894	5202	4806	SAUSA300_1916	hypothetical protein
SAUSA300_1589	1,3	0,8	2,0E-04	Yes	1743	1754	1652	1211	1329	1503	1353	1333	1384	dtd	D-tyrosyl-tRNA(Tyr) deacylase
SAUSA300_1792	1,3	0,8	4,6E-04	Yes	9384	8652	8234	6708	6376	7502	7037	6976	7280	SAUSA300_1792	hypothetical protein
SAUSA300_2070	1,3	0,8	7,5E-04	Yes	930	1050	971	769	793	751	734	771	706	SAUSA300_2070	hypothetical protein
SAUSA300_0494	1,3	0,8	6,3E-04	Yes	1114	1217	1042	908	870	860	907	927	816	folK	2-amino-4-hydroxy-6- hydroxymethyldihydropteridine pyrophosphokinase
SAUSA300_1630	1,3	0,8	1,2E-03	Yes	1823	2192	1966	1579	1602	1493	1506	1499	1423	dnaI	primosomal protein DnaI

SAUSA300_1037	1,3	0,8	1,7E-03	Yes	1529	1689	1596	1371	1188	1171	1184	1287	1226	pheS	phenylalanyl-tRNA synthetase subunit alpha
SAUSA300_1036	1,3	0,8	2,7E-03	Yes	552	570	675	496	449	450	427	515	465	SAUSA300_1036	RNA methyltransferase
SAUSA300_1179	1,3	0,8	6,6E-03	Yes	22265	21589	17849	15018	17030	15534	17572	17450	16207	SAUSA300_1179	phosphodiesterase
SAUSA300_1103	1,3	0,8	5,5E-04	Yes	1469	1476	1736	1103	1292	1214	1185	1276	1253	rpoZ	DNA-directed RNA polymerase subunit omega
SAUSA300_0513	1,3	0,8	3,1E-05	Yes	7889	8352	7109	5952	6339	5711	6353	6122	5617	gltX	glutamyl-tRNA synthetase
SAUSA300_1701	1,3	0,8	6,4E-03	Yes	740	837	791	657	650	519	603	539	638	SAUSA300_1701	hypothetical protein
SAUSA300_2564	1,3	0,8	9,4E-03	Yes	357	475	358	283	318	311	298	274	300	estA	tributyrin esterase
SAUSA300_0981	1,3	0,8	9,4E-04	Yes	615	705	685	535	487	519	487	499	476	SAUSA300_0981	hypothetical protein
SAUSA300_1632	1,3	0,8	1,9E-03	Yes	814	934	848	642	718	631	627	703	639	nrdR	transcriptional regulator NrdR
SAUSA300_0001	1,3	0,8	1,1E-07	Yes	6252	5747	5571	4365	4419	4675	5167	5506	5274	dnaA	chromosomal replication initiation protein
SAUSA300_1858	1,3	0,8	2,3E-04	Yes	1031	1087	934	767	813	756	770	854	751	SAUSA300_1858	hypothetical protein
SAUSA300_1521	1,3	0,8	1,6E-04	Yes	6629	5797	5380	4548	4478	4535	4687	4570	4286	rpoD	RNA polymerase sigma factor RpoD
SAUSA300_2296	1,3	0,8	1,4E-03	Yes	2357	2563	2022	1709	1993	1564	1770	1791	1660	SAUSA300_2296	esterase-like protein
SAUSA300_1518	1,3	0,8	3,7E-08	Yes	3370	3279	3261	2611	2386	2517	2598	2339	2404	SAUSA300_1518	DEAD-box ATP dependent DNA helicase
SAUSA300_0702	1,3	0,8	7,2E-03	Yes	1604	2201	1544	1401	1374	1256	1302	1232	1245	SAUSA300_0702	hypothetical protein
SAUSA300_0952	1,3	0,8	4,0E-04	Yes	1915	1742	1742	1334	1407	1322	1431	1341	1364	SAUSA300_0952	aminotransferase, class I
SAUSA300_0467	1,3	0,8	4,7E-03	Yes	7213	9522	7531	6302	6419	5545	5465	5372	5421	metS	methionyl-tRNA synthetase
SAUSA300_2349	1,3	0,8	1,0E-03	Yes	699	761	833	526	648	551	664	665	639	SAUSA300_2349	formate/nitrite transporter family protein
SAUSA300_2282	1,3	0,7	1,5E-04	Yes	6192	5926	4895	3909	4326	4511	4477	4423	4968	SAUSA300_2282	hypothetical protein
SAUSA300_1356	1,3	0,7	7,4E-04	Yes	724	932	899	633	617	668	557	563	589	aroB	3-dehydroquinate synthase
SAUSA300_2176	1,3	0,7	8,5E-04	Yes	903	1051	923	762	728	665	766	666	690	cbiO	cobalt transporter ATP-binding subunit
SAUSA300_1260	1,3	0,7	4,9E-04	Yes	951	923	811	654	652	692	614	656	644	SAUSA300_1260	prephenate dehydrogenase
SAUSA300_1102	1,3	0,7	4,4E-05	Yes	3808	4171	4416	3079	3262	2897	3012	3018	2719	gmk	guanylate kinase
SAUSA300_2490	1,3	0,7	9,2E-03	Yes	428	400	347	269	315	287	328	307	310	SAUSA300_2490	TetR family regulatory protein
SAUSA300_0538	1,3	0,7	6,8E-03	Yes	1164	1181	1070	825	885	819	1024	1443	948	SAUSA300_0538	NAD-dependent epimerase/dehydratase family protein
SAUSA300_2232	1,4	0,7	3,3E-05	Yes	501	570	545	370	407	418	460	448	455	SAUSA300_2232	acetyltransferase
SAUSA300_0405	1,4	0,7	9,9E-03	Yes	1419	2085	1819	1274	1489	1179	1173	1251	1109	hsdM	type I restriction-modification system, M subunit
SAUSA300_2315	1,4	0,7	8,4E-03	Yes	2165	1855	1633	1295	1501	1368	1556	1439	1576	SAUSA300_2315	hypothetical protein
SAUSA300_2073	1,4	0,7	1,2E-03	Yes	2099	1832	1716	1347	1332	1477	1776	1698	1532	tdk	thymidine kinase
SAUSA300_1900	1,4	0,7	2,6E-06	Yes	9246	9303	8756	6709	6826	6541	6097	6387	5900	ppaC	putative manganese-dependent inorganic pyrophosphatase
SAUSA300_0463	1,4	0,7	3,2E-04	Yes	568	673	647	483	442	465	565	543	481	SAUSA300_0463	DNA replication intiation control protein YabA
SAUSA300_1293	1,4	0,7	1,4E-03	Yes	1219	1389	1287	869	1049	939	878	828	985	lysA	diaminopimelate decarboxylase
SAUSA300_0486	1,4	0,7	3,1E-04	Yes	4776	4406	4154	3029	3620	3119	3886	4125	3122	SAUSA300_0486	hypothetical protein
SAUSA300_0863	1,4	0,7	3,3E-03	Yes	562	606	553	455	356	452	401	442	427	argH	argininosuccinate lyase
SAUSA300_2044	1,4	0,7	2,7E-06	Yes	1985	2161	1773	1353	1430	1547	1327	1345	1352	cls	cardiolipin synthetase
SAUSA300_0676	1,4	0,7	2,6E-04	Yes	532	498	461	387	350	352	386	417	326	SAUSA300_0676	anion transporter family protein
SAUSA300_0028	1,4	0,7	1,3E-03	Yes	444	374	374	258	285	320	346	370	357	SAUSA300_0028	putative transposase
SAUSA300_0384	1,4	0,7	9,3E-04	Yes	749	681	610	501	489	494	492	534	574	SAUSA300_0384	hypothetical protein
SAUSA300_0677	1,4	0,7	1,6E-06	Yes	769	800	723	600	515	556	518	537	534	SAUSA300_0677	putative deoxyribodipyrimidine photolyase
SAUSA300_0916	1,4	0,7	1,4E-04	Yes	1609	1474	1291	931	1075	1165	1392	1519	1388	SAUSA300_0916	hypothetical protein
SAUSA300_0219	1,4	0,7	3,7E-03	Yes	347	367	306	257	261	222	199	254	206	SAUSA300_0219	putative iron compound A C transporter, iron compound-binding protein
SAUSA300_0897	1,4	0,7	4,6E-04	Yes	2362	3088	2828	2050	2132	1826	1780	1747	1691	trpS	tryptophanyl-tRNA synthetase
SAUSA300_2283	1,4	0,7	7,7E-05	Yes	652	817	728	472	584	531	485	518	493	rpiA	ribose-5-phosphate isomerase A
SAUSA300_1588	1,4	0,7	1,5E-04	Yes	4753	4669	3778	2878	3447	3207	3589	3350	3207	lytH	N-acetylmuramoyl-L-alanine amidase

SAUSA300_0544	1,4	0,7	2,3E-04	Yes	1527	1694	1256	1064	1112	1054	975	918	952	SAUSA300_0544	HAD superfamily hydrolase
SAUSA300_1629	1,4	0,7	5,3E-03	Yes	8905	9565	7550	5875	6701	6168	6588	6651	6125	thrS	threonyl-tRNA synthetase
SAUSA300_2636	1,4	0,7	3,3E-03	Yes	362	292	271	226	227	211	252	279	242	SAUSA300_2636	integrase/recombinase
SAUSA300_1896	1,4	0,7	2,9E-04	Yes	856	784	860	586	578	635	545	556	578	pheA	prephenate dehydratase
SAUSA300_0009	1,4	0,7	7,1E-05	Yes	3970	3900	3520	2604	2962	2615	3303	3656	2928	serS	seryl-tRNA synthetase
SAUSA300_1801	1,4	0,7	5,9E-04	Yes	1080	1214	999	777	809	779	842	1026	818	fumC	fumarate hydratase
SAUSA300_1590	1,4	0,7	3,9E-04	Yes	9246	11280	8792	6450	7788	6826	7077	7448	6604	SAUSA300_1590	GTP pyrophosphokinase
SAUSA300_2086	1,4	0,7	9,5E-03	Yes	1139	1537	1478	944	1166	872	1207	1269	1111	SAUSA300_2086	hypothetical protein
SAUSA300_2388	1,4	0,7	3,9E-03	Yes	1184	1431	1315	927	987	904	1129	1309	1291	panE	2-dehydropantoate 2-reductase
SAUSA300_2072	1,4	0,7	1,5E-06	Yes	3467	3145	3004	2338	2150	2396	2671	2744	2571	prfA	peptide chain release factor 1
SAUSA300_0184	1,4	0,7	5,6E-03	Yes	302	303	235	205	214	181	216	240	213	argB	acetylglutamate kinase
SAUSA300_0107	1,4	0,7	1,1E-03	Yes	6289	7602	6220	4827	5066	4503	4778	5273	4545	SAUSA300_0107	Na/Pi cotransporter family protein
SAUSA300_2045	1,4	0,7	3,3E-03	Yes	789	768	669	522	492	576	520	458	457	SAUSA300_2045	HD domain-containing protein
SAUSA300_2489	1,4	0,7	2,1E-03	Yes	361	381	276	254	240	232	267	242	248	SAUSA300_2489	antibiotic transport-associated protein-like protein
SAUSA300_2338	1,4	0,7	1,2E-04	Yes	906	1149	894	647	796	659	657	717	742	SAUSA300_2338	sensor histidine kinase
SAUSA300_1195	1,4	0,7	9,3E-07	Yes	1000	892	914	620	697	678	728	780	813	miaA	tRNA delta(2)-isopentenylpyrophosphate transferase
SAUSA300_1136	1,4	0,7	4,7E-03	Yes	411	516	508	359	320	348	366	342	320	rbgA	ribosomal biogenesis GTPase
SAUSA300_1327	1,4	0,7	1,5E-04	Yes	3815	4648	3994	2728	3244	2884	3097	3773	3639	SAUSA300_1327	cell surface protein
SAUSA300_2397	1,4	0,7	4,6E-03	Yes	2644	2050	2095	1618	1446	1757	1894	1623	1593	SAUSA300_2397	putative transport protein
SAUSA300_1672	1,4	0,7	1,2E-11	Yes	2539	2714	2578	1900	1834	1831	1696	1820	1794	nagE	phosphotransferase system, N-acetylglucosamine-specific IIBC component
SAUSA300_1319	1,4	0,7	6,1E-04	Yes	1667	1713	1360	1038	1304	1014	1112	1089	1032	folA	dihydrofolate reductase
SAUSA300_1252	1,4	0,7	3,8E-04	Yes	3424	4112	3890	2691	2722	2687	2831	2767	2634	SAUSA300_1252	amino acid carrier protein
SAUSA300_1899	1,4	0,7	8,8E-05	Yes	3756	3797	3696	2613	2773	2551	2782	2865	2539	SAUSA300_1899	hypothetical protein
SAUSA300_1698	1,4	0,7	1,6E-03	Yes	1532	1215	1510	1052	896	1054	1084	922	956	SAUSA300_1698	hypothetical protein
SAUSA300_2389	1,4	0,7	2,7E-04	Yes	5009	5150	5379	3454	3496	3995	4476	4772	4581	SAUSA300_2389	putative drug transporter
SAUSA300_2577	1,4	0,7	1,6E-05	Yes	787	800	694	520	527	553	545	635	519	manA	mannose-6-phosphate isomerase
SAUSA300_2337	1,4	0,7	2,6E-05	Yes	846	879	663	525	572	573	622	605	594	SAUSA300_2337	DegU family transcriptional regulator
SAUSA300_2225	1,4	0,7	7,0E-04	Yes	1052	1008	860	666	728	650	723	662	672	moaC	molybdenum cofactor biosynthesis protein MoaC
SAUSA300_0630	1,4	0,7	1,9E-03	Yes	4591	5115	5210	3253	3051	4108	3689	3396	5001	SAUSA300_0630	ABC transporter ATP-binding protein
SAUSA300_1591	1,4	0,7	8,0E-05	Yes	1509	1467	1379	985	1003	1043	1028	1083	974	apt	adenine phosphoribosyltransferase
SAUSA300_0204	1,4	0,7	5,4E-07	Yes	669	739	621	473	481	457	572	582	567	ggt	gamma-glutamyltranspeptidase
SAUSA300_2071	1,4	0,7	1,6E-05	Yes	2925	2629	2554	1909	1755	1955	2138	2164	2154	SAUSA300_2071	HemK family modification methylase
SAUSA300_0258	1,4	0,7	4,7E-04	Yes	1335	1042	1108	750	757	904	1129	1194	1137	SAUSA300_0258	GntR family transcriptional regulator
SAUSA300_2080	1,5	0,7	2,1E-03	Yes	937	677	850	518	526	653	606	566	581	SAUSA300_2080	hypothetical protein
SAUSA300_2576	1,5	0,7	3,2E-03	Yes	1187	1283	1128	704	980	789	859	970	729	SAUSA300_2576	phosphotransferase system, fructose-specific IIABC component
SAUSA300_0147	1,5	0,7	6,1E-03	Yes	250	344	287	237	209	163	199	208	182	SAUSA300_0147	5' nucleotidase family protein
SAUSA300_2407	1,5	0,7	5,6E-03	Yes	140	191	144	95	112	116	149	139	143	SAUSA300_2407	oligopeptide ABC transporter ATP-binding protein
SAUSA300_1137	1,5	0,7	4,0E-04	Yes	338	415	406	287	239	271	285	231	267	rnhB	ribonuclease HII
SAUSA300_1807	1,5	0,7	1,1E-04	Yes	331	294	259	184	202	211	184	189	182	SAUSA300_1807	amino acid ABC transporter ATP-binding protein
SAUSA300_2265	1,5	0,7	1,3E-03	Yes	1517	1945	2242	1313	1307	1272	1451	1604	1372	SAUSA300_2265	putative amino acid permease
SAUSA300_2297	1,5	0,7	2,2E-05	Yes	1108	876	862	623	617	692	656	566	641	SAUSA300_2297	hypothetical protein
SAUSA300_0596	1,5	0,7	4,3E-08	Yes	4350	4183	4118	2820	2828	2962	2796	2656	2849	argS	arginyl-tRNA synthetase
SAUSA300_1664	1,5	0,7	2,8E-07	Yes	9441	8133	9196	5665	5733	6822	5907	6461	6242	ezrA	septation ring formation regulator EzrA
SAUSA300_0543	1,5	0,7	2,2E-08	Yes	720	724	644	476	468	472	481	481	465	SAUSA300_0543	putative deaminase

SAUSA300_2255	1,5	0,7	6,0E-08	Yes	1402	1565	1314	980	975	950	938	918	858	SAUSA300_2255	hypothetical protein
SAUSA300_2288	1,5	0,7	3,1E-07	Yes	884	944	810	594	631	562	691	733	742	SAUSA300_2288	ABC transporter ATP-binding protein
SAUSA300_2268	1,5	0,7	7,9E-04	Yes	302	324	285	203	214	198	166	223	189	SAUSA300_2268	sodium/bile acid symporter family protein
SAUSA300_1357	1,5	0,7	1,3E-04	Yes	873	1091	938	697	666	600	602	587	597	aroC	chorismate synthase
SAUSA300_0794	1,5	0,7	7,0E-03	Yes	198	169	189	134	109	132	136	105	108	SAUSA300_0794	TOPRIM domain-containing protein
SAUSA300_0306	1,5	0,7	1,6E-03	Yes	3055	2331	2755	1738	1566	2159	2681	2857	2698	brnQ	branched-chain amino acid transport system II carrier protein
SAUSA300_1657	1,5	0,7	1,4E-09	Yes	10299	9815	8970	6055	6889	6484	6885	7076	6323	ackA	acetate kinase
SAUSA300_1592	1,5	0,7	2,7E-07	Yes	3022	2571	2464	1729	1648	2000	1875	1816	1782	recJ	single-stranded-DNA-specific exonuclease RecJ
SAUSA300_2384	1,5	0,7	5,1E-04	Yes	3942	3440	3487	2262	2470	2524	2390	2443	2512	SAUSA300_2384	putative Na+/H+ antiporter
SAUSA300_0187	1,5	0,7	6,0E-03	Yes	354	482	293	249	290	214	296	319	266	rocD	ornithine aminotransferase
SAUSA300_2541	1,5	0,7	8,8E-07	Yes	46928	49642	42647	28910	33306	30669	35085	37774	31253	mqo	malate:quinone oxidoreductase
SAUSA300_0582	1,5	0,7	9,4E-03	Yes	158	146	155	108	79	119	133	98	119	SAUSA300_0582	hypothetical protein
SAUSA300_1799	1,5	0,7	3,8E-07	Yes	1560	1437	1203	955	936	893	736	668	711	SAUSA300_1799	putative sensor histidine kinase
SAUSA300_1077	1,5	0,7	1,5E-21	Yes	2724	2923	2879	1902	1885	1870	2792	2915	3002	murD	UDP-N-acetylmuramoyl-L-alanyl-D-glutamate synthetase
SAUSA300_1584	1,5	0,7	2,6E-08	Yes	3090	2879	2325	1720	1886	1883	1948	2097	1823	SAUSA300_1584	recombination factor protein RarA
SAUSA300_0340	1,5	0,7	3,3E-03	Yes	141	135	126	98	86	82	108	82	121	SAUSA300_0340	NADH-dependent FMN reductase
SAUSA300_0862	1,5	0,7	1,9E-03	Yes	171	143	142	101	89	110	98	122	96	glpQ	glycerophosphoryl diester phosphodiesterase
SAUSA300_1318	1,5	0,7	5,4E-07	Yes	1479	1498	1332	972	924	943	886	855	888	SAUSA300_1318	hypothetical protein
SAUSA300_0012	1,5	0,7	3,7E-04	Yes	599	724	499	371	444	384	552	499	440	SAUSA300_0012	putative homoserine O-acetyltransferase
SAUSA300_0664	1,5	0,7	4,6E-05	Yes	2089	1468	1621	1054	1199	1137	1438	1448	1274	SAUSA300_0664	hypothetical protein
SAUSA300_2468	1,5	0,7	1,1E-03	Yes	378	475	355	215	319	255	337	320	292	SAUSA300_2468	acetyltransferase
SAUSA300_2324	1,5	0,7	7,7E-03	Yes	321	439	361	234	271	226	315	451	314	SAUSA300_2324	PTS system, sucrose-specific IIBC component
SAUSA300_0282	1,5	0,7	7,7E-03	Yes	632	454	360	331	296	312	773	491	631	SAUSA300_0282	hypothetical protein
SAUSA300_2303	1,5	0,7	2,8E-03	Yes	837	523	674	427	374	519	429	406	470	tcaR	transcriptional regulator TcaR
SAUSA300_2251	1,5	0,6	4,9E-03	Yes	2651	3225	2570	1731	2227	1517	2076	2027	1830	SAUSA300_2251	dehydrogenase family protein
SAUSA300_2250	1,5	0,6	2,0E-03	Yes	3123	3521	2614	1881	2325	1793	2497	2126	2044	nhaC	Na+/H+ antiporter NhaC
SAUSA300_0753	1,5	0,6	2,2E-06	Yes	1045	1053	873	642	728	552	589	635	533	SAUSA300_0753	hypothetical protein
SAUSA300_0132	1,5	0,6	3,5E-04	Yes	174	186	183	122	114	116	153	174	174	SAUSA300_0132	glycosyl transferase, group 1 family protein
SAUSA300_2517	1,5	0,6	9,1E-04	Yes	877	908	666	520	596	468	549	470	480	SAUSA300_2517	amidohydrolase family protein
SAUSA300_2095	1,6	0,6	4,8E-04	Yes	409	376	370	210	264	267	309	376	289	SAUSA300_2095	hypothetical protein
SAUSA300_1320	1,6	0,6	8,7E-06	Yes	1646	1706	1430	1076	969	1032	1021	930	914	thyA	thymidylate synthase
SAUSA300_1979	1,6	0,6	1,5E-03	Yes	352	280	312	183	202	221	188	164	203	SAUSA300_1979	cation transport family protein
SAUSA300_0668	1,6	0,6	9,7E-04	Yes	3764	3548	2844	1979	2653	1891	2496	2342	2012	SAUSA300_0668	hypothetical protein
SAUSA300_0254	1,6	0,6	4,0E-06	Yes	1264	1483	1304	904	921	774	822	910	885	SAUSA300_0254	sensor histidine kinase
SAUSA300_0038	1,6	0,6	9,1E-07	Yes	552	473	472	315	281	360	327	341	320	ccrA	cassette chromosome recombinase A
SAUSA300_0774	1,6	0,6	9,5E-03	Yes	127	94	92	62	66	71	63	79	58	empbp	secretory extracellular matrix and plasma binding protein
SAUSA300_2258	1,6	0,6	1,2E-05	Yes	8254	9387	9091	6245	5948	4907	7551	7785	7007	SAUSA300_2258	formate dehydrogenase, alpha subunit
SAUSA300_2356	1,6	0,6	2,9E-04	Yes	280	252	210	178	158	137	176	183	163	fmhA	fmhA protein
SAUSA300_2287	1,6	0,6	9,0E-05	Yes	1205	915	995	611	642	732	829	812	902	SAUSA300_2287	hypothetical protein
SAUSA300_0309	1,6	0,6	4,6E-05	Yes	1138	1033	974	608	778	618	885	1015	880	SAUSA300_0309	ABC transporter ATP-binding protein
SAUSA300_1229	1,6	0,6	2,2E-10	Yes	2406	2464	2462	1383	1621	1671	1632	1640	1595	SAUSA300_1229	HAD superfamily hydrolase
SAUSA300_1031	1,6	0,6	4,5E-03	Yes	120	143	137	72	80	102	77	73	98	SAUSA300_1031	hypothetical protein
SAUSA300_0864	1,6	0,6	2,3E-05	Yes	438	426	351	299	232	244	235	263	229	argG	argininosuccinate synthase
SAUSA300_0473	1,6	0,6	1,4E-15	Yes	2517	2472	2762	1728	1539	1672	1850	2033	1945	purR	pur operon repressor

SAUSA300_0146	1,6	0,6	8,8E-03	Yes	100	161	128	94	86	70	74	72	66	SAUSA300_0146	hypothetical protein
SAUSA300_0980	1,6	0,6	6,4E-16	Yes	4116	3538	3613	2344	2278	2537	2640	2764	2666	SAUSA300_0980	hypothetical protein
SAUSA300_1076	1,6	0,6	2,1E-09	Yes	951	999	1157	650	668	658	906	1035	940	mraY	phospho-N-acetylmuramoyl-pentapeptide- transferase
SAUSA300_2548	1,6	0,6	1,2E-05	Yes	242	212	212	134	142	143	227	207	233	SAUSA300_2548	hypothetical protein
SAUSA300_0977	1,6	0,6	7,4E-05	Yes	288	256	293	181	166	184	135	143	117	SAUSA300_0977	cobalt transport family protein
SAUSA300_1976	1,6	0,6	9,9E-03	Yes	762	1039	754	505	668	442	587	519	523	SAUSA300_1976	succinyl-diaminopimelate desuccinylase
SAUSA300_1295	1,6	0,6	8,0E-03	Yes	18096	14159	20650	7180	12349	13765	17281	15960	13319	SAUSA300_1295	CSD family cold shock protein
SAUSA300_0196	1,6	0,6	1,5E-05	Yes	3561	4059	3043	2027	2574	2107	2735	2902	2754	hsdR	type I restriction-modification enzyme, R subunit
SAUSA300_1499	1,6	0,6	1,2E-03	Yes	255	225	321	129	195	179	161	176	185	aroK	shikimate kinase
SAUSA300_1030	1,6	0,6	5,2E-03	Yes	94	157	131	78	84	78	76	71	80	SAUSA300_1030	iron transport associated domain-containing protein
SAUSA300_2385	1,6	0,6	2,9E-06	Yes	3253	3497	3131	2081	2054	2063	1964	2016	2212	SAUSA300_2385	hypothetical protein
SAUSA300_1284	1,6	0,6	1,2E-06	Yes	4920	3685	3902	2293	2653	2882	3188	3231	2816	SAUSA300_1284	hypothetical protein
SAUSA300_1870	1,6	0,6	5,1E-06	Yes	422	440	397	252	248	288	225	228	202	SAUSA300_1870	hypothetical protein
SAUSA300_0356	1,6	0,6	1,5E-09	Yes	556	575	518	335	380	317	407	419	359	SAUSA300_0356	hypothetical protein
SAUSA300_2590	1,6	0,6	1,5E-04	Yes	816	757	523	421	470	417	546	521	484	SAUSA300_2590	hypothetical protein
SAUSA300_0814	1,6	0,6	2,6E-03	Yes	6423	3747	4336	2786	2628	3637	4355	3518	3424	SAUSA300_0814	hypothetical protein
SAUSA300_0255	1,6	0,6	7,2E-12	Yes	630	674	648	418	384	417	475	456	465	SAUSA300_0255	two-component response regulator
SAUSA300_2447	1,6	0,6	7,1E-03	Yes	2584	2030	2473	1166	1778	1462	3184	2913	2516	SAUSA300_2447	hypothetical protein
SAUSA300_2411	1,6	0,6	1,8E-06	Yes	367	513	478	276	296	273	316	382	364	opp-1A	oligopeptide permease, peptide-binding protein
SAUSA300_0186	1,6	0,6	4,7E-04	Yes	354	438	294	230	251	191	301	280	254	argC	N-acetyl-gamma-glutamyl-phosphate reductase
SAUSA300_1048	1,6	0,6	8,8E-07	Yes	1439	1447	1332	923	858	824	1054	1316	956	sdhB	succinate dehydrogenase iron-sulfur subunit
SAUSA300_0817	1,6	0,6	1,2E-04	Yes	2677	2534	2324	1398	1748	1494	1741	1684	1349	SAUSA300_0817	hypothetical protein
SAUSA300_2591	1,6	0,6	4,2E-03	Yes	160	146	111	96	64	96	113	94	111	SAUSA300_2591	hypothetical protein
SAUSA300_2096	1,6	0,6	8,4E-12	Yes	1884	1756	1795	1094	1090	1161	1417	1502	1444	manA	mannose-6-phosphate isomerase
SAUSA300_2025	1,6	0,6	2,6E-15	Yes	1853	1980	1903	1142	1129	1257	1248	1170	1287	rsbU	sigma-B regulation protein
SAUSA300_0341	1,6	0,6	1,6E-10	Yes	807	921	904	499	563	555	538	560	570	SAUSA300_0341	hypothetical protein
SAUSA300_1788	1,6	0,6	2,1E-04	Yes	3547	2996	2630	1632	2261	1732	2100	2117	1796	SAUSA300_1788	hypothetical protein
SAUSA300_0185	1,6	0,6	1,5E-03	Yes	589	753	508	386	419	330	497	453	414	argJ	bifunctional ornithine acetyltransferase/N-acetylglutamate synthase protein
SAUSA300_2340	1,6	0,6	1,3E-04	Yes	150	170	159	87	99	106	91	86	102	narI	respiratory nitrate reductase, gamma subunit
SAUSA300_0567	1,6	0,6	2,2E-05	Yes	707	781	614	424	498	361	450	502	443	SAUSA300_0567	hypothetical protein
SAUSA300_1117	1,6	0,6	5,0E-03	Yes	19221	20589	25218	10938	14755	13993	20064	13440	10391	rpmB	50S ribosomal protein L28
SAUSA300_2627	1,6	0,6	7,7E-10	Yes	2489	2341	1919	1300	1492	1307	1691	1876	1532	SAUSA300_2627	2-oxoglutarate/malate translocator
SAUSA300_0382	1,6	0,6	4,3E-05	Yes	2972	3175	1991	1702	1692	1548	1371	1237	1269	SAUSA300_0382	sodium:dicarboxylate symporter family protein
SAUSA300_2593	1,6	0,6	2,5E-04	Yes	582	613	454	340	389	272	353	392	354	SAUSA300_2593	hypothetical protein
SAUSA300_0940	1,6	0,6	4,9E-04	Yes	2249	2078	2374	907	1535	1616	1715	1625	1368	SAUSA300_0940	hypothetical protein
SAUSA300_2449	1,7	0,6	8,0E-04	Yes	466	648	553	333	390	288	413	573	351	SAUSA300_2449	putative transporter
SAUSA300_2339	1,7	0,6	2,1E-08	Yes	626	699	533	401	388	336	376	392	366	SAUSA300_2339	hypothetical protein
SAUSA300_0308	1,7	0,6	2,0E-07	Yes	1097	1095	1080	640	694	640	888	977	749	SAUSA300_0308	ABC transporter permease
SAUSA300_0782	1,7	0,6	4,5E-03	Yes	1790	1172	1265	603	1014	922	1039	1074	820	SAUSA300_0782	hypothetical protein
SAUSA300_0406	1,7	0,6	8,3E-09	Yes	810	936	839	496	591	466	567	500	523	SAUSA300_0406	putative restriction/modification system specificity protein
SAUSA300_1856	1,7	0,6	2,1E-03	Yes	4335	4269	2992	1930	3025	1982	2548	2416	2142	SAUSA300_1856	hypothetical protein
SAUSA300_0847	1,7	0,6	3,1E-07	Yes	947	726	683	461	508	435	450	479	416	SAUSA300_0847	hypothetical protein
SAUSA300_0914	1,7	0,6	4,8E-07	Yes	392	493	409	233	295	242	299	330	267	SAUSA300_0914	sodium:alanine symporter family protein
SAUSA300_2460	1,7	0,6	1,8E-03	Yes	237	176	236	104	143	138	203	187	153	SAUSA300_2460	acetyltransferase family protein

SAUSA300_0472	1,7	0,6	1,9E-11	Yes	2494	2415	2635	1540	1287	1647	1771	1798	1814	ipk	4-diphosphocytidyl-2-C-methyl-D-erythritol kinase
SAUSA300_1621	1,7	0,6	5,2E-21	Yes	10809	10482	9653	5627	6256	6444	13888	13909	12597	clpX	ATP-dependent protease ATP-binding subunit ClpX
SAUSA300_0960	1,7	0,6	5,3E-08	Yes	6001	5899	5856	3412	3619	3482	3397	3796	3789	qoxD	quinol oxidase, subunit IV
SAUSA300_0568	1,7	0,6	1,1E-07	Yes	904	1088	829	555	617	497	527	508	490	SAUSA300_0568	hypothetical protein
SAUSA300_2600	1,7	0,6	9,2E-03	Yes	57	71	55	35	34	39	32	26	41	icaA	N-glycosyltransferase
SAUSA300_0471	1,7	0,6	2,7E-03	Yes	4718	3094	4969	1984	2170	3361	4179	3821	3974	SAUSA300_0471	hypothetical protein
SAUSA300_0721	1,7	0,6	8,4E-05	Yes	598	736	818	352	481	431	506	467	407	SAUSA300_0721	transferrin receptor
SAUSA300_1181	1,7	0,6	3,6E-17	Yes	1790	1787	1705	943	1045	1100	1140	1159	1097	SAUSA300_1181	hypothetical protein
SAUSA300_1809	1,7	0,6	4,8E-13	Yes	1108	1132	968	584	620	667	623	665	604	SAUSA300_1809	hypothetical protein
SAUSA300_1895	1,7	0,6	8,0E-07	Yes	954	788	891	558	456	524	542	545	567	SAUSA300_1895	nitric oxide synthase oxygenase
SAUSA300_0143	1,7	0,6	6,7E-04	Yes	77	95	96	54	56	47	56	47	45	phnE	phosphonate ABC transporter permease
SAUSA300_1453	1,7	0,6	8,4E-15	Yes	1175	1231	1061	663	672	682	638	686	665	SAUSA300_1453	ribonuclease Z
SAUSA300_0446	1,7	0,6	1,1E-03	Yes	337	571	528	322	295	221	216	210	201	gltD	glutamate synthase subunit beta
SAUSA300_0979	1,7	0,6	4,7E-04	Yes	659	739	571	366	456	315	388	348	277	SAUSA300_0979	hypothetical protein
SAUSA300_0279	1,7	0,6	1,2E-03	Yes	2909	1549	1418	1178	1053	1151	2800	2193	2776	SAUSA300_0279	hypothetical protein
SAUSA300_0566	1,7	0,6	7,6E-04	Yes	6375	6530	7050	3806	4385	3292	4504	4976	4221	SAUSA300_0566	amino acid permease
SAUSA300_2461	1,7	0,6	4,6E-05	Yes	680	502	460	321	320	299	408	393	318	SAUSA300_2461	glyoxalase family protein
SAUSA300_2257	1,7	0,6	4,1E-18	Yes	1574	1458	1597	929	895	833	1316	1196	1193	SAUSA300_2257	hypothetical protein
SAUSA300_0978	1,7	0,6	1,7E-06	Yes	1900	2314	1717	1148	1261	983	981	1052	817	SAUSA300_0978	ABC transporter ATP-binding protein
SAUSA300_0719	1,8	0,6	5,9E-03	Yes	80	127	126	55	63	71	50	61	75	SAUSA300_0719	iron compound ABC transporter permease
SAUSA300_1075	1,8	0,6	6,5E-34	Yes	8438	8318	8012	4695	4564	4713	8345	9054	8122	pbpA	penicillin-binding protein 1
SAUSA300_0929	1,8	0,6	4,0E-03	Yes	145	213	212	80	142	99	139	176	155	SAUSA300_0929	hypothetical protein
SAUSA300_0222	1,8	0,6	2,4E-12	Yes	1386	1360	1328	704	761	826	870	815	766	SAUSA300_0222	hypothetical protein
SAUSA300_0097	1,8	0,6	8,1E-15	Yes	705	858	777	450	432	433	603	580	604	SAUSA300_0097	hypothetical protein
SAUSA300_2439	1,8	0,6	2,0E-08	Yes	1202	1490	1114	691	805	632	866	894	796	galU	UTP-glucose-1-phosphate uridylyltransferase
SAUSA300_2326	1,8	0,6	4,9E-03	Yes	515	380	549	236	174	396	234	272	310	SAUSA300_2326	transcription regulatory protein
SAUSA300_2610	1,8	0,6	4,4E-03	Yes	66	87	85	52	44	38	41	61	50	hisC	histidinol-phosphate aminotransferase hisC
SAUSA300_0783	1,8	0,6	1,5E-10	Yes	800	765	634	366	405	446	454	447	471	SAUSA300_0783	phosphoglycerate mutase family protein
SAUSA300_1863	1,8	0,6	6,7E-07	Yes	2536	2196	2180	994	1538	1294	1606	1529	1247	SAUSA300_1863	hypothetical protein
SAUSA300_1677	1,8	0,6	3,2E-11	Yes	23213	22286	19407	11721	12365	11801	12765	13851	12913	SAUSA300_1677	cell wall surface anchor family protein
SAUSA300_0557	1,8	0,5	2,1E-07	Yes	446	509	396	252	266	225	223	224	205	SAUSA300_0557	HAD family hydrolase
SAUSA300_0961	1,8	0,5	2,1E-18	Yes	13490	13862	13684	7922	7230	7353	7288	8172	7633	qoxC	quinol oxidase, subunit III
SAUSA300_0618	1,8	0,5	3,3E-04	Yes	48689	44923	54013	28438	20597	31855	18512	20051	33398	SAUSA300_0618	ABC transporter substrate-binding protein
SAUSA300_0547	1,8	0,5	5,2E-03	Yes	4789	8201	9598	3575	4812	3983	5347	7825	7131	sdrD	sdrD protein
SAUSA300_0951	1,8	0,5	3,5E-08	Yes	675	636	505	387	336	272	263	268	238	sspA	V8 protease
SAUSA300_0619	1,8	0,5	5,0E-04	Yes	24929	25109	30672	15261	11143	17734	9406	10404	16985	SAUSA300_0619	ABC transporter permease
SAUSA300_2276	1,8	0,5	2,5E-04	Yes	158	292	228	119	140	112	171	160	156	SAUSA300_2276	M20/M25/M40 family peptidase
SAUSA300_2416	1,8	0,5	6,7E-03	Yes	287	348	367	168	220	158	214	187	197	SAUSA300_2416	glucose 1-dehydrogenase-like protein
SAUSA300_1074	1,8	0,5	5,3E-11	Yes	3872	3771	3663	1659	2420	2077	4141	4931	4115	ftsL	cell division protein
SAUSA300_0388	1,8	0,5	9,5E-07	Yes	26573	34919	30387	17651	17505	14911	15495	15681	14978	guaB	inosine-5'-monophosphate dehydrogenase
SAUSA300_0631	1,8	0,5	4,3E-26	Yes	3772	4111	4194	2103	2109	2338	2233	2347	2097	SAUSA300_0631	putative nucleoside transporter
SAUSA300_0145	1,8	0,5	4,2E-04	Yes	68	76	88	51	39	38	38	52	40	SAUSA300_0145	phosphonate ABC transporter phosphonate-binding protein
SAUSA300_1196	1,8	0,5	5,4E-11	Yes	665	554	496	304	280	340	444	438	393	hfq	RNA chaperone, host factor-1 protein
SAUSA300_0962	1,9	0,5	4,4E-17	Yes	44288	45484	44289	25172	24202	22934	24045	27252	25243	qoxB	quinol oxidase, subunit I

SAUSA300_0098	1,9	0,5	2,2E-07	Yes	257	244	299	124	139	168	221	193	201	SAUSA300_0098	hypothetical protein
SAUSA300_1855	1,9	0,5	1,1E-11	Yes	1122	923	884	553	492	531	526	498	512	sgtB	glycosyltransferase
SAUSA300_0445	1,9	0,5	8,0E-03	Yes	498	1111	900	541	460	351	357	302	278	gltB	glutamate synthase, large subunit
SAUSA300_1742	1,9	0,5	4,3E-05	Yes	121	101	98	51	57	62	77	91	89	SAUSA300_1742	hypothetical protein
SAUSA300_2396	1,9	0,5	3,8E-11	Yes	1085	1271	969	566	678	535	617	655	561	pnbA	para-nitrobenzyl esterase
SAUSA300_1800	1,9	0,5	3,5E-12	Yes	2991	2171	2266	1237	1391	1336	1592	1419	1345	SAUSA300_1800	ribosomal large subunit pseudouridine synthase D
SAUSA300_0247	1,9	0,5	1,1E-09	Yes	2041	2416	1883	955	1277	1147	1274	1286	1178	SAUSA300_0247	putative teichoic acid biosynthesis protein B
SAUSA300_1437	1,9	0,5	8,0E-04	Yes	888	633	954	388	312	618	418	387	453	SAUSA300_1437	phiSLT ORF204-like protein
SAUSA300_0389	1,9	0,5	1,2E-07	Yes	36302	42338	36877	20494	21873	18729	21809	22081	17994	guaA	GMP synthase
SAUSA300_2613	1,9	0,5	5,2E-03	Yes	67	65	52	26	38	32	25	43	41	hisZ	ATP phosphoribosyltransferase regulatory subunit
SAUSA300_2108	1,9	0,5	6,0E-13	Yes	1075	954	975	556	532	493	600	542	571	mtlD	mannitol-1-phosphate 5-dehydrogenase
SAUSA300_1671	1,9	0,5	1,2E-12	Yes	727	937	1042	471	479	475	522	511	586	SAUSA300_1671	hypothetical protein
SAUSA300_2026	1,9	0,5	4,4E-15	Yes	3639	4071	3477	1843	2240	1765	2304	2082	1900	SAUSA300_2026	PemK family protein
SAUSA300_2528	1,9	0,5	1,2E-11	Yes	583	541	525	245	298	308	328	325	301	SAUSA300_2528	hypothetical protein
SAUSA300_2399	1,9	0,5	1,4E-05	Yes	322	375	346	183	189	169	222	212	201	SAUSA300_2399	ABC transporter ATP-binding protein
SAUSA300_0602	1,9	0,5	4,7E-12	Yes	4489	3499	4363	1812	2137	2392	3275	3380	2742	SAUSA300_0602	hypothetical protein
SAUSA300_0246	1,9	0,5	1,1E-06	Yes	1050	1487	1241	524	763	652	664	676	625	SAUSA300_0246	putative alcohol dehydrogenase
SAUSA300_0506	1,9	0,5	2,4E-11	Yes	3709	3001	3637	1580	1599	2128	2071	2322	2096	nupC	pyrimidine nucleoside transport protein
SAUSA300_1264	2,0	0,5	3,6E-05	Yes	120	130	123	63	61	68	67	66	73	trpD	anthranilate phosphoribosyltransferase
SAUSA300_0203	2,0	0,5	8,3E-08	Yes	291	384	268	161	172	149	181	148	210	SAUSA300_0203	RGD-containing lipoprotein
SAUSA300_2403	2,0	0,5	1,8E-03	Yes	975	662	763	329	422	476	502	386	517	SAUSA300_2403	putative lipoprotein
SAUSA300_0934	2,0	0,5	1,2E-03	Yes	44	56	60	25	28	29	39	35	28	SAUSA300_0934	membrane protein
SAUSA300_1034	2,0	0,5	4,5E-03	Yes	83	77	99	37	35	60	37	38	55	srtB	sortase B
SAUSA300_2023	2,0	0,5	6,5E-08	Yes	2302	2250	2234	1085	1229	1140	1521	1408	1295	rsbW	serine-protein kinase RsbW
SAUSA300_2518	2,0	0,5	3,6E-06	Yes	2521	3273	2200	1365	1548	1135	1558	1427	1379	SAUSA300_2518	hydrolase family protein
SAUSA300_0344	2,0	0,5	4,9E-03	Yes	31	71	58	23	30	28	33	36	35	SAUSA300_0344	putative lipoprotein
SAUSA300_1057	2,0	0,5	8,1E-05	Yes	435	273	344	150	202	179	177	168	182	SAUSA300_1057	hypothetical protein
SAUSA300_0620	2,0	0,5	2,6E-05	Yes	40208	38843	44768	23445	16110	22979	13856	15008	23596	SAUSA300_0620	ABC transporter ATP-binding protein
SAUSA300_1035	2,0	0,5	3,7E-05	Yes	131	121	106	58	61	61	67	52	76	SAUSA300_1035	heme-degrading monooxygenase IsdG
SAUSA300_1708	2,0	0,5	1,1E-05	Yes	2942	2246	2805	1028	1388	1615	1678	1695	1242	rot	accessory regulator Rot
SAUSA300_0963	2,0	0,5	3,8E-14	Yes	25907	24072	24268	13664	11457	12168	11792	13739	13828	qoxA	quinol oxidase, subunit II
SAUSA300_2618	2,0	0,5	3,3E-04	Yes	60	85	86	32	45	39	37	51	59	SAUSA300_2618	hypothetical protein
SAUSA300_0718	2,0	0,5	2,3E-06	Yes	195	340	251	128	144	123	103	117	119	SAUSA300_0718	iron compound ABC transporter permease
SAUSA300_1804	2,0	0,5	1,9E-11	Yes	5328	3971	4502	2044	2575	2231	2504	2459	2311	SAUSA300_1804	hypothetical protein
SAUSA300_0374	2,0	0,5	2,0E-04	Yes	10080	10462	12249	4017	7092	5169	9512	9124	6864	SAUSA300_0374	hypothetical protein
SAUSA300_1684	2,0	0,5	1,4E-10	Yes	10453	9639	7499	4505	4877	4208	5041	4490	4392	SAUSA300_1684	hypothetical protein
SAUSA300_1267	2,0	0,5	1,1E-03	Yes	60	70	52	41	26	24	27	33	33	trpB	tryptophan synthase subunit beta
SAUSA300_1490	2,0	0,5	2,1E-12	Yes	11412	8776	9047	4277	5063	5043	5382	5134	4505	efp	elongation factor P
SAUSA300_2626	2,0	0,5	1,6E-04	Yes	264	188	201	90	129	99	232	173	137	SAUSA300_2626	hypothetical protein
SAUSA300_2436	2,0	0,5	4,7E-06	Yes	1045	1860	2040	881	909	634	865	876	759	SAUSA300_2436	putative cell wall surface anchor family protein
SAUSA300_2022	2,0	0,5	3,9E-08	Yes	9467	9084	7722	4194	4673	3962	5556	5369	4870	rpoF	RNA polymerase sigma factor SigB
SAUSA300_1061	2,1	0,5	8,0E-04	Yes	58	56	44	27	27	23	22	19	30	SAUSA300_1061	superantigen-like protein
SAUSA300_2107	2,1	0,5	9,2E-07	Yes	485	432	378	178	245	202	259	249	241	mtlA	PTS system, mannitol specific IIA component
SAUSA300_2629	2,1	0,5	2,7E-05	Yes	261	276	185	104	142	102	159	174	117	SAUSA300_2629	hypothetical protein

SAUSA300_0070	2,1	0,5	1,2E-06	Yes	1251	1041	965	460	657	455	709	626	562	SAUSA300_0070	putative lysophospholipase
SAUSA300_2592	2,1	0,5	2,7E-07	Yes	1167	842	814	421	487	447	619	621	499	SAUSA300_2592	hypothetical protein
SAUSA300_0387	2,1	0,5	4,0E-08	Yes	19352	23359	19954	10394	10570	9173	9439	9552	8971	pbuX	xanthine permease
SAUSA300_1971	2,1	0,5	9,7E-04	Yes	145	169	101	65	73	62	109	59	67	SAUSA300_1971	phi77 ORF017-like protein
SAUSA300_0245	2,1	0,5	5,6E-10	Yes	626	767	793	287	412	343	356	401	389	SAUSA300_0245	2-C-methyl-D-erythritol 4-phosphate cytidylyltransferase
SAUSA300_2145	2,1	0,5	1,5E-05	Yes	4287	4177	4080	1732	2399	1816	2498	2468	2091	SAUSA300_2145	glycine betaine transporter
SAUSA300_2252	2,1	0,5	5,8E-06	Yes	559	303	363	168	193	216	324	261	296	SAUSA300_2252	hypothetical protein
SAUSA300_0937	2,1	0,5	9,3E-03	Yes	280	238	456	83	151	225	270	199	215	SAUSA300_0937	hypothetical protein
SAUSA300_0913	2,1	0,5	3,0E-11	Yes	3283	2760	3776	1468	1333	1824	1459	1549	1741	SAUSA300_0913	hypothetical protein
SAUSA300_2379	2,1	0,5	5,9E-16	Yes	2337	1976	2173	878	1010	1161	1136	1246	1138	SAUSA300_2379	putative transporter protein
SAUSA300_0950	2,1	0,5	6,2E-13	Yes	666	521	525	317	241	248	192	219	223	sspB	cysteine protease
SAUSA300_1919	2,1	0,5	5,0E-04	Yes	3477	1709	1833	668	1030	1595	999	847	1055	SAUSA300_1919	hypothetical protein
SAUSA300_0711	2,2	0,5	3,0E-08	Yes	1550	1018	1064	526	597	563	755	759	593	SAUSA300_0711	hypothetical protein
SAUSA300_2572	2,2	0,5	1,2E-06	Yes	391	352	227	161	151	137	209	167	157	aur	zinc metalloproteinase aureolysin
SAUSA300_1974	2,2	0,5	1,7E-04	Yes	375	305	339	146	155	170	167	233	192	SAUSA300_1974	leukocidin/hemolysin toxin family protein
SAUSA300_2620	2,2	0,5	4,3E-05	Yes	904	660	579	271	322	398	350	304	421	SAUSA300_2620	hypothetical protein
SAUSA300_0712	2,2	0,5	5,5E-10	Yes	11679	14715	12954	5834	6867	5484	7420	8031	5777	SAUSA300_0712	amino acid/peptide transporter (peptide:H+ symporter)
SAUSA300_2398	2,2	0,5	2,3E-07	Yes	817	643	619	314	341	301	367	355	297	SAUSA300_2398	hypothetical protein
SAUSA300_2617	2,2	0,5	1,4E-14	Yes	341	437	366	168	201	156	209	219	234	SAUSA300_2617	putative cobalt ABC transporter ATP-binding protein
SAUSA300_1803	2,2	0,5	8,1E-06	Yes	1088	705	1056	388	353	560	444	373	430	SAUSA300_1803	hypothetical protein
SAUSA300_1740	2,2	0,5	3,8E-14	Yes	2248	2204	2165	1028	1026	941	1349	1989	1489	SAUSA300_1740	hypothetical protein
SAUSA300_1685	2,2	0,5	2,1E-09	Yes	16078	10927	11456	5003	6717	5660	8134	7103	5911	SAUSA300_1685	hypothetical protein
SAUSA300_0720	2,2	0,5	6,1E-08	Yes	108	152	140	58	63	60	71	66	65	SAUSA300_0720	putative iron compound ABC transporter ATP-binding protein
SAUSA300_1249	2,2	0,4	1,5E-13	Yes	2135	1527	1826	683	824	957	1080	1044	904	SAUSA300_1249	hypothetical protein
SAUSA300_0360	2,2	0,4	3,6E-04	Yes	150	215	106	56	91	63	67	78	65	SAUSA300_0360	Cys/Met metabolism PLP-dependent enzyme
SAUSA300_0273	2,2	0,4	1,5E-18	Yes	588	482	434	211	229	229	388	382	377	SAUSA300_0273	hypothetical protein
SAUSA300_1307	2,2	0,4	1,4E-11	Yes	1717	1559	1416	566	725	802	746	672	789	arlS	sensor histidine kinase protein
SAUSA300_0964	2,2	0,4	3,8E-14	Yes	4054	3051	2707	1362	1571	1437	1379	1147	1377	SAUSA300_0964	hypothetical protein
SAUSA300_1060	2,2	0,4	1,9E-03	Yes	40	48	56	15	23	26	26	31	29	SAUSA300_1060	superantigen-like protein
SAUSA300_1683	2,3	0,4	7,5E-29	Yes	3564	4310	4127	1706	1965	1661	1854	1828	2005	SAUSA300_1683	bifunctional 3-deoxy-7-phosphoheptulonate synthase/chorismate mutase
SAUSA300_2024	2,3	0,4	1,8E-15	Yes	1289	1248	1407	571	511	670	771	664	643	rsbV	anti-sigma-B factor, antagonist
SAUSA300_2619	2,3	0,4	3,3E-04	Yes	53	105	84	37	40	31	37	34	48	SAUSA300_2619	hypothetical protein
SAUSA300_0386	2,3	0,4	1,1E-09	Yes	6931	8579	7002	3458	3500	3008	3188	3062	2790	xpt	xanthine phosphoribosyltransferase
SAUSA300_0736	2,3	0,4	1,2E-10	Yes	2671	2670	2138	815	1314	1175	1580	1944	1703	yfiA	ribosomal subunit interface protein
SAUSA300_0201	2,3	0,4	3,6E-13	Yes	185	217	187	90	93	78	96	84	99	SAUSA300_0201	peptide ABC transporter permease
SAUSA300_2435	2,3	0,4	1,6E-09	Yes	1192	1863	1724	723	784	593	738	812	687	SAUSA300_2435	cell wall surface anchor family protein
SAUSA300_0359	2,3	0,4	3,8E-06	Yes	181	312	153	94	100	89	92	90	84	SAUSA300_0359	trans-sulfuration enzyme family protein
SAUSA300_1073	2,3	0,4	4,2E-20	Yes	11901	11564	10418	4194	5864	4743	12170	14178	11323	mraW	S-adenosyl-methyltransferase MraW
SAUSA300_0025	2,3	0,4	7,5E-12	Yes	1038	1486	1058	497	630	437	527	511	459	SAUSA300_0025	5'-nucleotidase family protein
SAUSA300_0815	2,3	0,4	1,5E-05	Yes	596	321	261	159	194	158	117	98	115	ear	Ear protein
SAUSA300_0846	2,3	0,4	3,2E-08	Yes	1068	648	708	382	323	348	227	220	238	SAUSA300_0846	Na+/H+ antiporter family protein
SAUSA300_0982	2,3	0,4	7,4E-11	Yes	1125	1023	1039	421	493	469	582	518	383	SAUSA300_0982	hypothetical protein
SAUSA300_2544	2,3	0,4	6,8E-03	Yes	48	47	69	19	20	32	46	20	27	SAUSA300_2544	hypothetical protein
SAUSA300_0354	2,3	0,4	1,3E-07	Yes	763	559	829	276	241	414	279	329	354	ltrA	hypothetical protein

SAUSA300_0337	2,3	0,4	4,7E-13	Yes	495	470	444	182	217	203	331	374	277	glpT	glycerol-3-phosphate transporter
SAUSA300_1210	2,3	0,4	9,7E-06	Yes	98	100	127	38	45	55	46	33	51	SAUSA300_1210	hypothetical protein
SAUSA300_2275	2,3	0,4	8,7E-08	Yes	1443	1820	1767	728	824	591	893	851	787	SAUSA300_2275	short chain dehydrogenase/reductase family oxidoreductase
SAUSA300_1263	2,4	0,4	1,5E-05	Yes	74	63	55	29	25	27	51	48	45	trpG	anthranilate synthase component II
SAUSA300_1739	2,4	0,4	8,1E-17	Yes	1627	1538	1656	756	637	657	938	1307	1056	SAUSA300_1739	hypothetical protein
SAUSA300_2615	2,4	0,4	3,8E-07	Yes	100	109	89	42	41	42	57	52	57	SAUSA300_2615	hypothetical protein
SAUSA300_0272	2,4	0,4	2,4E-09	Yes	635	367	414	220	171	206	345	334	345	SAUSA300_0272	hypothetical protein
SAUSA300_1986	2,4	0,4	5,7E-06	Yes	566	383	421	200	177	199	210	169	231	SAUSA300_1986	hypothetical protein
SAUSA300_1862	2,4	0,4	3,3E-14	Yes	4261	3473	3573	1332	1751	1658	1915	1874	1827	SAUSA300_1862	hypothetical protein
SAUSA300_2014	2,4	0,4	6,4E-17	Yes	285	265	296	116	121	118	129	132	142	ilvA	threonine dehydratase
SAUSA300_2616	2,4	0,4	2,0E-07	Yes	151	164	170	52	63	86	72	66	80	SAUSA300_2616	cobalt transport family protein
SAUSA300_1581	2,4	0,4	1,3E-03	Yes	847	467	1163	225	288	518	424	297	513	SAUSA300_1581	hypothetical protein
SAUSA300_1308	2,4	0,4	1,4E-11	Yes	1167	1294	1067	364	628	465	502	513	496	arlR	DNA-binding response regulator
SAUSA300_1065	2,4	0,4	3,8E-21	Yes	1764	1673	1542	667	635	757	619	676	652	SAUSA300_1065	exfoliative toxin A
SAUSA300_0767	2,4	0,4	4,7E-06	Yes	402	264	231	98	119	151	249	214	258	SAUSA300_0767	hypothetical protein
SAUSA300_1669	2,5	0,4	3,3E-24	Yes	901	1107	791	356	424	354	396	382	385	SAUSA300_1669	aminotransferase, class V
SAUSA300_0257	2,5	0,4	5,4E-08	Yes	385	533	595	193	215	204	191	287	173	SAUSA300_0257	antiholin-like protein LrgB
SAUSA300_0271	2,5	0,4	3,0E-10	Yes	669	456	439	228	188	211	358	330	351	SAUSA300_0271	ABC transporter ATP-binding protein
SAUSA300_0598	2,5	0,4	1,6E-19	Yes	1512	1637	1317	510	716	553	733	654	658	SAUSA300_0598	putative iron compound ABC transporter iron compound-binding protein
SAUSA300_1072	2,5	0,4	6,8E-39	Yes	6564	5504	6003	2147	2373	2683	5883	6022	5218	mraZ	cell division protein MraZ
SAUSA300_1670	2,5	0,4	2,6E-28	Yes	1328	1573	1126	491	557	535	627	621	595	serA	D-3-phosphoglycerate dehydrogenase
SAUSA300_0112	2,5	0,4	8,3E-13	Yes	5375	6879	4941	2147	2491	2107	2683	2298	2382	lctP	L-lactate permease
SAUSA300_0274	2,6	0,4	1,6E-10	Yes	2959	1823	2822	1081	866	1035	1879	1960	1961	SAUSA300_0274	hypothetical protein
SAUSA300_0343	2,6	0,4	2,6E-07	Yes	365	653	442	187	242	144	223	201	238	SAUSA300_0343	acetyltransferase
SAUSA300_0202	2,6	0,4	1,4E-07	Yes	157	236	134	61	84	61	104	86	70	SAUSA300_0202	peptide ABC transporter permease
SAUSA300_0816	2,6	0,4	2,3E-07	Yes	12951	10553	11391	3152	6134	4281	6547	5870	5073	SAUSA300_0816	CsbD-like superfamily protein
SAUSA300_2364	2,6	0,4	2,1E-08	Yes	14489	10497	7132	3674	4936	3760	2784	2795	2624	sbi	IgG-binding protein SBI
SAUSA300_2264	2,6	0,4	8,5E-09	Yes	3203	2471	3433	943	888	1660	1193	1159	1589	SAUSA300_2264	RpiR family phosphosugar-binding transcriptional regulator
SAUSA300_1883	2,6	0,4	2,5E-18	Yes	2283	2584	2289	856	1016	850	1277	1363	1155	putP	high affinity proline permease
SAUSA300_0206	2,6	0,4	5,1E-08	Yes	820	608	582	249	227	286	237	214	262	SAUSA300_0206	azoreductase
SAUSA300_0769	2,7	0,4	1,0E-07	Yes	1955	1064	1075	484	513	545	1009	936	914	SAUSA300_0769	hypothetical protein
SAUSA300_1058	2,7	0,4	2,6E-10	Yes	839	971	732	306	317	333	337	490	585	SAUSA300_1058	alpha-hemolysin
SAUSA300_1975	2,7	0,4	2,1E-05	Yes	291	199	295	98	76	121	107	123	155	SAUSA300_1975	Aerolysin/leukocidin family protein
SAUSA300_1864	2,7	0,4	1,0E-21	Yes	8126	6365	5935	2256	2858	2458	3021	2919	2849	SAUSA300_1864	hypothetical protein
SAUSA300_0278	2,7	0,4	8,8E-07	Yes	19024	7242	6505	4059	3971	3969	11019	10333	10894	SAUSA300_0278	hypothetical protein
SAUSA300_1582	2,7	0,4	2,7E-07	Yes	1784	1319	1664	482	629	623	1022	844	628	SAUSA300_1582	hypothetical protein
SAUSA300_2486	2,8	0,4	3,8E-15	Yes	4404	3477	2952	1296	1508	1131	1745	1722	1594	SAUSA300_2486	putative ATP-dependent Clp proteinase
SAUSA300_0135	2,8	0,4	9,8E-14	Yes	1158	1200	937	301	485	404	428	449	400	SAUSA300_0135	Fe/Mn family superoxide dismutase
SAUSA300_0475	2,8	0,4	4,5E-17	Yes	6329	5604	4795	1929	2280	1799	2749	2710	2236	SAUSA300_0475	regulatory protein SpoVG
SAUSA300_0954	2,8	0,4	2,7E-14	Yes	906	832	723	247	325	304	362	398	399	SAUSA300_0954	MarR family transcriptional regulator
SAUSA300_1741	2,8	0,4	2,1E-03	Yes	30	18	21	7	7	10	21	21	13	SAUSA300_1741	putative lipoprotein
SAUSA300_1212	2,8	0,4	6,1E-03	Yes	19	19	18	4	6	9	8	8	9	SAUSA300_1212	hypothetical protein
SAUSA300_2012	2,8	0,4	1,3E-15	Yes	334	418	377	127	147	122	145	171	159	leuC	isopropylmalate isomerase large subunit
SAUSA300_0785	2,9	0,4	4,6E-06	Yes	869	530	934	188	262	367	362	316	451	SAUSA300_0785	acetyltransferase

SAUSA300_2327	2,9	0,3	4,3E-14	Yes	1309	844	943	312	368	381	511	444	417	SAUSA300_2327	hypothetical protein
SAUSA300_0784	2,9	0,3	1,3E-14	Yes	703	636	687	168	260	262	295	311	276	SAUSA300_0784	LysE/YggA family protein
SAUSA300_0099	2,9	0,3	3,3E-09	Yes	145	188	199	59	61	61	67	56	63	plc	1-phosphatidylinositol phosphodiesterase
SAUSA300_0474	2,9	0,3	4,2E-32	Yes	5724	4731	4883	1814	1675	1714	2275	2174	1909	SAUSA300_0474	putative endoribonuclease L-PSP
SAUSA300_2247	3,0	0,3	1,1E-09	Yes	178	160	147	47	47	70	55	54	53	SAUSA300_2247	hypothetical protein
SAUSA300_2142	3,0	0,3	2,2E-22	Yes	32084	30718	32365	9313	12271	10478	17327	16647	14583	asp23	alkaline shock protein 23
SAUSA300_1231	3,0	0,3	5,8E-32	Yes	8995	9726	7555	2745	3210	2893	3199	3180	3151	SAUSA300_1231	gamma-aminobutyrate permease
SAUSA300_1922	3,0	0,3	1,6E-17	Yes	412	324	300	87	132	126	119	128	111	sak	staphylokinase
SAUSA300_2013	3,0	0,3	6,4E-14	Yes	145	199	167	57	60	54	71	68	58	leuD	isopropylmalate isomerase small subunit
SAUSA300_0358	3,0	0,3	1,0E-17	Yes	610	815	479	213	234	184	249	232	208	SAUSA300_0358	bifunctional homocysteine S-methyltransferase/5,10-methylenetetrahydrofolate reductase protein
SAUSA300_0116	3,0	0,3	1,6E-17	Yes	409	540	444	128	183	147	201	215	193	sirB	iron compound ABC transporter permease SirB
SAUSA300_1029	3,0	0,3	1,5E-11	Yes	3210	3620	2620	921	1233	958	926	942	951	SAUSA300_1029	iron transport associated domain-containing protein
SAUSA300_2007	3,1	0,3	4,4E-15	Yes	451	608	453	169	199	128	170	175	173	ilvB	acetolactate synthase large subunit
SAUSA300_2418	3,1	0,3	1,7E-11	Yes	1052	820	1014	232	362	345	551	488	474	SAUSA300_2418	hypothetical protein
SAUSA300_0256	3,1	0,3	8,5E-06	Yes	147	106	212	36	43	71	55	82	46	SAUSA300_0256	murein hydrolase regulator LrgA
SAUSA300_2538	3,1	0,3	4,4E-11	Yes	1786	1478	1817	471	585	590	887	1207	819	SAUSA300_2538	amino acid permease family protein
SAUSA300_1226	3,1	0,3	3,0E-22	Yes	866	1249	1246	358	396	334	539	573	634	SAUSA300_1226	homoserine dehydrogenase
SAUSA300_0115	3,1	0,3	1,9E-11	Yes	1954	1685	1646	441	727	525	865	800	780	sirC	iron compound ABC transporter permease SirC
SAUSA300_0768	3,1	0,3	3,2E-10	Yes	1010	611	516	252	219	208	584	440	383	SAUSA300_0768	hypothetical protein
SAUSA300_1292	3,2	0,3	2,6E-39	Yes	446	476	484	151	151	142	171	186	202	alr2	alanine racemase
SAUSA300_1291	3,2	0,3	2,6E-38	Yes	496	510	500	139	165	165	207	201	208	SAUSA300_1291	hippurate hydrolase
SAUSA300_2011	3,2	0,3	1,9E-12	Yes	242	325	264	89	101	69	112	127	78	leuB	3-isopropylmalate dehydrogenase
SAUSA300_1227	3,3	0,3	3,3E-30	Yes	1268	1485	1399	438	440	393	617	705	681	thrC	threonine synthase
SAUSA300_0010	3,4	0,3	2,2E-19	Yes	542	712	531	162	219	146	220	246	204	SAUSA300_0010	hypothetical protein
SAUSA300_0114	3,4	0,3	2,6E-09	Yes	1389	974	1686	302	278	613	316	395	466	SAUSA300_0114	accessory regulator
SAUSA300_1228	3,4	0,3	4,8E-42	Yes	1341	1415	1296	388	405	396	594	617	665	thrB	homoserine kinase
SAUSA300_2144	3,4	0,3	7,4E-27	Yes	13719	13502	13268	3622	4680	3511	5771	5394	4998	SAUSA300_2144	hypothetical protein
SAUSA300_0357	3,4	0,3	2,4E-43	Yes	1082	1204	913	298	336	295	423	394	351	metE	5-methyltetrahydropteroyltriglutamate-- homocysteine S-methyltransferase
SAUSA300_1014	3,5	0,3	2,6E-37	Yes	8769	11786	8194	2626	2939	2722	3208	3298	3116	pyc	pyruvate carboxylase
SAUSA300_1989	3,5	0,3	1,3E-03	Yes	10784	9098	13517	2387	1164	5976	2929	2411	5807	agrB	accessory gene regulator protein B
SAUSA300_0928	3,6	0,3	8,3E-13	Yes	124	117	125	47	26	32	40	34	40	comK	competence transcription factor
SAUSA300_0011	3,6	0,3	1,3E-13	Yes	295	420	297	90	117	71	125	130	102	SAUSA300_0011	hypothetical protein
SAUSA300_1211	3,6	0,3	2,1E-12	Yes	452	373	327	89	112	113	106	93	92	SAUSA300_1211	hypothetical protein
SAUSA300_1290	3,7	0,3	1,4E-33	Yes	421	464	477	104	135	124	181	210	174	dapD	tetrahydrodipicolinate acetyltransferase
SAUSA300_1991	3,8	0,3	1,4E-05	Yes	20958	18541	23713	4321	2833	9395	5454	4933	10372	agrC	accessory gene regulator protein C
SAUSA300_0889	3,8	0,3	1,7E-10	Yes	304	590	605	140	148	103	202	226	212	oppD	oligopeptide ABC transporter ATP-binding protein
SAUSA300_2248	3,9	0,3	3,3E-13	Yes	392	357	431	90	66	145	97	88	103	SAUSA300_2248	AraC family transcriptional regulator
SAUSA300_2010	4,0	0,3	1,6E-28	Yes	386	508	420	109	124	99	155	170	134	leuA	2-isopropylmalate synthase
SAUSA300_1225	4,0	0,3	2,1E-16	Yes	359	285	280	68	61	102	121	123	154	SAUSA300_1225	aspartate kinase
SAUSA300_0117	4,0	0,3	5,4E-13	Yes	1393	2073	1723	310	539	448	527	575	606	sirA	iron compound ABC transporter iron compound-binding protein SirA
SAUSA300_1992	4,0	0,2	2,1E-09	Yes	17343	16244	17373	3407	2381	6862	4391	4037	8027	agrA	accessory gene regulator protein A
SAUSA300_0887	4,0	0,2	1,1E-13	Yes	191	249	416	65	75	72	91	124	117	oppB	oligopeptide ABC transporter permease
SAUSA300_2008	4,1	0,2	1,5E-05	Yes	31	37	35	7	10	8	9	6	9	ilvN	acetolactate synthase 1 regulatory subunit
SAUSA300_2143	4,1	0,2	1,4E-30	Yes	6033	6562	5853	1529	1712	1299	2291	2041	1807	SAUSA300_2143	hypothetical protein

SAUSA300_0435	4,1	0,2	7,8E-17	Yes	347	668	462	107	140	113	134	187	166	SAUSA300_0435	ABC transporter ATP-binding protein
SAUSA300_0888	4,1	0,2	7,9E-11	Yes	163	278	346	76	66	52	101	114	111	oppC	oligopeptide ABC transporter permease
SAUSA300_0437	4,2	0,2	2,0E-47	Yes	769	1117	909	226	233	215	352	349	348	SAUSA300_0437	NLPA lipoprotein
SAUSA300_0890	4,2	0,2	3,8E-15	Yes	508	927	893	206	194	153	282	350	330	oppF	oligopeptide ABC transporter ATP-binding protein
SAUSA300_0796	4,3	0,2	1,6E-21	Yes	1596	2711	1859	420	603	399	603	649	555	SAUSA300_0796	ABC transporter ATP-binding protein
SAUSA300_0188	4,4	0,2	3,5E-72	Yes	4874	4549	4310	938	1079	1098	1425	1543	1497	brnQ	branched-chain amino acid transport system II carrier protein
SAUSA300_2417	4,7	0,2	1,9E-53	Yes	2275	2500	2446	484	587	464	730	796	726	SAUSA300_2417	putative transporter
SAUSA300_0372	4,7	0,2	8,5E-12	Yes	7369	8893	5213	1191	2187	1154	2158	1616	1527	SAUSA300_0372	hypothetical protein
SAUSA300_1289	4,8	0,2	4,1E-27	Yes	522	662	579	119	119	128	169	206	208	dapB	dihydrodipicolinate reductase
SAUSA300_1288	4,9	0,2	8,3E-35	Yes	502	673	534	128	124	97	192	206	220	dapA	dihydrodipicolinate synthase
SAUSA300_1286	4,9	0,2	8,3E-27	Yes	365	539	430	79	110	81	152	176	178	SAUSA300_1286	aspartate kinase
SAUSA300_0672	5,0	0,2	5,8E-62	Yes	18706	12329	14822	3017	2979	3213	3546	3543	3408	SAUSA300_0672	MarR family transcriptional regulator
SAUSA300_0898	5,0	0,2	7,2E-33	Yes	22384	12395	15728	2884	3297	3948	6153	5962	5928	spxA	transcriptional regulator Spx
SAUSA300_2006	5,0	0,2	2,4E-15	Yes	355	562	404	89	95	79	145	150	118	ilvD	dihydroxy-acid dehydratase
SAUSA300_2331	5,1	0,2	8,3E-45	Yes	2916	1898	1982	460	444	440	984	872	887	SAUSA300_2331	MarR family transcriptional regulator
SAUSA300_2009	5,1	0,2	5,9E-26	Yes	244	309	258	59	61	40	86	77	70	ilvC	ketol-acid reductoisomerase
SAUSA300_0436	5,1	0,2	4,6E-20	Yes	171	363	251	51	56	47	78	84	96	SAUSA300_0436	ABC transporter permease
SAUSA300_0891	5,1	0,2	6,5E-23	Yes	2141	2883	2630	518	556	415	907	933	864	oppA	oligopeptide ABC transporter substrate-binding protein
SAUSA300_0797	5,3	0,2	2,6E-26	Yes	878	1483	962	195	257	174	333	339	269	SAUSA300_0797	ABC transporter permease
SAUSA300_0136	5,3	0,2	6,8E-56	Yes	3065	2621	2510	485	518	534	600	642	537	SAUSA300_0136	cell wall surface anchor family protein
SAUSA300_0320	5,4	0,2	4,2E-80	Yes	10299	10689	8443	1770	1978	1747	2029	1830	2273	SAUSA300_0320	triacylglycerol lipase
SAUSA300_0776	5,5	0,2	9,7E-24	Yes	3545	2381	1902	464	481	488	620	529	519	nuc	thermonuclease
SAUSA300_1287	5,7	0,2	2,2E-32	Yes	647	839	737	113	145	130	193	262	239	asd	aspartate semialdehyde dehydrogenase
SAUSA300_0798	6,0	0,2	6,8E-57	Yes	1533	1998	1498	261	310	264	442	378	366	SAUSA300_0798	ABC transporter substrate-binding protein
SAUSA300_1068	6,3	0,2	3,9E-03	Yes	740	1209	1371	94	28	405	202	88	871	SAUSA300_1068	anti protein
SAUSA300_1890	7,3	0,1	2,4E-40	Yes	907	912	729	132	113	105	122	112	117	SAUSA300_1890	staphopain A
SAUSA300_0307	8,6	0,1	1,9E-48	Yes	3946	3925	3075	360	507	409	490	444	445	SAUSA300_0307	5'-nucleotidase
SAUSA300_2313	9,5	0,1	3,3E-03	Yes	1629	1587	9401	401	440	486	548	3944	723	SAUSA300_2313	L-lactate permease
SAUSA300_0113	12,9	0,1	2,5E-08	Yes	239701	387719	273617	10205	38766	20712	11548	39875	16946	SAUSA300_0113	immunoglobulin G binding protein A
SAUSA300_2454	29,5	0,0	3,9E-71	Yes	498	562	657	18	18	22	18	13	12	SAUSA300_2454	membrane spanning protein
SAUSA300_2453	50,1	0,0	1,4E-72	Yes	1329	2605	1302	30	42	32	24	26	22	SAUSA300_2453	ABC transporter ATP-binding protein

Supplementary Table 2. The complete list of genes expressed significantly differentially between JE2ΔclpX and the $JE2_{clpXI265E}$ strains.

ID	ΔclpX/X_{I265E}	padj	WT-E1	WT-E2	WT-E3	clpX-E1	clpX-E2	clpX-E3	X_{I265E} 1	X_{I265E} 2	X_{I265E} 3	Product	Gene
SAUSA300_0807	5,0	4,6E-10	34	17	15	1414	694	707	154	207	201	SaPI gene, function unknown	SAUSA300_0807
SAUSA300_0806	5,0	2,5E-09	9	3	6	357	153	187	34	52	53	SaPI gene, function unknown	SAUSA300_0806
SAUSA300_0805	4,6	2,3E-05	70	23	34	2361	1181	1175	273	432	310	xis, SaPI excisionase	SAUSA300_0805
SAUSA300_0808	4,5	5,8E-11	19	8	3	493	262	361	61	93	96	SaPI gene, function unknown	SAUSA300_0808
SAUSA300_0809	4,4	4,6E-10	262	138	149	8170	3810	3861	996	1369	1216	SaPI5 DNA primase	SAUSA300_0809
SAUSA300_0811	4,0	1,8E-05	27	14	12	423	163	204	51	58	86	SaPI gene, function unknown	SAUSA300_0811
SAUSA300_0810	4,0	2,8E-11	68	59	55	1560	803	705	225	302	246	SaPI gene, function unknown	SAUSA300_0810
SAUSA300_0804	3,9	1,6E-10	44	16	26	1502	1025	966	239	365	284	putative transcriptional regulator	SAUSA300_0804
SAUSA300_0813	3,6	1,7E-10	66	64	82	974	433	522	155	197	180	SaPI gene, function unknown	SAUSA300_0813
SAUSA300_0812	3,6	1,1E-09	364	248	254	4372	2018	2339	687	891	859	SaPI5 pif	SAUSA300_0812
SAUSA300_0365	2,6	3,2E-04	106	48	58	378	273	283	108	157	100	hypothetical protein SAS009 63aa	SAUSA300_0365
SAUSA300_2051	2,0	5,7E-06	133	160	116	587	621	468	265	297	267	sceD, WalkR controlled peptidoglycan hydrolases	SAUSA300_2051
SAUSA300_1903	1,9	2,1E-03	245	194	225	651	408	453	251	280	266	hypothetical protein, LexA operon	SAUSA300_1903
SAUSA300_2493	1,9	6,3E-03	46	25	32	128	116	156	70	86	57	cwrA (gene responding to cell wall damage)	SAUSA300_2493
SAUSA300_0741	1,5	5,2E-03	1141	1395	1255	4205	2765	2602	2103	2220	2145	excinuclease ABC subunit B	uvrB
SAUSA300_0742	1,5	7,2E-03	2496	2733	2669	7854	4880	4933	3873	4258	3927	excinuclease ABC subunit A	uvrA
SAUSA300_1039	1,5	1,8E-05	369	412	381	740	731	635	495	490	461	ribonuclease HIII	rnhC
SAUSA300_1548	1,5	9,8E-04	676	717	614	782	1040	861	620	633	598	ComE operon protein 2	SAUSA300_1548
SAUSA300_1483	1,4	6,3E-03	114	136	121	153	143	138	95	100	106	hypothetical protein	SAUSA300_1483
SAUSA300_1104	1,3	8,4E-04	777	936	833	1296	1256	1052	943	899	855	phosphopantothenoylcysteine decarboxylase/	coaBC
SAUSA300_1156	1,3	8,4E-04	4502	4997	4957	6943	6279	5780	4700	5026	4672	prolyl-tRNA synthetase	proS
SAUSA300_1799	1,3	6,5E-03	1560	1437	1203	955	936	893	736	668	711	putative sensor histidine kinase	SAUSA300_1799
SAUSA300_1177	1,3	8,5E-03	458	586	519	954	1034	936	774	687	774	competence/damage-inducible protein cinA	cinA
SAUSA300_1301	1,3	7,6E-03	1947	2094	1875	2168	2214	2224	1712	1650	1749	hypothetical protein	SAUSA300_1301
SAUSA300_1043	1,3	2,4E-04	1497	1647	1468	2573	2300	2223	1939	1817	1817	recombination and DNA strand exchange inhibitor protein	mutS2
SAUSA300_2359	1,3	2,5E-03	9555	10389	9478	9608	8872	8427	7020	7063	7129	amino acid ABC transporter amino acid-binding protein	SAUSA300_2359
SAUSA300_1451	1,3	7,1E-03	418	500	426	657	647	630	483	488	553	short chain dehydrogenase/reductase family oxidoreductase	SAUSA300_1451
SAUSA300_2128	1,3	4,0E-03	429	418	399	510	467	488	399	387	380	putative drug transporter	SAUSA300_2128
SAUSA300_1570	1,2	6,6E-03	1758	1864	1709	2374	2074	2002	1792	1665	1706	U32 family peptidase	SAUSA300_1570
SAUSA300_1459	1,2	6,3E-04	10163	10742	10767	11779	12059	11798	10014	9981	9876	6-phosphogluconate dehydrogenase	gnd
SAUSA300_2358	1,2	3,3E-03	9750	10184	8947	8609	7814	8244	6859	7034	6850	ABC transporter permease	SAUSA300_2358
SAUSA300_0001	0,8	6,3E-03	6252	5747	5571	4365	4419	4675	5167	5506	5274	chromosomal replication initiation protein	dnaA
SAUSA300_2467	0,8	8,3E-03	1767	1504	1633	1729	1792	1890	2331	2266	2196	sortase	srtA
SAUSA300_2329	0,8	8,1E-03	5198	4155	5305	3858	3895	4080	5028	5053	4930	proton/sodium-glutamate symport protein	gltT
SAUSA300_0020	0,8	8,0E-04	1583	1590	1575	2356	2376	2289	3044	3111	2860	DNA-binding response regulator	SAUSA300_0020

SAUSA300_0032	0,8	6,5E-03	109024	96889	100585	85952	78206	82347	106258	111690	100287	penicillin-binding protein 2'	mecA
SAUSA300_2096	0,8	2,2E-03	1884	1756	1795	1094	1090	1161	1417	1502	1444	mannose-6-phosphate isomerase	manA
SAUSA300_2587	0,8	1,1E-03	906	991	950	1735	1567	1452	2031	1996	2232	accessory secretory protein Asp1	SAUSA300_2587
SAUSA300_0035	0,8	5,1E-03	3758	3348	3173	2697	2745	3135	3676	4332	3407	hypothetical protein	SAUSA300_0035
SAUSA300_2494	0,7	9,2E-03	2299	2477	1754	3728	3704	3577	5217	4629	4884	copper-translocating P-type ATPase	copA
SAUSA300_0916	0,7	2,0E-03	1609	1474	1291	931	1075	1165	1392	1519	1388	hypothetical protein	SAUSA300_0916
SAUSA300_0097	0,7	5,4E-04	705	858	777	450	432	433	603	580	604	hypothetical protein	SAUSA300_0097
SAUSA300_1196	0,7	6,0E-03	665	554	496	304	280	340	444	438	393	RNA chaperone, host factor-1 protein	hfq
SAUSA300_2257	0,7	5,2E-06	1574	1458	1597	929	895	833	1316	1196	1193	hypothetical protein	SAUSA300_2257
SAUSA300_1306	0,7	7,3E-04	1837	2297	1830	1616	1759	1586	2194	2620	2235	2-oxoglutarate dehydrogenase E1 component	sucA
SAUSA300_0078	0,7	1,4E-04	3294	3301	2688	6651	5995	6057	9391	8240	8972	ATPase copper transport	copX
SAUSA300_0385	0,7	6,3E-03	519	430	486	421	532	507	712	694	680	hypothetical protein	SAUSA300_0385
SAUSA300_0188	0,7	4,9E-04	4874	4549	4310	938	1079	1098	1425	1543	1497	branched-chain amino acid transport system II carrier protein	brnQ1
SAUSA300_0258	0,7	3,8E-03	1335	1042	1108	750	757	904	1129	1194	1137	GntR family transcriptional regulator	SAUSA300_0258
SAUSA300_1305	0,7	6,2E-06	977	1190	999	861	939	976	1310	1416	1323	dihydrolipoamide succinyltransferase	sucB
SAUSA300_1076	0,7	8,7E-06	951	999	1157	650	668	658	906	1035	940	phospho-N-acetylmuramoyl-pentapeptide- transferase	mraY
SAUSA300_2243	0,7	7,1E-03	342	371	353	2402	1626	1650	2642	2797	2849	urease accessory protein UreG	ureG
SAUSA300_2417	0,7	3,2E-03	2275	2500	2446	484	587	464	730	796	726	putative transporter	SAUSA300_2417
SAUSA300_0128	0,7	1,0E-03	251	185	203	224	225	191	317	330	292	hypothetical protein	SAUSA300_0128
SAUSA300_1237	0,7	7,4E-03	2975	2137	2363	2298	2399	3035	3801	4057	3511	LexA repressor	lexA
SAUSA300_2574	0,7	6,5E-03	538	571	455	417	442	350	583	651	547	hypothetical protein	SAUSA300_2574
SAUSA300_0079	0,7	5,6E-05	968	908	820	1995	1900	1833	2977	2576	2891	putative lipoprotein	SAUSA300_0079
SAUSA300_0602	0,7	7,3E-04	4489	3499	4363	1812	2137	2392	3275	3380	2742	hypothetical protein	SAUSA300_0602
SAUSA300_1078	0,7	3,6E-08	3580	4302	3697	3427	3449	3087	4858	4969	4933	cell division protein	divIB
SAUSA300_2240	0,7	9,2E-03	421	428	467	6229	4159	4263	7070	7683	7302	urease subunit alpha	ureC
SAUSA300_0306	0,7	6,5E-03	3055	2331	2755	1738	1566	2159	2681	2857	2698	branched-chain amino acid transport system II carrier protein	brnQ
SAUSA300_2523	0,7	8,5E-03	118	140	133	368	451	334	572	594	573	hypothetical protein	SAUSA300_2523
SAUSA300_2239	0,7	4,5E-03	94	93	95	1818	1199	1117	2028	2162	2058	urease subunit beta	ureB
SAUSA300_2142	0,7	3,3E-03	32084	30718	32365	9313	12271	10478	17327	16647	14583	alkaline shock protein 23	asp23
SAUSA300_2455	0,7	1,1E-03	255	230	201	274	281	310	395	530	389	putative fructose-1,6-bisphosphatase	SAUSA300_2455
SAUSA300_2241	0,7	6,5E-03	178	193	197	2358	1546	1570	2638	2792	2866	urease accessory protein UreE	ureE
SAUSA300_2390	0,7	6,0E-04	693	571	592	1139	1198	1328	2082	1568	1953	glycine betaine/carnitine/choline transport system permease	opuCd
SAUSA300_2244	0,7	5,5E-03	378	334	362	2320	1538	1932	2683	2858	3320	urease accessory protein UreD	ureD
SAUSA300_1077	0,6	1,6E-23	2724	2923	2879	1902	1885	1870	2792	2915	3002	UDP-N-acetylmuramoyl-L-alanyl-D-glutamate synthetase	murD
SAUSA300_1563	0,6	1,8E-03	110	152	143	158	172	137	242	257	223	acetyl-CoA carboxylase, biotin carboxylase	accC
SAUSA300_1290	0,6	1,7E-03	421	464	477	104	135	124	181	210	174	tetrahydrodipicolinate acetyltransferase	dapD

SAUSA300_1565	0,6	4,3E-03	68	95	94	75	93	66	117	132	114	hypothetical protein	SAUSA300_1565
SAUSA300_2524	0,6	9,4E-03	61	46	59	108	144	121	183	192	205	hypothetical protein	SAUSA300_2524
SAUSA300_2476	0,6	7,2E-03	923	1156	1138	1339	1412	1342	1948	2652	1756	phosphotransferase system, glucose-specific IIABC component	ptsG
SAUSA300_2648	0,6	5,7E-03	7360	4598	5594	5248	6656	7277	10994	9889	8963	50S ribosomal protein L34	rpmH
SAUSA300_0437	0,6	3,0E-04	769	1117	909	226	233	215	352	349	348	NLPA lipoprotein	SAUSA300_0437
SAUSA300_2589	0,6	5,7E-09	23639	20777	20326	30034	26358	30390	41032	46977	47655	SasA adjesom cell wall anchor domain-containing protein	SAUSA300_2589
SAUSA300_1227	0,6	3,3E-04	1268	1485	1399	438	440	393	617	705	681	threonine synthase	thrC
SAUSA300_1228	0,6	2,1E-05	1341	1415	1296	388	405	396	594	617	665	homoserine kinase	thrB
SAUSA300_0736	0,6	3,7E-03	2671	2670	2138	815	1314	1175	1580	1944	1703	ribosomal subunit interface protein	yfiA
SAUSA300_2548	0,6	4,8E-05	242	212	212	134	142	143	227	207	233	hypothetical protein	SAUSA300_2548
SAUSA300_1226	0,6	9,4E-04	866	1249	1246	358	396	334	539	573	634	homoserine dehydrogenase, hom	hom
SAUSA300_1739	0,6	8,0E-05	1627	1538	1656	756	637	657	938	1307	1056	hypothetical protein	SAUSA300_1739
SAUSA300_1740	0,6	1,1E-04	2248	2204	2165	1028	1026	941	1349	1989	1489	hypothetical protein, lipid anchored	SAUSA300_1740
SAUSA300_2596	0,6	1,9E-03	101	104	100	96	94	93	150	142	164	capsular polysaccharide biosynthesis protein Cap1C	cap1C
SAUSA300_0772	0,6	1,4E-03	12737	10353	10113	11687	9367	10868	16470	18328	16995	clumping factor A	clfA
SAUSA300_0337	0,6	5,3E-04	495	470	444	182	217	203	331	374	277	glycerol-3-phosphate transporter	glpT
SAUSA300_2238	0,6	5,9E-03	30	27	29	595	442	466	838	823	787	urease subunit gamma	ureA
SAUSA300_0286	0,6	4,6E-03	123	120	115	152	97	105	189	173	208	Esx E toxin-antitoxin system (type VII secretion locus)	SAUSA300_0286
SAUSA300_0271	0,6	5,2E-03	669	456	439	228	188	211	358	330	351	ABC transporter ATP-binding protein	SAUSA300_0271
SAUSA300_2289	0,6	4,5E-04	328	188	227	230	220	214	346	371	406	hypothetical protein	SAUSA300_2289
SAUSA300_2495	0,6	7,3E-05	501	364	385	635	801	805	1375	1259	1165	copper chaperone copZ	copZ
SAUSA300_0284	0,6	6,2E-03	98	70	69	80	64	56	117	92	129	EsxC toxin (type VII secretion locus)	SAUSA300_0284
SAUSA300_0273	0,6	6,7E-08	588	482	434	211	229	229	388	382	377	hypothetical protein	SAUSA300_0273
SAUSA300_2551	0,6	4,7E-03	451	415	433	408	424	395	692	660	752	anaerobic ribonucleoside triphosphate reductase	nrdD
SAUSA300_0272	0,6	2,1E-03	635	367	414	220	171	206	345	334	345	hypothetical protein	SAUSA300_0272
SAUSA300_2603	0,6	5,7E-03	248	259	199	234	172	254	380	304	468	triacylglycerol lipase	lip
SAUSA300_2597	0,6	1,4E-05	100	113	98	110	94	87	166	171	169	capsular polysaccharide biosynthesis protein Cap1B	cap1B
SAUSA300_2538	0,6	8,3E-03	1786	1478	1817	471	585	590	887	1207	819	amino acid permease family protein	SAUSA300_2538
SAUSA300_1288	0,6	3,2E-04	502	673	534	128	124	97	192	206	220	dihydrodipicolinate synthase	dapA
SAUSA300_0898	0,6	4,9E-04	22384	12395	15728	2884	3297	3948	6153	5962	5928	transcriptional regulator Spx	spxA
SAUSA300_1287	0,6	2,0E-03	647	839	737	113	145	130	193	262	239	aspartate semialdehyde dehydrogenase	asd
SAUSA300_0277	0,6	1,8E-04	1214	944	711	718	747	555	1250	1200	1152	putative staphyloxanthin biosynthesis protein	SAUSA300_0277
SAUSA300_2550	0,6	1,2E-03	101	109	124	119	115	128	210	202	244	anaerobic ribonucleotide reductase, small subunit	nrdG
SAUSA300_0558	0,6	1,6E-04	4246	2443	2797	4201	3409	4142	6739	7445	7106	putative proline/betaine transporter	SAUSA300_0558
SAUSA300_0891	0,6	5,1E-03	2141	2883	2630	518	556	415	907	933	864	oligopeptide ABC transporter substrate-binding protein	opp-3a
SAUSA300_1075	0,5	8,5E-37	8438	8318	8012	4695	4564	4713	8345	9054	8122	penicillin-binding protein 1	pbpA

**SAUSA300_0769**	**0,5**	**6,7E-03**	**1955**	**1064**	**1075**	**484**	**513**	**545**	**1009**	**936**	**914**	**hypothetical protein**	**SAUSA300_0769**
**SAUSA300_1286**	**0,5**	**7,9E-04**	**365**	**539**	**430**	**79**	**110**	**81**	**152**	**176**	**178**	**aspartate kinase**	**SAUSA300_1286**
**SAUSA300_0285**	**0,5**	**2,8E-03**	**125**	**101**	**131**	**126**	**84**	**75**	**202**	**153**	**178**	**EsxB toxin (type VII secretion locus)**	**SAUSA300_0285**
**SAUSA300_2614**	**0,5**	**5,2E-09**	**579**	**593**	**555**	**503**	**507**	**538**	**852**	**1078**	**993**	**hypothetical protein**	**SAUSA300_2614**
**SAUSA300_2237**	**0,5**	**1,4E-03**	**96**	**68**	**84**	**659**	**395**	**555**	**950**	**980**	**1146**	**putative urea transporter**	**SAUSA300_2237**
**SAUSA300_0287**	**0,5**	**2,8E-03**	**61**	**73**	**64**	**72**	**63**	**47**	**122**	**111**	**114**	**EsxD toxin (type VII secretion locus)**	**SAUSA300_0287**
**SAUSA300_0274**	**0,5**	**9,9E-05**	**2959**	**1823**	**2822**	**1081**	**866**	**1035**	**1879**	**1960**	**1961**	**hypothetical protein**	**SAUSA300_0274**
**SAUSA300_0283**	**0,5**	**5,4E-04**	**2711**	**1693**	**1327**	**1561**	**1252**	**1128**	**2952**	**2137**	**2609**	**EssC protein (membrane- bound, type VII secretion**	**essC**
**SAUSA300_2447**	**0,5**	**7,9E-04**	**2584**	**2030**	**2473**	**1166**	**1778**	**1462**	**3184**	**2913**	**2516**	**hypothetical protein**	**SAUSA300_2447**
**SAUSA300_0767**	**0,5**	**4,6E-03**	**402**	**264**	**231**	**98**	**119**	**151**	**249**	**214**	**258**	**hypothetical protein**	**SAUSA300_0767**
**SAUSA300_0282**	**0,5**	**5,2E-05**	**632**	**454**	**360**	**331**	**296**	**312**	**773**	**491**	**631**	**EssB protein (membrane- bound, type VII secretion)**	**essB**
**SAUSA300_2331**	**0,5**	**3,6E-08**	**2916**	**1898**	**1982**	**460**	**444**	**440**	**984**	**872**	**887**	**sarZ MarR family transcriptional regulator**	**sarZ**
**SAUSA300_0768**	**0,5**	**8,5E-04**	**1010**	**611**	**516**	**252**	**219**	**208**	**584**	**440**	**383**	**hypothetical protein**	**SAUSA300_0768**
**SAUSA300_0280**	**0,5**	**8,4E-04**	**347**	**192**	**194**	**155**	**121**	**162**	**337**	**256**	**338**	**EssA (membrane- bound, type VII secretion)**	**ess**
**SAUSA300_1074**	**0,5**	**1,2E-15**	**3872**	**3771**	**3663**	**1659**	**2420**	**2077**	**4141**	**4931**	**4115**	**cell division protein**	**ftsL**
**SAUSA300_1621**	**0,5**	**3,5E-46**	**10809**	**10482**	**9653**	**5627**	**6256**	**6444**	**13888**	**13909**	**12597**	**ATP-dependent protease ATP-binding subunit ClpX**	**clpX**
**SAUSA300_2549**	**0,4**	**1,4E-04**	**235**	**279**	**275**	**453**	**345**	**371**	**721**	**718**	**1166**	**BCCT family choline/carnitine/betaine transporter**	**cudT**
**SAUSA300_0279**	**0,4**	**6,1E-06**	**2909**	**1549**	**1418**	**1178**	**1053**	**1151**	**2800**	**2193**	**2776**	**EsaA membrane- bound, type VII secretion**	**esaA**
**SAUSA300_1072**	**0,4**	**1,0E-33**	**6564**	**5504**	**6003**	**2147**	**2373**	**2683**	**5883**	**6022**	**5218**	**cell division protein MraZ**	**mraZ**
**SAUSA300_1073**	**0,4**	**2,4E-24**	**11901**	**11564**	**10418**	**4194**	**5864**	**4743**	**12170**	**14178**	**11323**	**S-adenosyl-methyltransferase MraW**	**mraW**
**SAUSA300_0278**	**0,4**	**1,8E-05**	**19024**	**7242**	**6505**	**4059**	**3971**	**3969**	**11019**	**10333**	**10894**	**EsxA toxin (type VII secretion locus)**	**esxA**
**SAUSA300_2546**	**0,3**	**8,5E-03**	**292**	**285**	**205**	**929**	**578**	**636**	**2136**	**1742**	**3911**	**glycine betaine aldehyde dehydrogenase**	**betB**

Supplementary Table 3. Relative expression of ClpX controlled sRNAs in JE2 wild-type, JE2ΔclpX and $JE2_{clpXI265E}$

ID	Fold JE2/ΔclpX	padj	JE2 E1	JE2 E2	JE2 E3	JE2ΔclpX 1	JE2ΔclpX 2	JE2ΔclpX 3	JEclpX$_{I265E}$ 1	JEclpX$_{I265E}$ 2	JEclpX$_{I265E}$ 3	sRNA
SAUSA300s013	6,6	1,42E-08	50	82	99	18	11	8	20	10	17	Lysine riboswitch
SAUSA300s084	5,8	4,34E-26	91848	114374	91523	18698	18016	14320	13395	15217	12404	Teg27
SAUSA300s030	4,4	9,78E-03	19	18	16	5	1	6	9	2	12	sprC
SAUSA300s003	3,9	4,07E-16	552	503	606	112	153	158	338	346	251	T-box riboswitch
SAUSA300s002	3,7	1,46E-27	362	364	346	96	107	84	223	208	172	SAM riboswitch
SAUSA300s168	3,5	4,16E-06	761	363	704	91	184	246	265	222	334	Sau-76
SAUSA300s128	3,4	3,24E-08	185	197	156	71	44	44	64	57	51	ssr63
SAUSA300s041	2,7	2,12E-04	114	141	175	28	50	77	114	130	134	rsaOB
SAUSA300s176	2,5	2,67E-13	2529	3216	2068	1075	1187	902	1147	1191	1054	Sau-26
SAUSA300s281	2,2	1,60E-03	94	76	80	31	38	42	33	33	33	tsr17
SAUSA300s129	2,2	2,38E-12	2462	2376	2073	1007	1017	1090	920	1012	1029	ssr68
SAUSA300s284	2,1	6,62E-04	604	714	397	259	325	225	236	284	211	tsr20
SAUSA300s034	1,9	3,74E-04	1057	1086	1415	720	562	641	853	518	647	sprF3
SAUSA300s285	1,8	3,61E-04	579	391	527	252	270	288	255	225	293	tsr21
SAUSA300s007	1,8	8,49E-03	127	153	132	83	79	66	109	93	63	SAM riboswitch
SAUSA300s181	1,8	1,07E-03	265	288	228	153	134	156	145	169	194	Sau-72
SAUSA300s166	1,7	9,04E-03	2431	1752	1638	864	1504	1062	1309	1413	1006	Teg40as
SAUSA300s065	1,6	9,90E-04	1459	1384	1238	943	831	777	880	933	902	Sau-59
SAUSA300s279	1,5	5,51E-03	2320	2272	1916	1383	1658	1293	1433	1469	1216	tsr15
SAUSA300s173	1,5	6,05E-03	1023	1088	826	694	757	522	550	618	519	Sau-15
SAUSA300s190	1,3	2,02E-03	2170	2148	1755	1581	1512	1409	1121	1145	1208	Sau-6515
SAUSA300s191	0,7	4,36E-03	662	543	528	806	697	836	711	709	703	Sau-6524
SAUSA300s263	0,7	2,02E-03	1125	1080	855	1513	1621	1353	1583	1670	1576	JKD6008sRNA403
SAUSA300s187	0,6	3,61E-04	577	798	593	1132	1038	890	681	716	828	Sau-6405
SAUSA300s006	0,6	2,56E-04	66038	72969	77926	109642	121637	132889	149788	137726	132039	ssrA
SAUSA300s238	0,6	2,96E-04	165	157	135	254	258	256	267	196	225	JKD6008sRNA259
SAUSA300s197	0,6	3,86E-04	106	92	81	184	148	142	130	146	139	Sau-6769
SAUSA300s180	0,6	7,41E-05	1787	1185	1351	2324	2614	2545	3418	3387	2659	Sau-39
SAUSA300s165	0,6	9,78E-03	102	145	149	310	222	180	144	187	158	Teg38as
SAUSA300s182	0,5	1,79E-03	339	181	153	485	368	431	365	394	330	Sau-6079
SAUSA300s024	0,5	6,83E-07	561	615	527	1199	1477	1028	1255	1375	1140	GlmS ribozyme
SAUSA300s140	0,4	1,09E-03	95	86	141	278	139	415	69	75	110	ssr128
SAUSA300s080	0,4	3,77E-04	24	15	23	66	47	56	31	30	35	rsaOV
SAUSA300s164	0,3	8,58E-14	7871	9352	5770	25749	22830	19674	17243	18954	19771	Teg36as
SAUSA300s211	0,3	4,75E-03	9	7	12	30	24	37	18	19	12	JKD6008sRNA073
SAUSA300s117	0,3	9,09E-06	29	16	10	68	59	60	29	40	31	ssr8
SAUSA300s152	0,2	4,19E-06	29	18	24	138	93	103	62	65	81	Teg13
SAUSA300s226	0,0	1,01E-11	4	0	1	128	59	67	16	15	18	JKD6008sRNA173
